# Effect of compound kushen injection on immune function in patients with primary liver cancer: a systematic review and meta-analysis

**DOI:** 10.3389/fphar.2026.1715798

**Published:** 2026-02-19

**Authors:** Yu Xiong, Yutong Cai, Chenxi Li, Qian Yan, Xiongwen Wang, Xiaoying Zhang

**Affiliations:** 1 Department of Tumor, Chongqing Hospital of the First Affiliated Hospital of Guangzhou University of Chinese Medicine, Chongqing, China; 2 Postdoctoral Research Station, Guangzhou University of Chinese Medicine, Guangzhou, China; 5 State Key Laboratory of Traditional Chinese Medicine, The First Affiliated Hospital of Guangzhou University of Chinese Medicine, Guangzhou, China; 6 Department of Tumor, Shenzhen Hospital (Futian) of Guangzhou University of Chinese Medicine, Shenzhen, China; 3 School of Traditional Chinese Medicine, Chongqing University of Chinese Medicine, Chongqing, China; 4 School of Acupuncture and Tuina, Chongqing University of Chinese Medicine, Chongqing, China

**Keywords:** compound kushen injection, immune function, meta-analysis, primary liver cancer, systematic review

## Abstract

**Background:**

Primary liver cancer (PLC) is the third leading cause of cancer mortality worldwide. The heterogeneity of PLC and the complex immunosuppressive microenvironment make it difficult for single treatment regimens to meet patient needs. Therefore, it is crucial to find effective combination treatment strategies that enhance immune function in PLC therapy.

**Objective:**

To systematically evaluate the effect of compound kushen injection (CKI) on enhancing immune function in PLC patients and its role in treatment efficacy and related adverse reactions.

**Methods:**

Relevant Chinese and English electronic databases were searched to include randomized controlled trials (RCTs) assessing the impact of CKI on immune function in PLC patients published before June 2025. The quality of the included studies was assessed using the Cochrane risk of bias assessment tool. Statistical analyses, Sensitivity analysis and publication bias assessment were conducted using Stata 18.0 software.

**Results:**

A total of 2,359 patients from 25 RCTs were included (1,185 in the experimental group and 1,174 in the control group). Compared to conventional treatment alone, the combination of CKI and conventional treatment significantly enhanced immune function, as evidenced by increased CD3^+^ levels, CD4^+^ levels, CD4^+^/CD8^+^ ratio, and natural killer (NK) cell levels, while CD8^+^ levels showed no statistical significance. Additionally, CKI improved the objective response rate (ORR) and disease control rate (DCR), reduced AFP levels, increased KPS scores and the one-year overall survival period, reduced treatment-related adverse reactions, including nausea and vomiting, hepatic dysfunction, myelosuppression, fever, and pain.

**Conclusion:**

Existing evidence suggests that the combined use of CKI and conventional treatment may positively impact immune function, therapeutic effect and treatment-related adverse reactions in patients with PLC. However, the reliability of these conclusions is limited by the absence of disease staging stratification analysis and long-term follow-up data, which are essential for confirming the long-term efficacy and safety of the combined treatment.

**Systematic Review Registration:**

https://www.crd.york.ac.uk/PROSPERO/view/CRD420251112494, identifier CRD420251112494.

## 1 Introduction

Primary liver cancer (PLC) is a common malignant tumor worldwide. According to epidemiological statistics, liver cancer ranks sixth in incidence and third in mortality among malignant tumors globally (Bray et al., 2024). Clinically, PLC mainly includes hepatocellular carcinoma, intrahepatic cholangiocarcinoma, and mixed hepatocellular and cholangiocarcinoma. Hepatocellular carcinoma accounts for about 90% of the overall incidence of PLC (Zheng et al., 2025), making it the main type of liver cancer. Currently, various risk factors, including viral infections, metabolic abnormalities, long-term exposure to carcinogenic toxins, and chronic alcohol consumption, are recognized as major contributors to the development of PLC (Chan et al., 2024). Although surgical procedures (hepatectomy and liver transplantation) are the most effective treatments for early-stage liver cancer patients, early clinical symptoms of liver cancer are often non-specific. Moreover, the tumor exhibits rapid progression and high malignancy. Consequently, most liver cancer patients are already in the advanced stage at the time of diagnosis, making hepatectomy and liver transplantation no longer feasible (Yang et al., 2019). Local treatments are commonly used for intermediate liver cancer, including transarterial chemoembolization (TACE) and transarterial radioembolization; the main therapeutic approaches for advanced liver cancer are systemic therapies, such as targeted therapy and immunotherapy (Hwang et al., 2025). Although these therapies can effectively reduce tumor burden and prolong survival, they are associated with high recurrence and metastasis rates (Wu et al., 2022). Moreover, they often come with severe side effects, including gastrointestinal reactions and liver function damage (Liu Z. et al., 2022; Powell et al., 2020). Therefore, there is still a need to explore safer and more effective treatment modalities in clinical practice: minimizing tumor-induced immune suppression while effectively curbing tumor progression.

The progression and evolution of liver cancer are closely related to immune function. The immune system plays a role in immune surveillance by correctly identifying and eliminating malignant tumor cells. Cellular immunity is the main immune regulatory mechanism against tumors, primarily composed of T lymphocytes and natural killer (NK) cells (Zhang et al., 2024). Among them, T lymphocyte subpopulations mainly include CD3^+^ T cells, CD4^+^ T cells, and CD8^+^ T cells, which are key components of cellular immunity. Although immune surveillance involves complex processes to identify and eliminate cancer cells, HCC employs various strategies to evade detection and destruction by the immune system. The enhanced expression of inhibitory immune checkpoint molecules, particularly the increased levels of PD-L1, leads to exhaustion of T cells through coordinated interactions with PD-1 on their surface, resulting in weakened immune effector responses (Du et al., 2022; Teng et al., 2015). The tumor microenvironment surrounding HCC often exhibits immunosuppressive characteristics, primarily due to the accumulation of regulatory T cells and myeloid-derived suppressor cells, which inhibit effector T cells and NK cells, ultimately impairing the immune response against malignant cells (Ning et al., 2024). The accumulation of these immunosuppressive entities ultimately suppresses the function of effector immune cells. Therefore, enhancing immune responses is a key strategy to improve treatment outcomes and quality of life for patients with primary liver cancer.

Given the immune dysfunction associated with HCC, exploring therapeutic strategies that modulate immune responses is critical. Traditional Chinese medicine (TCM) is a discipline that integrates holistic and differential diagnosis concepts, emphasizing the restoration of balance and the elimination of pathogenic factors. In recent years, TCM has provided new treatment strategies for the clinical management of PLC patients. CKI is an anti-tumor TCM preparation made from extracts of Sophora flavescens Aiton (Kushen) and Heterosmilax japonica Kunth (Baituling). Its effects of clearing heat and dampness, cooling blood and detoxifying, and dispersing masses and relieving pain make it commonly used in clinical practice for cancer pain and bleeding. In China, it is approved by the National Medical Products Administration for use as an adjuvant treatment for cancer (Li S. L. et al., 2025). CKI mainly contains active alkaloids such as matrine, oxymatrine, and sophoridine, which have significant anti-tumor and immune-modulating properties (Yue, 2024). Modern pharmacological studies indicate that CKI induces apoptosis of tumor cells, inhibits cell proliferation and migration, regulates the cell cycle, suppresses tumor invasion and metastasis, inhibits tumor energy metabolism, and modulates immune responses (Hou and Hu, 2019). In the DEN-induced rat liver cancer model, compared with 5-fluorouracil (5-FU) chemotherapy alone, the LVEF and LVAWs in the CKI combined with 5-FU chemotherapy group increased, while the levels of LVEDs, BNP, cTnI, and CK-MB decreased, and the levels of TNF-α, IL-1β, and IL-6 decreased, The levels of CD3^+^, CD4^+^, CD4^+^/CD8^+^, IgG, IgM and IgA increased, The results suggested that CKI could alleviate cardiotoxic injury caused by 5-FU in liver cancer rats and also relieve immunosuppression (Wang et al., 2025). CKI has shown significant effects in improving tumor responses, initiating immune function, and reducing the frequency of adverse reactions (Wang et al., 2022). Tregs possess immunosuppressive functions and mainly play a role in maintaining tumor immune escape and tolerance of the tumor immune microenvironment. The immune function of tumor patients is disrupted by a large number of CD4^+^CD25^+^ Tregs induced by tumor cells, which interfere with the body’s anti-tumor immune response by inhibiting T cell function (Yao et al., 2014). Compound Kushen Injection can effectively reduce the level of CD4^+^CD25^+^ Tregs and their inhibitory effect on T cell immune function. It promotes the recovery of immune system function by increasing the levels of immune factors such as CD4^+^ and CD25^+^, thereby enhancing the anti-tumor efficacy of chemotherapeutic drugs (Zhao et al., 2018). These exciting research findings urgently require a systematic evaluation of CKI’s impact on immune function in HCC patients to provide strong evidence support for clinical application.

Recently, the number of RCTs on the combined use of CKI and biomedicine treatment regimens for PLC has been steadily increasing. Although some previous meta-analyses have indicated that CKI combined with biomedicine treatment regimens is more effective than biomedicine treatment regimens alone, reports comprehensively assessing the effects of CKI combined with conventional treatment regimens on immune function indicators in PLC patients remain limited. Therefore, this study aims to conduct an updated meta-analysis of clinical randomized controlled trials investigating the immune function effects of CKI combined with biomedicine treatment regimens for PLC. The focus will be on key factors related to PLC progression and treatment response. This work is intended to provide a reference for the clinical application of CKI.

## 2 Methods

### 2.1 Study registration

This systematic review and meta-analysis strictly followed the 2020 version of the PRISMA guidelines to ensure methodological transparency and reduce potential bias (Supplementary Table 1). The study protocol has been registered in the PROSPERO database (www.crd.york.ac.uk) with registration number CRD420251112494.

To enhance the accuracy of this study, we standardized the characterization of CKI metabolite in accordance with the Plant Extracts (ConPhyMP) guidelines. Simultaneously, we strictly adhered to established norms for the scientific naming and standardization of botanical drugs metabolites. The CKI formulation analyzed in this study is derived from Sophora flavescens Aiton [Fabaceae; Sophorae Flavescentis Radix] and Heterosmilax japonica Kunth [Smilacaceae; Rhizoma Heterosmilacis Japonicae]. Its taxonomic information has been verified through the Plants of the World Online (POWO) database (http://www.plantsoftheworldonline.org).

### 2.2 Search strategy

Two independent reviewers (XY and XZ) conducted a comprehensive literature search in eight Chinese and English databases. These included PubMed, Embase, Cochrane Library, Web of Science; China National Knowledge Infrastructure (CNKI), Wanfang Database (WF), Chinese Science and Technology Journal Database (VIP), and Chinese Biomedical Literature Database (CBM). The search covered all publications published up to 12 June 2025, without language restrictions. The search strategy was designed based on the PICOS framework, incorporating medical subject headings (MeSH) and free terms such as “compound kushen injection,” “kushen injection,” “Yanshu injection,” “primary liver cancer,” “hepatocellular carcinoma,” and “malignant liver tumors.” Detailed search strategies and results for each database are provided in Supplementary Table 1.

### 2.3 Eligibility criteria

#### 2.3.1 Inclusion criteria

- Participants: Patients diagnosed with primary liver cancer through pathological examination.
- Interventions: The control group received guideline-recommended conventional treatment (CT) regimens (such as interventional therapy, immunotherapy, targeted therapy, and chemotherapy); the treatment group received CKI in addition to the regimen received by the control group.
- Outcomes: Primary outcomes included immune function indicators (CD3^+^, CD4^+^, CD8^+^, CD4^+^/CD8^+^ ratio, and NK cells). Secondary outcome indicators included objective response rate (ORR), disease control rate (DCR), alpha-fetoprotein (AFP), Karnofsky performance status (KPS), one-year survival period, and adverse reactions. Specifically, the assessment of ORR and DCR was based on the World Health Organization (WHO)/RECIST 1.0/RECIST 1.1 criteria for solid tumors. Tumor responses were categorized into four types. Complete response (CR), partial response (PR), stable disease (SD), progressive disease (PD). ORR includes CR and PR, while DCR includes CR, PR, and SD. The KPS is scored according to the Karnofsky performance status, with an increase of 10 points post-treatment indicating improvement, a change (increase or decrease) of less than 10 points indicating stability, and a decrease of more than 10 points indicating deterioration. Clinical adverse reactions in patients include nausea, vomiting, liver function damage, myelosuppression, fever, and pain.
- Study Design: Randomized controlled trials (RCTs).

#### 2.3.2 Exclusion criteria

- Non-RCTs, including animal studies, *in vitro* studies, reviews, case reports, or letters.
- Studies where the treatment group included other botanical drugs in addition to CKI.
- Studies lacking primary outcome data.
- Duplicate publications (only the most comprehensive study with complete data was included).
- Studies for which the full text could not be obtained online or via email.

### 2.4 Study selection and data extraction

EndNote (version 21.2) was used to manage the retrieved studies. After removing duplicate literature, two reviewers (YX and XZ) independently screened titles and abstracts according to pre-established criteria, excluding studies that clearly did not meet the inclusion criteria. The full texts of the remaining studies were then comprehensively reviewed to finalize the included studies. Any discrepancies were resolved through consultation with a senior reviewer (XW) or joint discussion. Two reviewers (YC and CL) independently extracted data using standardized data extraction forms. The extracted data included:

1. Basic information: first author, publication year, study title, and journal;
2. Baseline characteristics: sample size, age, gender, etc.,;
3. Interventions: dosage, duration, frequency of CKI, and conventional treatment regimens;
4. Outcome indicators: all relevant outcomes specified in the studies.

### 2.5 Risk of bias assessment

Two reviewers (YX and QY) independently assessed the quality of the included studies using the Cochrane risk of bias assessment tool, version 2.0 (RoB 2.0) (Cumpston et al., 2019) and the modified Jadad scale (Lunny et al., 2022). The Cochrane risk of bias tool evaluates six domains: randomization method, allocation concealment, blinding of participants, personnel, and outcome assessors, incomplete outcome data, selective reporting, and other biases. Each study was classified as having low, unclear, or high risk of bias. The modified Jadad scale includes four domains: random sequence generation, allocation concealment, blinding, and withdrawals/dropouts, with corresponding scores of 2, 2, 2, and 1, respectively. Trials scoring between 1 and 3 were considered low quality, while those scoring between 4 and 7 were considered high quality. Any discrepancies were resolved through consultation with a third reviewer (XW) or joint discussion.

### 2.6 Statistical analysis

Data analysis was performed using Stata (version 18.0) statistical analysis software. Continuous outcome indicators were expressed as Standard Mean Difference (SMD) with 95% confidence intervals (CIs), while binary outcome indicators were presented as risk ratio (RR) with 95% CIs. A fixed-effect model was used when heterogeneity was low (*I* ^*2*^ < 50% and *p* > 0.05); otherwise, a random-effects model was applied. The level of statistical significance was set at *p* < 0.05.

### 2.7 Sensitivity analysis

Sensitivity analysis was conducted by sequentially excluding individual studies to evaluate the robustness of the results and identify potential sources of heterogeneity. These analyses were performed using Stata (version 18.0).

### 2.8 Subgroup analysis

Subgroup analyses were conducted based on treatment duration (<4 weeks or ≥4 weeks) to explore potential sources of heterogeneity. These analyses were performed using Stata (version 18.0).

### 2.9 Publication bias

Begg’s test and Egger’s test were used to assess publication bias, and funnel plots were used for visual assessment. These analyses were completed using Stata (version 18.0). The trim-and-fill method was performed using Stata (version 15.0).

## 3 Results

### 3.1 Search results and study characteristics

**FIGURE 1:**
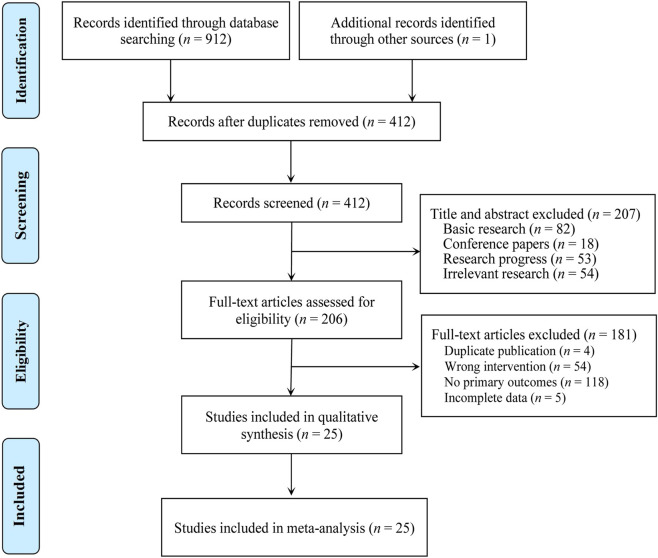
The PRISMA study flowchart of study search.

A total of 913 studies were initially retrieved. After removing duplicate literature using EndNote software, 207 studies were excluded based on the review of titles and abstracts. Then, 206 studies underwent full-text review. Among these, 181 studies were excluded. Finally, 25 studies were included as shown in Figure 1 (Ba, 2018; Cao et al., 2011; Chen et al., 2007; Dong et al., 2016; Guan et al., 2006; Guo, 2015; Han et al., 2012; Hao et al., 2020; He et al., 2016; Jiang et al., 2017; Li, 2020; Li and Wang, 2021; Liu L. Q. et al., 2024; Lu et al., 2011; Lv et al., 2023; Ren and Feng, 2024; Wang, 2009; Xu et al., 2012; Yao et al., 2021; You et al., 2018; Yuan, 2018; Zhang and Zhou, 2014; Zhang et al., 2017; Zhang et al., 2018; Zhou, 2021). All 25 included studies were conducted in China, involving a total of 2,359 patients (1,185 in the experimental group and 1,174 in the control group), published between 2006 and 2024. Sample sizes ranged from 60 to 216 participants, and treatment durations ranged from 4 days to 12 weeks. Regarding treatment regimens, 16 studies (Ba, 2018; Cao et al., 2011; Dong et al., 2016; Guo, 2015; Han et al., 2012; Hao et al., 2020; Li, 2020; Lu et al., 2011; Lv et al., 2023; Ren and Feng, 2024; Wang, 2009; Xu et al., 2012; Yao et al., 2021; You et al., 2018; Yuan, 2018; Zhou, 2021).used CKI combined with TACE; 2 studies (He et al., 2016; Zhang et al., 2017) used CKI combined with Hepatectomy; 2 studies (Zhang and Zhou, 2014; Zhang et al., 2018) used CKI combined with GP regimen; 1 study (Liu L. Q. et al., 2024) used CKI combined with FOLFOX4 regimen; 1 study (Guan et al., 2006) involved CKI combined with interventional Therapy or chemotherapy; 1 study (Chen et al., 2007) used CKI combined with TACE combined with anhydrous ethanol injection; 1 study (Jiang et al., 2017) involved CKI combined with radioactive ^125^I particle implantation; and 1 study (Li and Wang, 2021) used CKI combined with TACE plus RFA. Table 1 summarizes the characteristics and treatment regimens of the included studies.

**TABLE 1:** Included studies basic characteristics.

Study ID	Sample size		Sex (M/F)		Mean age (years)		Interventions		Criteria	Treatment duration	Outcomes
	T	C	T	C	T	C	T	C			
Ba (2018)	42	42	23/19	25/17	56.8 ± 3.1	56.3 ± 2.9	CT + CKI, 20 mL	CT (TACE)	RECIST1.1	12w	②③④⑥⑦⑩
Cao et al. (2011)	30	30	—	—	—	—	CT + CKI, 20 mL, qd	CT (TACE)	RECIST1.0	12w	① ②④⑤⑥⑦⑨
Chen et al. (2007)	46	40	62/24		40∼71		CT + CKI, 20 mL, qd	CT (TACE + AEI)	WHO	6w-9w	① ②③④⑤⑥⑦⑨⑩⑪
Dong et al. (2016)	108	108	137/79		56		CT + CKI, 20 mL	CT (TACE)	WHO	14d-20d	① ②③④⑤⑥⑦
Guan et al. (2006)	93	92	120/65		26∼65		CT + CKI, 20 mL, qd	CT (IT/Chemo)	WHO	45d-60d	① ②③④⑤⑥⑦⑨⑪
Guo (2015)	78	78	45/33	47/31	46.1 ± 3.2	47.3 ± 2.9	CT + CKI, 20 mL	CT (TACE)	—	12w	① ②③④⑤
Han et al. (2012)	30	30	19/11	18/12	56	57	CT + CKI, 15 mL	CT (TACE)	WHO	4d	① ②③④⑥⑦⑨⑩
Hao et al. (2020)	49	49	29/20	31/18	57.01 ± 6.82	56.25 ± 6.21	CT + CKI, 20 mL, qd	CT (TACE)	RECIST1.1	30d-80d	②③④⑤⑥⑦⑧
He et al. (2016)	40	49	25/15	31/18	56.20 ± 5.16	55.15 ± 9.18	CT + CKI, 20 mL, qd	CT (Hepatectomy)	—	10d	②③④⑤
Jiang et al. (2017)	46	46	27/19	28/18	52.8	50.6	CT + CKI, 20 mL, qd	CT (RPI)	RECIST1.1	14d-21d	① ②③④⑥⑦⑧⑨
Li (2020)	33	31	20/13	18/13	61.38 ± 6.85	60.74 ± 7.29	CT + CKI, 12 mL, qd	CT (TACE)	WHO	4w	②③⑥⑦⑨
Li and Wang (2021)	32	32	20/12	18/14	38.42 ± 5.12	36.46 ± 4.35	CT + CKI, 20 mL, qd	CT (TACE + RFA)	mRECIST	2w	②③④⑤⑥⑦⑧⑨
Liu L. Q. et al. (2024)	30	30	19/11	17/13	59.88 ± 4.71	59.65 ± 4.66	CT + CKI, 20 mL, qd	CT (FOLFOX4)	RECIST1.0	30d	②③⑤⑥⑦⑧
Lu et al. (2011)	39	35	25/14	22/13	63.4	62.8	CT + CKI, 20 mL	CT (TACE)	—	10d	①②③④⑤⑩
Lv et al., 2023	53	53	27/26	28/25	58.28 ± 8.89	56.21 ± 10.64	CT + CKI, 20 mL, qd	CT (TACE)	iRECIST	20d	①②③④⑥⑦⑧⑩
Ren and Feng (2024)	52	52	27/25	29/23	59.79 ± 6.33	59.62 ± 7.28	CT + CKI, 20 mL, qd	CT (TACE)	WHO	4w	① ②④⑥⑦⑩⑪
Wang (2009)	42	41	51/32		53		CT + CKI, 20 mL	CT (TACE)	WHO	20d	① ②③⑤⑥⑦
Xu et al. (2012)	30	30	38/22		53		CT + CKI, 20 mL	CT (TACE)	WHO	30d	①②④⑤⑥⑦⑨⑪
Yao et al. (2021)	48	48	25/23	23/25	63.24 ± 2.57	63.58 ± 2.96	CT + CKI, 20 mL	CT (TACE)	RECIST1.0	30d	① ②③④⑤⑥⑦
You et al. (2018)	45	45	29/16	26/19	61.56 ± 2.94	61.92 ± 2.25	CT + CKI, 20 mL, qd	CT (TACE)	WHO	20d	②③④⑥⑦⑧
Yuan (2018)	45	45	23/22	25/20	60.41 ± 8.43	60.84 ± 8.67	CT + CKI, 20 mL	CT (TACE)	—	10d	①②④⑨⑩
Zhang and Zhou (2014)	48	42	27/21	26/16	63.8 ± 8.2	64.2 ± 7.8	CT + CKI, 30 mL, qd	CT (GP)	WHO	8w	①②③④⑤⑥⑦ ⑩
Zhang et al. (2017)	48	48	28/20	26/22	52.65 ± 10.21	52.34 ± 10.24	CT + CKI, 15 mL, qd	CT (Hepatectomy)	—	4w	① ②④⑧
Zhang et al. (2018)	36	36	22/14	23/13	50.12 ± 11.85	49. 62 ± 12. 07	CT + CKI, 30 mL, qd	CT (GP)	WHO	8w	①②③④⑥⑩
Zhou (2021)	42	42	27/15	25/17	58.44 ± 4.62	57.62 ± 4.71	CT + CKI, 20 mL, qd	CT (TACE)	mRECIST	20d	① ②④⑥⑦⑩

Abbreviations: C, control group; T, treatment group; M, male; F, female; qd, quaque in die; bid, bis in die; CKI, compound kushen injection; CT, conventional treatment; TACE, transarterial chemoembolization; AEI, anhydrous ethanol injection; IT/Chemo, Interventional Therapy/chemotherapy; RPI, radioactive 125I particle implantation; RFA, radiofrequency ablation; FOLFOX4, Oxaliplatin + Fluorouracil + Folinic acid; GP, Gemcitabine + Cisplatin; Outcomes: ①CD3+; ②CD4+; ③CD8+; ④CD4+/CD8+; ⑤NKcell; ⑥ORR; ⑦DCR; ⑧AFP; ⑨KPS; ⑩Adverse reactions; ⑪ One-Year Survival Rate; -, not reported.

### 3.2 Risk of bias assessment

**FIGURE 2:**
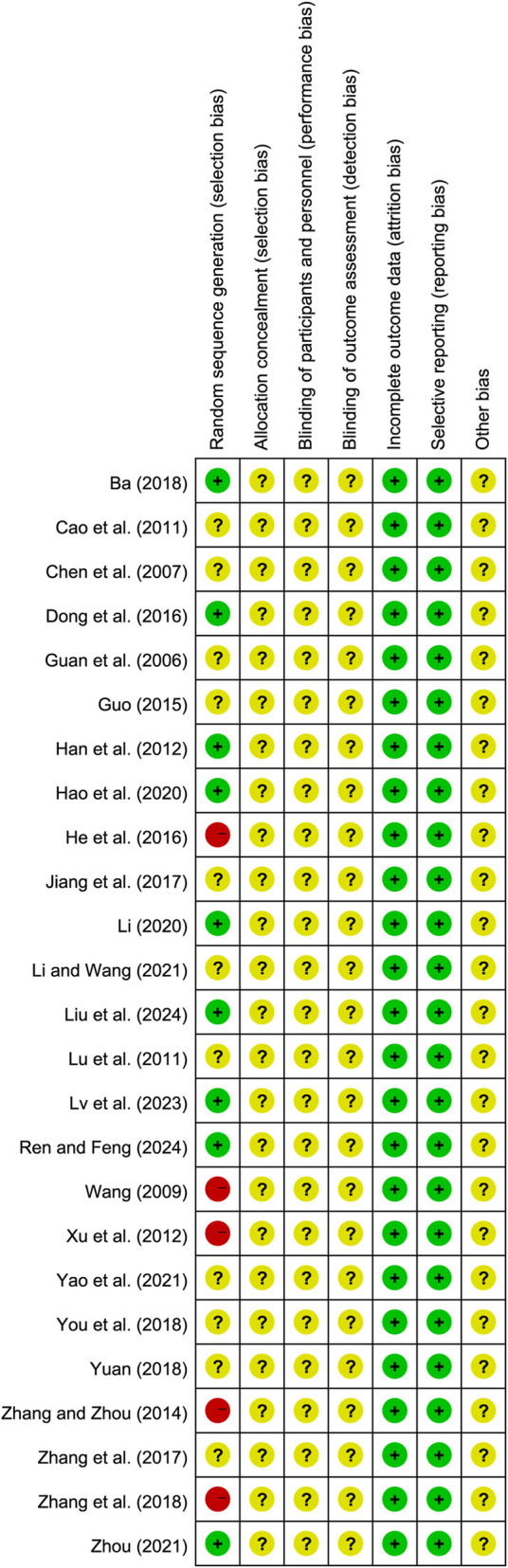
Bias risk assessment of included studies.

All studies reported baseline comparability. Quality assessment of the included studies was conducted using RoB 2.0 and the modified Jadad scale. Among them, 9 studies (Ba, 2018; Dong et al., 2016; Han et al., 2012; Hao et al., 2020; Li, 2020; Liu L. Q. et al., 2024; Lv et al., 2023; Ren and Feng, 2024; Zhou, 2021) were assessed as low risk due to the explicit use of random number tables for grouping. 5 studies (He et al., 2016; Wang, 2009; Xu et al., 2012; Zhang and Zhou, 2014; Zhang et al., 2018) were assessed as high risk: 1 study (Zhang and Zhou, 2014) grouped by treatment methods, 3 studies (Wang, 2009; Xu et al., 2012; Zhang et al., 2018) grouped by admission order, and 1 study (He et al., 2016) grouped by patient willingness. 11 studies (Cao et al., 2011; Chen et al., 2007; Guan et al., 2006; Guo, 2015; Jiang et al., 2017; Li and Wang, 2021; Lu et al., 2011; Yao et al., 2021; You et al., 2018; Yuan, 2018; Zhang et al., 2017) did not clearly report randomization methods and were thus classified as unclear risk. None of the studies reported the use of blinding or allocation concealment; consequently, the risk ratings in these areas are unclear. All studies reported no loss to follow-up, resulting in a low risk assessment for the completeness of outcome data. Additionally, the definitions of outcome indicators were clear and comprehensively reported across studies, with low risk assessments for selective reporting. Due to the lack of explicit reporting on other potential sources of bias, the overall risk rating for other biases remains unclear. Overall, the methodological quality of the included studies is relatively low. The results of the risk of bias assessment are detailed in Figure 2; Supplementary Table 1.

### 3.3 Primary outcomes

#### 3.3.1 CD3^+^ levels

**FIGURE 3:**
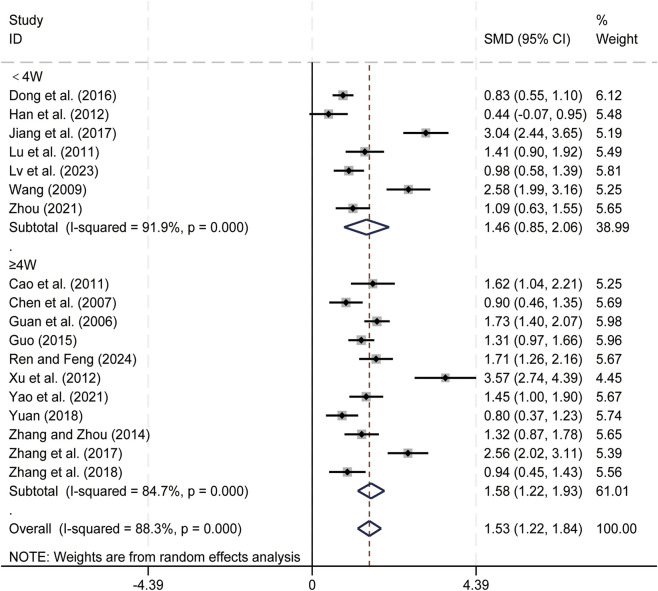
Forest plot for CD3^+^ levels.

**FIGURE 4:**
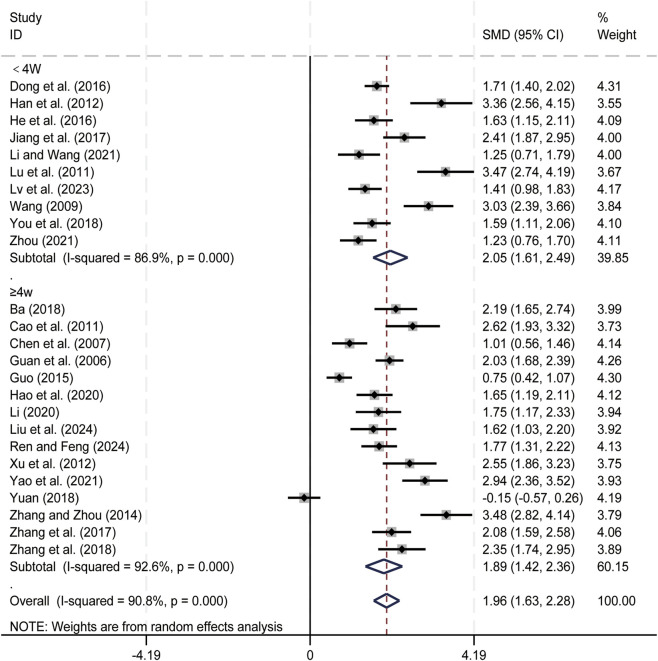
Forest plot for CD4^+^ levels.

18 studies (Cao et al., 2011; Chen et al., 2007; Dong et al., 2016; Guan et al., 2006; Guo, 2015; Han et al., 2012; Jiang et al., 2017; Lu et al., 2011; Lv et al., 2023; Ren and Feng, 2024; Wang, 2009; Xu et al., 2012; Yao et al., 2021; Yuan, 2018; Zhang and Zhou, 2014; Zhang et al., 2017; Zhang et al., 2018; Zhou, 2021) assessed CD3^+^ levels. Data analysis was performed using Stata (version 18.0) statistical analysis software. Due to significant heterogeneity (*p < 0.05, I* ^*2*^ = 88.3%), a random-effects model was used to summarize the effect sizes. The main sources of heterogeneity were clinical differences among studies, including variations in liver cancer staging and treatment regimens. Compared to conventional treatment (CT) alone, CKI combined with CT significantly enhanced CD3^+^ levels [SMD = 1.53, 95% CI: 1.22 to 1.84, Figure 3], Subgroup analysis based on treatment duration showed significant improvements in CD3^+^ levels for CKI combined with CT in both duration categories: less than 4 weeks [SMD = 1.46, 95% CI: 0.85 to 2.06, Figure 3] and 4 weeks or longer [SMD = 1.58, 95% CI: 1.22 to 1.93, Figure 3]. We have conducted subgroup analyses of CD3^+^ based on different treatment regimens (Supplementary File 1) and CKI dose (Supplementary File 2), CD3^+^ is an important immune cell in the body, the suggests that the combined regimen can effectively enhance patients’ immune function.

#### 3.3.2 CD4^+^ levels

25 studies (Ba, 2018; Cao et al., 2011; Chen et al., 2007; Dong et al., 2016; Guan et al., 2006; Guo, 2015; Han et al., 2012; Hao et al., 2020; He et al., 2016; Jiang et al., 2017; Li, 2020; Li and Wang, 2021; Liu L. Q. et al., 2024; Lu et al., 2011; Lv et al., 2023; Ren and Feng, 2024; Wang, 2009; Xu et al., 2012; Yao et al., 2021; You et al., 2018; Yuan, 2018; Zhang and Zhou, 2014; Zhang et al., 2017; Zhang et al., 2018; Zhou, 2021) evaluated CD4^+^ levels. Data analysis was performed using Stata (version 18.0) statistical analysis software. Due to significant heterogeneity (*p < 0.05, I* ^*2*^ = 90.8%), a random-effects model was used to summarize the effect sizes. The main sources of heterogeneity were clinical differences among studies, including variations in liver cancer staging and treatment regimens. Compared with CT alone, CKI combined with CT significantly increased CD4^+^ levels (SMD = 1.96, 95% CI: 1.63 to 2.28, Figure 4). Subgroup analysis based on treatment duration showed significant improvements in CD4^+^ levels for both durations: less than 4 weeks (SMD = 2.05, 95% CI: 1.61 to 2.49, Figure 4) and 4 weeks or longer (SMD = 1.89, 95% CI: 1.42 to 2.36, Figure 4). We have conducted subgroup analyses of CD4^+^ based on different treatment regimens (Supplementary File 1) and CKI dose (Supplementary File 2). CD4^+^ is an important immune cell in the body, the result suggests that the combined regimen can effectively enhance patients’ immune function.

#### 3.3.3 CD8^+^ levels

**FIGURE 5:**
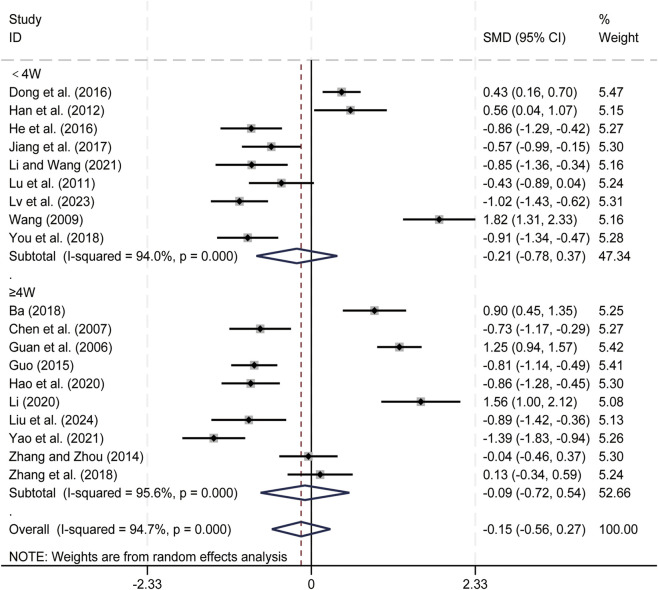
Forest plot for CD8^+^ levels.

19 studies (Ba, 2018; Chen et al., 2007; Dong et al., 2016; Guan et al., 2006; Guo, 2015; Han et al., 2012; Hao et al., 2020; He et al., 2016; Jiang et al., 2017; Li, 2020; Li and Wang, 2021; Liu L. Q. et al., 2024; Lu et al., 2011; Lv et al., 2023; Wang, 2009; Yao et al., 2021; You et al., 2018; Zhang and Zhou, 2014; Zhang et al., 2018) evaluated CD8^+^ levels. Data analysis was performed using Stata (version 18.0) statistical analysis software. Due to significant heterogeneity (*p < 0.05, I* ^*2*^ = 94.7%), a random-effects model was used to summarize the effect sizes. The main sources of heterogeneity were clinical differences among studies, including variations in liver cancer staging and treatment regimens. Compared to CT alone, CKI combined with CT did not significantly affect CD8^+^ levels (SMD = −0.15, 95% CI: −0.56 to 0.27, *p* = 0.495, Figure 5). Further subgroup analysis based on treatment duration indicated that CKI combined with CT did not result in significant changes in CD8^+^ levels for either duration: less than 4 weeks (SMD = −0.21, 95% CI: −0.78 to 0.37, *p* = 0.484, Figure 5) and 4 weeks or longer (SMD = −0.09, 95% CI: −0.72 to 0.54, *p* = 0.779, Figure 5). We have conducted subgroup analyses of CD8^+^ based on different treatment regimens (Supplementary File 1) and CKI dose (Supplementary File 2). CD8^+^ T cells affect the immune function of PLC patients in a bidirectional feedback manner, and the lack of statistical significance may be related to this result.

#### 3.3.4 CD4^+^/CD8^+^ ratio

**FIGURE 6:**
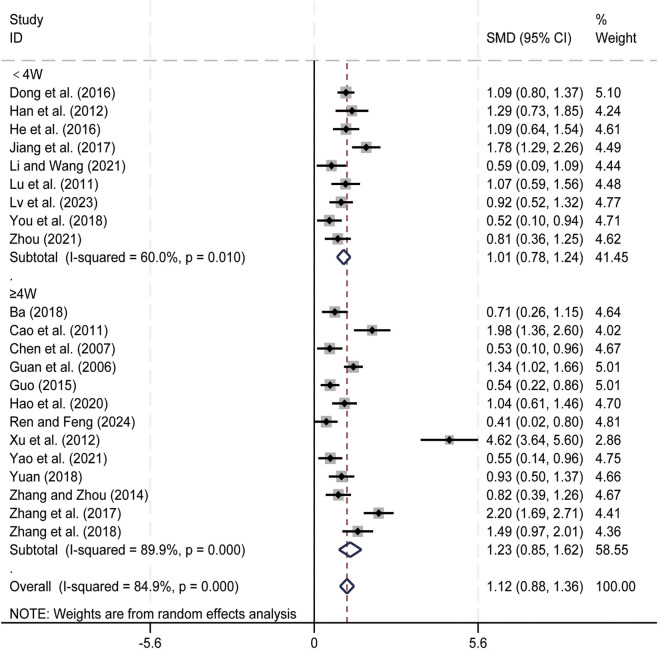
Forest plot for CD4^+^/CD8^+^ levels.

22 studies (Ba, 2018; Cao et al., 2011; Chen et al., 2007; Dong et al., 2016; Guan et al., 2006; Guo, 2015; Han et al., 2012; Hao et al., 2020; He et al., 2016; Jiang et al., 2017; Li and Wang, 2021; Lu et al., 2011; Lv et al., 2023; Ren and Feng, 2024; Xu et al., 2012; Yao et al., 2021; You et al., 2018; Yuan, 2018; Zhang and Zhou, 2014; Zhang et al., 2017; Zhang et al., 2018; Zhou, 2021) evaluated CD4^+^/CD8^+^ ratio. Data analysis was performed using Stata (version 18.0) statistical analysis software. Due to significant statistical heterogeneity (*p < 0.05, I* ^*2*^ = 84.9%), a random-effects model was used to summarize the effect size. The main sources of heterogeneity were clinical differences among studies, including variations in liver cancer staging and treatment regimens. Compared to CT alone, CKI combined with CT significantly improved CD4^+^/CD8^+^ ratio (SMD = 1.12, 95% CI: 0.88 to 1.36, Figure 6). Subgroup analysis based on treatment duration showed significant improvement in CD4^+^/CD8^+^ ratio with CKI combined with CT for both duration groups: less than 4 weeks (SMD = 1.01, 95% CI: 0.78 to 1.24, Figure 6) and 4 weeks or longer (SMD = 1.23, 95% CI: 0.85 to 1.62, Figure 6). We have conducted subgroup analyses of CD4^+^/CD8^+^ ratio based on different treatment regimens (Supplementary File 1) and CKI dose (Supplementary File 2). The CD4^+^/CD8^+^ ratio serves as an important indicator reflecting the immune regulation of the body, the result suggests that the combined regimen can effectively enhance patients’ immune function.

#### 3.3.5 NK cell levels

**FIGURE 7:**
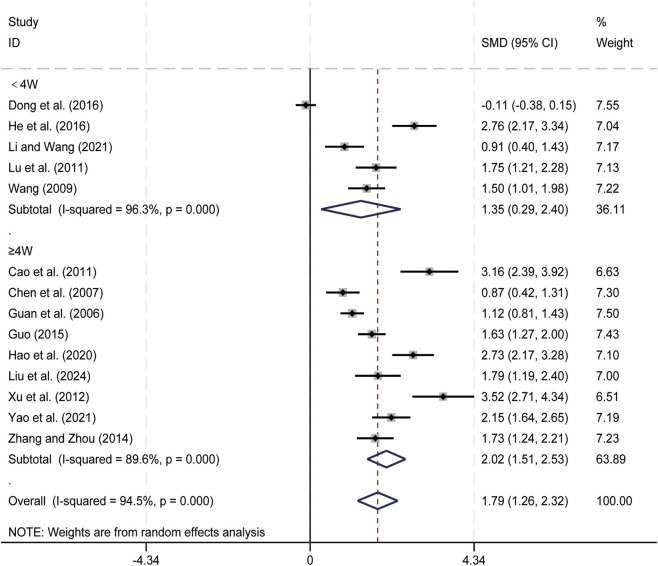
Forest plot for NK cell levels.

14 studies (Cao et al., 2011; Chen et al., 2007; Dong et al., 2016; Guan et al., 2006; Guo, 2015; Hao et al., 2020; He et al., 2016; Li and Wang, 2021; Liu L. Q. et al., 2024; Lu et al., 2011; Wang, 2009; Xu et al., 2012; Yao et al., 2021; Zhang and Zhou, 2014) evaluated NK cell levels. Data analysis was performed using Stata (version 18.0) statistical analysis software. Due to significant statistical heterogeneity (*p < 0.05, I* ^*2*^ = 94.5%), a random-effects model was used to summarize the effect sizes. The main sources of heterogeneity were clinical differences among studies, including variations in liver cancer staging and treatment regimens. Compared to CT alone, CKI combined with CT significantly increased NK cell levels (SMD = 1.79, 95% CI: 1.26 to 2.32, Figure 7). Subgroup analysis based on treatment duration showed significant improvement in NK cell levels with CKI combined with CT for both treatment durations: less than 4 weeks (SMD = 1.35, 95% CI: 0.29 to 2.40, *p* = 0.012, Figure 7) and 4 weeks or longer (SMD = 2.02, 95% CI: 1.51 to 2.53, Figure 7). We have conducted subgroup analyses of NK cell based on different treatment regimens (Supplementary File 1) and CKI dose (Supplementary File 2). NK cell is an important immune cell in the body, the result suggests that the combined regimen can effectively enhance patients’ immune function.

### 3.4 Secondary outcomes

#### 3.4.1 ORR

**FIGURE 8:**
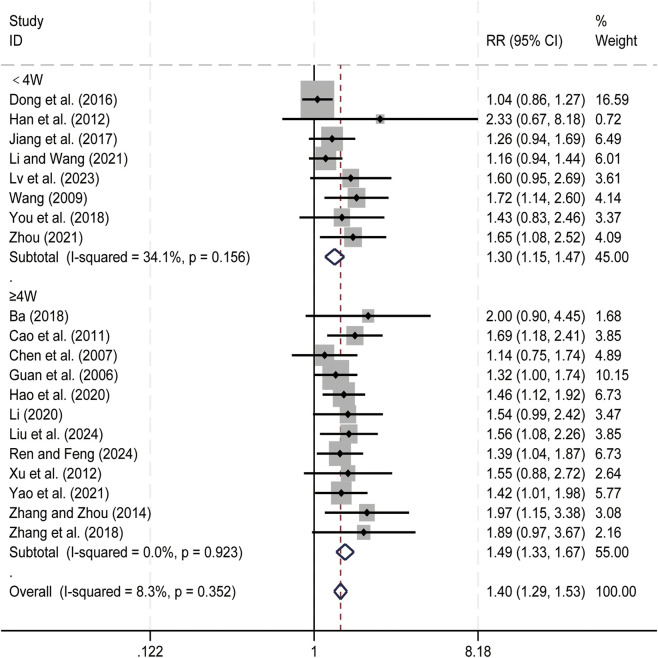
Forest plot for ORR.

20 studies (Ba, 2018; Cao et al., 2011; Chen et al., 2007; Dong et al., 2016; Guan et al., 2006; Han et al., 2012; Hao et al., 2020; Jiang et al., 2017; Li, 2020; Li and Wang, 2021; Liu L. Q. et al., 2024; Lv et al., 2023; Ren and Feng, 2024; Wang, 2009; Xu et al., 2012; Yao et al., 2021; You et al., 2018; Zhang and Zhou, 2014; Zhang et al., 2018; Zhou, 2021) evaluated ORR. Data analysis was performed using Stata (version 18.0) statistical analysis software. Due to low heterogeneity (*p* = 0.352, *I* ^*2*^ = 8.3%), a fixed-effects model was used to summarize the effect size. Compared to CT alone, CKI combined with CT significantly improved ORR [RR = 1.40, 95% CI: 1.29 to 1.53, Figure 8]. Subgroup analysis based on treatment duration showed significant improvement in ORR with CKI combined with CT for both treatment durations: less than 4 weeks (RR = 1.30, 95% CI: 1.15 to 1.47, Figure 8) and ≥4 weeks (RR = 1.49, 95% CI: 1.33 to 1.67, Figure 8), We have conducted subgroup analyses of ORR cell based on different treatment regimens (Supplementary File 1). This suggests that CKI can improve the clinical efficacy of PLC patients to a certain extent.

#### 3.4.2 DCR

**FIGURE 9:**
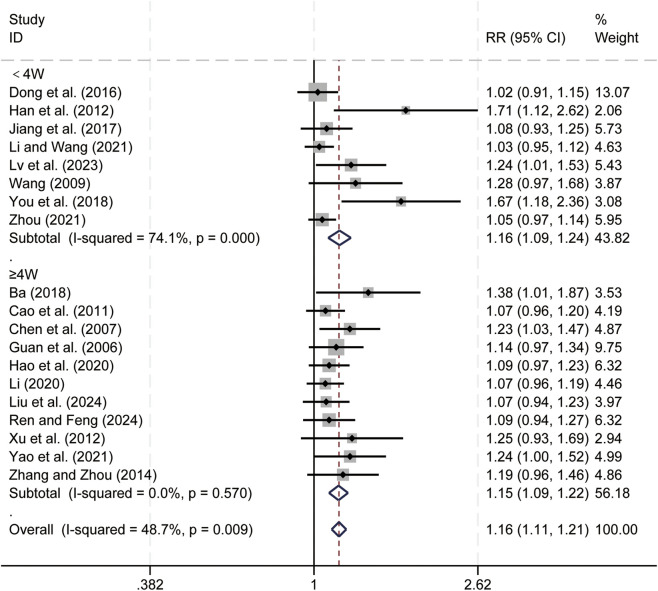
Forest plot for DCR.

19 studies (Ba, 2018; Cao et al., 2011; Chen et al., 2007; Dong et al., 2016; Guan et al., 2006; Han et al., 2012; Hao et al., 2020; Jiang et al., 2017; Li, 2020; Li and Wang, 2021; Liu L. Q. et al., 2024; Lv et al., 2023; Ren and Feng, 2024; Wang, 2009; Xu et al., 2012; Yao et al., 2021; You et al., 2018; Zhang and Zhou, 2014; Zhou, 2021) evaluated DCR. Data analysis was performed using Stata (version 18.0) statistical analysis software. Due to low heterogeneity (*p* = 0.009, *I* ^*2*^ = 48.7%), a fixed-effects model was used to summarize the effect size. Compared to CT alone, CKI combined with CT significantly improved DCR (RR = 1.16, 95% CI: 1.11 to 1.21, Figure 9). Subgroup analysis based on treatment duration showed significant improvement in DCR with CKI combined with CT for both treatment durations: less than 4 weeks (RR = 1.16, 95% CI: 1.09 to 1.24, Figure 9) and 4 weeks or longer (RR = 1.15, 95% CI: 1.09 to 1.22, Figure 9), We have conducted subgroup analyses of DCR cell based on different treatment regimens (Supplementary File 1). This suggests that CKI can improve the clinical efficacy of PLC patients to a certain extent.

#### 3.4.3 AFP levels

**FIGURE 10:**
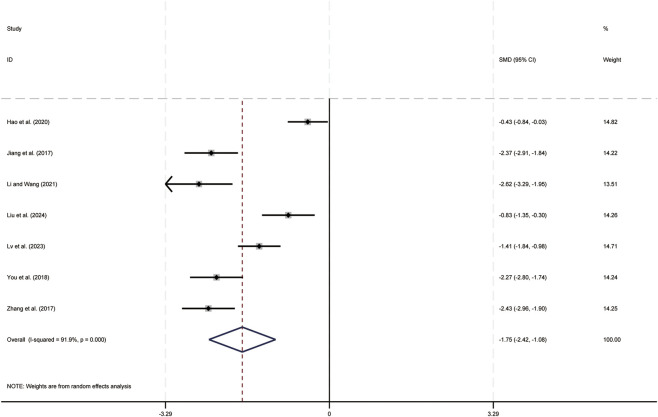
Forest plot for AFP levels.

Seven studies (Hao et al., 2020; Jiang et al., 2017; Li and Wang, 2021; Liu L. Q. et al., 2024; Lv et al., 2023; You et al., 2018; Zhang et al., 2017) evaluated AFP levels. Data analysis was performed using Stata (version 18.0) statistical analysis software. Due to significant statistical heterogeneity (*p < 0.05, I* ^*2*^ = 91.9%), a random-effects model was used to summarize the effect size. The main sources of heterogeneity were clinical differences among studies, including variations in liver cancer staging and treatment regimens. Compared to CT alone, CKI combined with CT significantly reduced AFP levels (SMD = −1.75, 95% CI: −2.42 to −1.08, Figure 10), this indicates that CKI has certain therapeutic advantages in inhibiting the development of liver tumors.

#### 3.4.4 KPS scores

**FIGURE 11:**
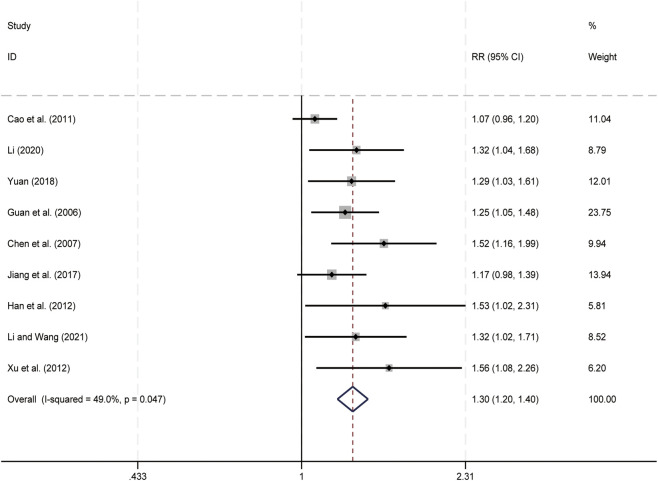
Forest plot for KPS Scores.

Nine studies (Cao et al., 2011; Chen et al., 2007; Guan et al., 2006; Han et al., 2012; Jiang et al., 2017; Li, 2020; Li and Wang, 2021; Xu et al., 2012; Yuan, 2018) evaluated KPS scores. Data analysis was performed using Stata (version 18.0) statistical analysis software. Due to low heterogeneity (*p* = 0.047, *I* ^*2*^ = 49.0%), a fixed-effects model was used to summarize the effect size. Compared to CT alone, CKI combined with CT significantly improved KPS scores (RR = 1.30, 95% CI: 1.20 to 1.40, Figure 11), this indicates that CKI can improve patients’ quality of life.

#### 3.4.5 One-year survival period

**FIGURE 12:**
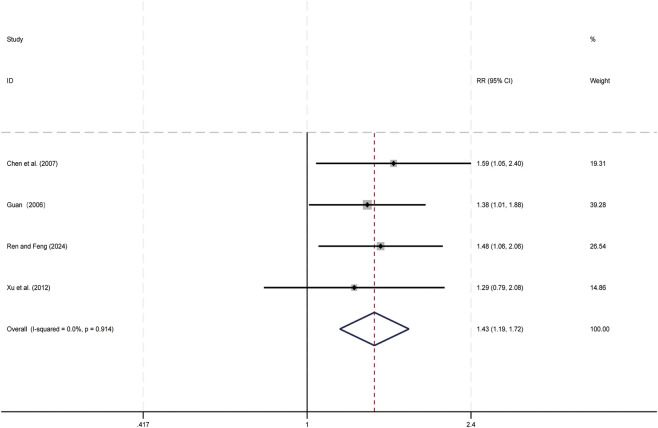
Forest plot for One-Year Survival period.

Four studies (Chen et al., 2007; Guan et al., 2006; Ren and Feng, 2024; Xu et al., 2012) evaluated the one-year survival period. Data analysis was performed using Stata (version 18.0) statistical analysis software. Due to low heterogeneity (*p* = 0.914, *I* ^*2*^ = 0.0%), a fixed-effects model was used to summarize the effect size. Compared to CT alone, CKI combined with CT significantly improved the one-year survival period (RR = 1.43, 95% CI: 1.19 to 1.721, Figure 12), this suggests that CKI can improve the clinical efficacy of PLC patients.

#### 3.4.6 Adverse reactions

The adverse reactions of CKI treatment for PLC included nausea and vomiting, hepatic dysfunction, myelosuppression, fever, and pain. Seven studies evaluated nausea and vomiting, six studies evaluated hepatic dysfunction, nine studies evaluated myelosuppression, eight studies evaluated fever, and seven studies evaluated pain. Data analysis was performed using Stata (version 18.0) statistical analysis software. Compared to CT alone, CKI combined with CT significantly reduced treatment-related adverse reactions: nausea and vomiting (RR = 0.52, 95% CI: 0.40 to 0.69], hepatic dysfunction (RR = 0.31, 95% CI: 0.20 to 0.49), myelosuppression (RR = 0.36, 95% CI: 0.27 to 0.48), fever (RR = 0.44, 95% CI: 0.32 to 0.61), and pain (RR = 0.52, 95% CI: 0.39 to 0.69). Details are in Table 2. We have conducted subgroup analyses of adverse reactions cell based on different treatment regimens (Supplementary File 1).

**TABLE 2:** Adverse reactions of the included studies.

Adverse reactions	Included studies	Treantment group		Control group		Heterogeneity		Outcomes	
		Events	Total	Events	Total	I^2^ (%)	*P*	RR (M-H,Fixed, 95%CI)	*P*
Nausea and vomiting	Ba (2018); Ren and Feng (2024); Lu et al. (2011); Lv et al. (2023); Zhang and Zhou (2014); Zhang et al. (2018); Zhou (2021)	43	312	87	302	0	0.706	0.52 [0.40,0.69]	0.000
Hepatic dysfunction	Ba (2018); Chen et al. (2007); Lu et al. (2011); Yuan (2018); Zhang and Zhou (2014); Zhang et al. (2018)	21	256	62	240	0	0.911	0.31 [0.20,0.49]	0.000
Myelosuppression	Ba (2018); Chen et al. (2007); Han et al. (2012); Lu et al. (2011); Lv et al. (2023); Yuan (2018); Zhang and Zhou (2014); Zhang et al. (2018); Zhou (2021)	50	381	134	365	35.7	0.132	0.36 [0.27,0.48]	0.000
Fever	Ba (2018); Lu et al. (2011); Lv et al. (2023); Ren and Feng (2024); Yuan (2018); Zhang and Zhou (2014); Zhang et al. (2018); Zhou (2021)	41	357	90	347	0	0.68	0.44 [0.32,0.61]	0.000
Pain	Ba (2018); Lu et al. (2011); Ren and Feng (2024); Yuan (2018); Zhang and Zhou (2014); Zhang et al. (2018); Zhou (2021)	53	304	99	294	0	0.993	0.52 [0.39,0.69]	0.000

### 3.5 Sensitivity analysis

**FIGURE 13:**
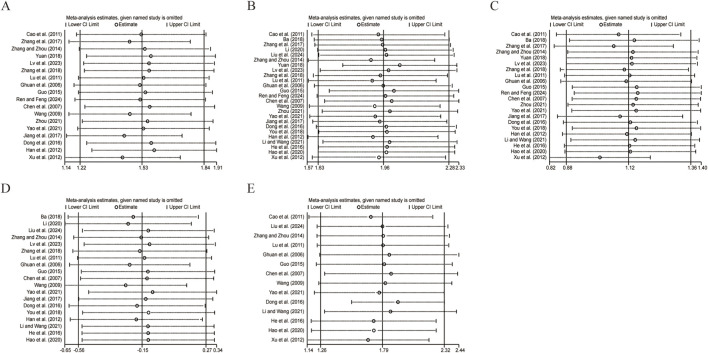
The results of sensitivity analysis. **(A)** CD3^+^ levels. **(B)** CD4^+^ levels. **(C)** CD8^+^ levels. **(D)** CD4^+^/CD8^+^ levels. **(E)** NK cell levels.

To assess the robustness and reliability of the study results, we conducted a sensitivity analysis on the primary outcome indicators, including CD3^+^, CD4^+^, CD8^+^, CD4^+^/CD8^+^ ratio, and NK cell levels. Data analysis was performed using Stata (version 18.0) statistical analysis software. By sequentially excluding each study for each indicator, we recombined the effect sizes after each exclusion and compared these results with the meta-analysis results before exclusion. This approach allowed us to evaluate the impact of individual studies on the overall findings and the robustness of the results. The analysis showed that after sequentially excluding each study, there were no significant changes in the results of the primary and secondary indicators, indicating that the results were not sensitive to the exclusion of any single study and thus confirming the robustness and reliability of the study findings (Figure 13). Sensitivity analyses for ORR and DCR are presented in Supplementary File 3.

### 3.6 Publication bias

**FIGURE 14:**
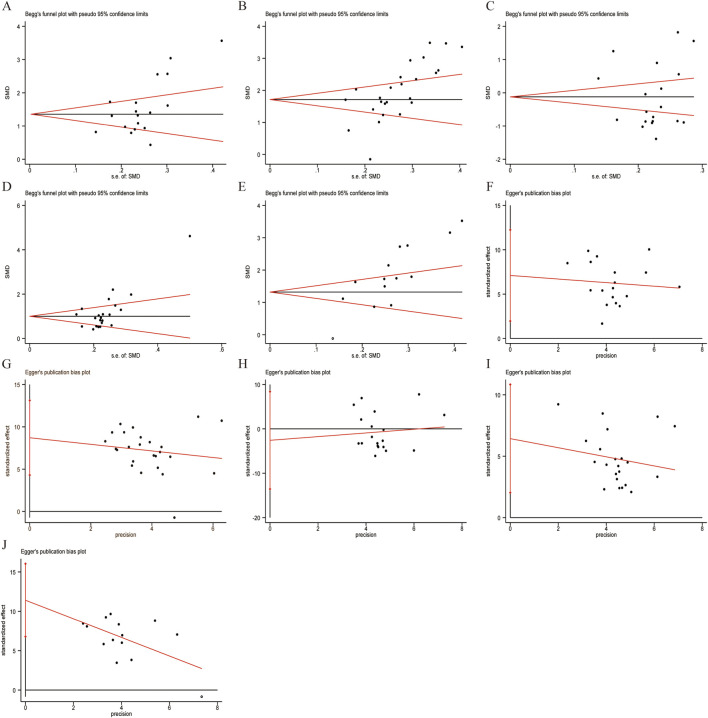
Begg’s and Egger’s publication funnel plot. **(A)** Begg’s test for CD3^+^. **(B)** Begg’s test for CD4^+^. **(C)** Begg’s test for CD8^+^. **(D)** Begg’s test for CD4^+^/CD8^+^. **(E)** Begg’s test for NK cell. **(F)** Egger’s test for CD3^+^. **(G)** Egger’s test for CD4^+^. **(H)** Egger’s test for CD8^+^. **(I)** Egger’s test for CD4^+^/CD8^+^. **(J)** Egger’s test for NK cell.

Given the significant heterogeneity observed in the primary results, Egger’s test and Begg’s test were conducted to assess potential publication bias (Figure 14). Data analysis was performed using Stata (version 18.0) statistical analysis software. The results of Egger’s test showed: CD3^+^ levels (*p* = 0.01), CD4^+^ levels (*p* = 0.000), CD8^+^ levels (*p* = 0.627), CD4^+^/CD8^+^ ratio (*p* = 0.006), and NK cell levels (*p* = 0.000). These results indicate that the primary outcome indicators suggest potential publication bias. The results of Begg’s test showed: CD3^+^ levels (p = 0.023), CD4^+^ levels (*p* = 0.000), CD8^+^ levels (*p* = 0.294), CD4^+^/CD8^+^ ratio (*p* = 0.005), and NK cell levels (*p* = 0.012). These results indicate that the primary outcome indicators suggest potential publication bias.

**FIGURE 15:**
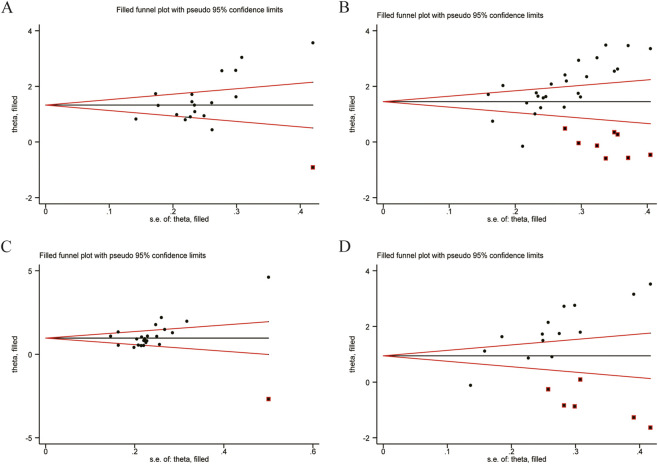
Filled funnel plot of Main indicators. **(A)** CD3^+^ levels. **(B)** CD4^+^ levels. **(C)** CD4^+^/CD8^+^ levels. **(D)** NK cell levels.

To address publication bias, this study used the trim-and-fill method to assess the stability of the combined results (Figure 15). Data analysis was performed using Stata (version 15.0) statistical analysis software. After including data from one imputed study for CD3^+^, a meta-analysis of all studies was conducted again. The heterogeneity test showed Q = 174.581, *p* < 0.001. Using a random-effects model, the combined effect size was 1.874, with a 95% CI of (1.185, 3.000). After including data from eight imputed studies for CD4^+^, a meta-analysis of all studies was conducted again. The heterogeneity test showed Q = 461.286, *p* < 0.001. Using a random-effects model, the combined effect size was 4.410, with a 95% CI of (3.122, 6.227). After including data from one imputed study for CD4^+^/CD8^+^ ratio, a meta-analysis of all studies was conducted again. The heterogeneity test showed Q = 192.861, *p* < 0.001. Using a random-effects model, the combined effect size was 2.772, with a 95% CI of (2.106, 3.649). After including data from six imputed studies for NK cells, a meta-analysis of all studies was conducted again. The heterogeneity test showed Q = 452.340, *p* < 0.001. Using a random-effects model, the combined effect size was 2.818, with a 95% CI of (1.641, 4.838). The change in the estimated combined effect size was not statistically significant, indicating that the impact of publication bias is minimal and the results are relatively robust.

### 3.7 Quality classification by GRADE

We evaluated the outcome measures of the 25 included studies. Prior to assessing the evidence quality using GRADEprofiler software, the default quality level of the evidence was designated as high. During the assessment process, the primary focus was on the downgrading of evidence quality. Factors that might lead to the downgrading of evidence quality included risk of bias, inconsistency, indirectness, imprecision, and publication bias. Based on the results of the quality classification test for grading, most of the outcome measures indicated low-quality studies (Supplementary Table 3).

## 4 Discussion

PLC is one of the most common types of gastrointestinal tumors and is considered a major risk factor threatening human health, ranking as the third leading cause of cancer mortality worldwide (Bray et al., 2024). Its pathogenesis is complex, involving multiple factors such as viral infection, liver cirrhosis, and metabolic abnormalities. Due to the lack of obvious symptoms in the early stages, patients are often diagnosed at advanced stages, missing the opportunity for surgical cure and resulting in poor overall prognosis (Liu T. et al., 2024). Traditional treatment methods, such as surgical resection, local ablation, chemotherapy, and molecular targeted therapies, have limited efficacy; moreover, some face issues of resistance, while others cause side effects (Wang et al., 2024). In recent years, the rise of immunotherapy, especially immune checkpoint inhibitors (ICIs), has brought new hope for liver cancer treatment (Llovet et al., 2024). Clinical studies have shown that ICI treatment can prolong the survival period of patients with few adverse reactions, making it a feasible adjuvant treatment on par with surgery, radiotherapy and chemotherapy (Wang J. K. et al., 2021). ICI is a monoclonal antibody that can block the interaction between checkpoint proteins and their ligands, thereby preventing T cell inactivation (Sangro et al., 2021). Among them, CTLA-4 inhibition and PD-1/PD-L1 blocking are the most commonly used checkpoint blocking strategies. CTLA-4 mainly regulates T cell activation by binding to the competing ligand of CD28 in the early initiation stage and inhibits DCs activity through Treg (Buchbinder and Desai, 2016). PD-1 inhibitors exert their effects in the later stage of the immune response by regulating T cell activity in peripheral tissues through interactions with PD-L1 and PD-L2 and promoting Treg differentiation (Rotte, 2019). Monotherapy with ICI has made progress in cancer treatment, but it also has certain limitations. For instance, the ORR is less than 20%, the PFS in most studies is only 2–4 months, some patients develop drug resistance, some trials fail to reach the primary endpoint, and it may also cause immune-related adverse events. Such as fatigue, rashes, gastrointestinal reactions and autoimmune thyroid diseases (Tong et al., 2025). Although ICIs monotherapy for HCC has been proven to have certain efficacy, the immunosuppressive nature of the tumor microenvironment and the development of drug resistance mechanisms often limit the persistence of anti-tumor responses. Therefore, the combined treatment strategy has become a more promising approach.

In recent years, traditional Chinese medicine has made significant progress in treating liver cancer, especially for patients in advanced stages. Multiple clinical randomized controlled trials have shown that traditional Chinese medicine combined with surgery, TACE, radiotherapy, or chemotherapy can improve patients’ immune systems, enhance their response rates to chemotherapy and radiotherapy, and alleviate side effects and complications caused by these treatments, thereby improving patients’ quality of life and survival rates (Yang et al., 2022). CKI, as a typical representative of botanical drug in China, has been approved by the National Medical Products Administration for the clinical treatment of pain or bleeding caused by tumors (Li S. L. et al., 2025). The clinical application of CKI is becoming increasingly widespread, and reports of its adverse drug reactions are also increasing accordingly. The adverse reactions of CKI are mainly gastrointestinal reactions (nausea, vomiting, abdominal distension, abdominal pain) and systemic damage (chills, chest tightness, flushing), and may also involve other systems, The occurrence of adverse reactions may be related to factors such as daily dosage, solvents, and combination therapy, The period within 30 min of the first infusion of CKI is the risk period for adverse reactions (Dian et al., 2020). CKI mainly exerts its therapeutic effects on liver cancer by directly targeting tumor cells; it also prevents the progression of precancerous liver lesions, regulates the tumor immune microenvironment, and modulates tumor cell metabolic reprogramming as well as the epithelial-mesenchymal transition process. Studies have shown that CKI targets the Smad7 protein in hepatic stellate cells (HSCs), promoting the degradation of TGFβR1 on the surface of HSCs, reducing the phosphorylation of downstream Smad2 and Smad3 signaling proteins, inhibiting HSC activation, and decreasing collagen structural protein accumulation in the liver, thereby inhibiting the progression from liver fibrosis to PLC (Yang et al., 2021). Some studies have found that CKI targets the TNFR1 receptor on tumor-associated macrophages, promoting the formation of a complex between TNFR1, TRADD, RIP1, and TRAF2. This activates downstream NF-κB p65 and p38 MAPK signaling axes, promotes polarization of tumor-associated macrophages toward the pro-inflammatory M1 phenotype, and enhances the proliferation and tumor-killing ability of CD8^+^ T cells, thereby exerting anti-liver cancer effects. Meanwhile, studies have also found that CKI can sensitize the treatment effect of low-dose sorafenib for liver cancer, and it has been proposed that combining botanical drugs with targeted chemotherapy drugs is a potential strategy for the clinical treatment of liver cancer (Yang et al., 2020). Compared with the camrelizumab plus chemotherapy group, the CKI plus camrelizumab plus chemotherapy group can improve the therapeutic effect of patients with advanced esophageal cancer, reduce the levels of serum tumor markers, improve immune function, and has good safety (Li X. T. et al., 2025). Studies have shown that compared with the PD-1 monoclonal antibody group, the combination of CKI and PD-1 monoclonal antibody has a better therapeutic effect in the treatment of advanced non-small cell lung cancer. It can improve the levels of serum CEA, NSE and Cyfra21-1 in patients, reduce the damage to liver, kidney and heart functions, and lower the incidence of adverse reactions and tumor recurrence and metastasis (Liao and Mao, 2024). CKI inhibits the expression of β-catenin and cyclooxygenase-2 and upregulates the expression of glycogen synthase kinase-3β (GSK-3β), thereby inhibiting the Wnt/β-catenin pathway and c-Myc expression. This suppresses glycolysis, regulates metabolic reprogramming, and inhibits epithelial-mesenchymal transition (Wang K. X. et al., 2021). Given the key role of immune function in CKI treatment for PLC, we conducted a meta-analysis to evaluate the latest progress regarding the immune effects of CKI combined with CT on PLC, providing potential scientific evidence for CKI in the clinical treatment of PLC.

### 4.1 Summary of findings

This meta-analysis assessed the impact of CKI on immune function, efficacy, and safety in patients with PLC. The main findings include: (a) CKI combined with CT significantly improved immune function, as evidenced by increased levels of CD3^+^, CD4^+^, CD4^+^/CD8^+^ ratio, and NK cells. The lack of statistical significance in the CD8^+^ outcome indicator may be related to the dual immunosuppressive and immunoregulatory roles of CD8^+^ cells. (b) Compared to CT alone, CKI combined with CT showed superior ORR and DCR and was able to reduce AFP levels. (c) Compared to CT alone, CKI combined with CT significantly improved KPS scores. (d) Compared to CT alone, CKI combined with CT significantly increased the one-year survival rate. (e) CKI combined with CT alleviated adverse reactions caused by biomedicine treatment, including nausea and vomiting, hepatic dysfunction, myelosuppression, fever, and pain. Sensitivity analysis confirmed the robustness of these findings. Egger’s test and Begg’s test were conducted to assess potential publication bias. The results suggested potential publication bias in the primary outcome indicators. To address this, the trim-and-fill method was employed to evaluate the stability of the combined results. The analysis showed that the change in the estimated combined effect size was not significant, indicating that the impact of publication bias is minimal and the results are relatively robust. Overall, this analysis emphasizes the potential of CKI combined with CT to enhance immune function, improve clinical outcomes, increase KPS scores, raise the one-year survival rate, and reduce adverse reactions in patients with PLC.

### 4.2 Comparison with previous studies

A previous meta-analysis (Yang et al., 2022) assessed the clinical efficacy and side effects of combining CKI with TACE in the treatment of PLC. Early studies had limitations such as small sample sizes; use of only single treatment combinations; a primary focus on clinical outcome indicators; failure to consider immune function and treatment duration; lack of subgroup analysis; and absence of Egger’s and Begg’s tests for publication bias, which may affect the reliability of the results. Additionally, from a clinical perspective, many studies on CKI combined with biomedicine for PLC lacked rigorous experimental design, had inconsistent inclusion criteria for enrolled patients, varied clinical observation indicators, and small sample sizes. These issues have contributed to insufficient evidence supporting the effectiveness of CKI combined with conventional treatment regimens for PLC. Therefore, to address these gaps, this study updates the meta-analysis of clinical randomized controlled trials investigating the immune regulatory effects of CKI combined with biomedicine for PLC. We screened eligible clinical trials, focused on key factors of PLC progression and treatment response, and specifically examined immune indicators such as CD3^+^, CD4^+^, CD8^+^, CD4^+^/CD8^+^ ratio, and NK cells. The study also employs subgroup analysis to explore the impact of different CKI treatment cycles on immune regulation, overall efficacy, and side effects in PLC. By applying evidence-based medicine methods for unified and standardized analysis of related clinical studies, this research provides more reliable and persuasive clinical evidence in this field.

### 4.3 Mechanism study of CKI on immune regulation

Mechanistically, CKI regulates immune function through multiple pathways. Compared to chemotherapy alone, CKI combined with cisplatin or paclitaxel significantly increased the activation rate of CD8^+^ T lymphocytes in the spleen and tumors of mice, enhanced the proportion of tumor-infiltrating CD8^+^ T cells, inhibited tumor-promoting signaling pathways such as specify pathways if known, and promoted T cell activation and immune responses. These effects suggest that CKI enhances the anti-tumor immune response in mice, thereby improving the efficacy of chemotherapy against triple-negative breast cancer (Liu et al., 2022). Furthermore, research indicates that CKI can downregulate Myc expression, reduce PD-L1 levels, and stimulate CD4^+^ T cells to release TNF-α, collectively contributing to its tumor-fighting activity (Li et al., 2023). CKI can activate T cell receptor pathways, induce NK cell activation, stimulate the proliferation and differentiation of Th1, Th2, and Th17 cells, and promote cytokine secretion, improving the prognosis of patients with triple-negative breast cancer (Liu et al., 2021). One of the main active metabolites of CKI, oxymatrine, can directly inhibit the proliferation of HepG2 and Hepa1-6 cells, induce apoptosis of HepG2 and Hepa1-6 cells, and simultaneously inhibit their migration. Oxymatrine enhances the immune therapeutic effect of the LAG-3 antibody *in vivo* and *in vitro* by downregulating the IL-6-mediated JAK2/STAT3 signaling pathway and reducing FGL1 expression (Chau et al., 2017). Oxymatrine can promote NK-92MI cells to secrete TNF-α and IFN-γ, inducing apoptosis in the human breast cancer cell line MCF-7 (Chen et al., 2019). Matrine can promote DC secretion of TNF-α, IL-6, and IL-12, reduce IL-10 secretion, enhance the expression of dendritic surface MHC-II, CD80, CD54, and CD86, thereby promoting DC maturation. Moreover, matrine activates TLR7/8 in dendritic cells, which leads to the increased expression of downstream signaling molecules MyD88, TNF receptor-associated factor 6, and IκB kinase, enhancing the tumor-killing effect of effector T cells (Wang J. K. et al., 2021). Taken together, these studies demonstrate that CKI and its main metabolites regulate immune function and inhibit tumors via multiple pathways.

### 4.4 Advantages and limitations

The progression and recurrence of PLC are closely related to immune dysfunction, with cellular immunity being the main immune regulatory mechanism against tumors. CD3^+^, CD4^+^, CD8^+^, and NK cells are important immune cells in the body. The CD3 complex of T cells consists of three different subunits that collectively promote the signal transduction required for T cell activation, providing the “first signal” to initiate T cell activation and determining the specificity of the immune response. It serves as the first immune barrier in the human immune mechanism and plays an important role in the immune function of PLC patients. Effector CD4^+^ T cells include Th1, Th2, and Th17 cells, which are the main force of cellular immunity. Th1 cells exert anti-tumor effects by secreting IFN-γ, TNF-α, and IL-2; Th2 cells exert anti-tumor or pro-tumor activity depending on different tumor types and disease states; and Th17 cells secrete IL-17 to promote tumor growth (Sun et al., 2023). CD8^+^ T cells influence the immune function of PLC patients in a bidirectional regulatory manner. On one hand, CD8^+^ T cells have the ability to selectively detect and eradicate cancer cells (Jäger et al., 2000); on the other hand, CD8^+^ T cells can secrete inhibitory T lymphocyte factors, exerting specific immune suppression (Khazaie and von Boehmer, 2006). The CD4^+^/CD8^+^ ratio is an important indicator reflecting the body’s immune regulation, with a decrease in this ratio indicating that the body’s cellular immune function is in a suppressed state (Li S. L. et al., 2025). NK cells are a subset of innate lymphocytes and are innate immune effector cells with a unique ability to distinguish between normal and transformed cells. When NK cells recognize abnormal cells (such as tumor cells), they are rapidly activated and eliminate tumor cells by secreting granzyme, perforin, and through death receptor-mediated pathways (Malmberg et al., 2017). This meta-analysis focuses on these immune indicators, providing a new perspective on the role of CKI in regulating immune function in PLC patients. To improve the reliability of the findings, we conducted subgroup analyses based on different CKI treatment cycles to exclude potential confounding factors related to treatment duration. Furthermore, this study explored publication bias through Egger’s and Begg’s tests to assess the reliability of the results.

This study has evidence-based significance. However, it has the following shortcomings: (1) In terms of literature sources, although extensive literature searches were conducted, the studies included in the meta-analysis were all clinical randomized controlled trials conducted domestically, and the results were published in Chinese journals, which may introduce potential language and publication biases; (2) Regarding literature quality, most studies lack detailed and specific descriptions of randomization methods, blinding, allocation concealment, etc., which could increase the risk of selection bias, implementation bias, and expectation bias; (3) In terms of CKI treatment dosage, duration, and quality control standards, there are differences in dosage and treatment cycles of CKI among the included studies, and quality control standards were either not reported or inconsistent, which may lead to heterogeneity in results and affect evaluation outcomes; (4) Regarding combination treatment regimens, the drug combinations used in various studies are inconsistent, and the interaction mechanisms between CKI and different treatment regimens remain unclear, which may increase heterogeneity in results and impact conclusions; (5) In terms of follow-up, long-term follow-up data were not comprehensively reported, with missing overall survival (OS) or progression-free survival (PFS) data, leaving the prognostic value of CKI combined treatment unclear; (6) Regarding disease staging, most included studies did not report disease stage, preventing stratified analysis by stage, which may contribute to heterogeneity and affect conclusions; (7) In terms of sample size, the patient cohorts in the included studies were small and lacked multi-center, large-sample randomized controlled trials, which may impact the robustness of evaluation outcomes.

### 4.5 Implication

To further strengthen the evidence for CKI in the treatment of PLC, future clinical research needs to focus on targeted optimization around existing shortcomings, particularly in the following key areas: (1) To expand the scope of research inclusion, breakthroughs should be achieved in overcoming regional and language barriers; multi-center clinical RCTs should be included, and international communications and cooperations should be strengthened. (2) Research design specifications should be strictly followed, with detailed explanations of randomization methods (such as detailed implementation procedures for stratified randomization), types of blinding (such as the subjects of single-blind/double-blind and unblinding criteria), and allocation concealment strategies (such as the sealed-envelope method) in the research protocol to reduce the risk of selection bias, implementation bias, and expectation bias from the source. (3) The optimal dosage (such as standardized doses calculated according to body surface area) and treatment cycles should be clarified in conjunction with pharmacokinetic and pharmacodynamic analyses. Moreover, a unified quality control system should be established to reduce heterogeneity in results caused by differences in intervention regimens. (4) Different combination regimens should be designed based on the pathogenesis of primary liver cancer (such as angiogenesis and immunosuppressive microenvironment); for example, CKI combined with interventional therapy, targeted drugs, or immune checkpoint inhibitors. In-depth analyses of mechanisms should be conducted through basic experiments and clinical sample analyses (such as tumor tissue immunohistochemical analysis and single-cell sequencing of peripheral blood samples) to enhance the reliability and reproducibility of conclusions. (5) Long-term follow-up periods should be established, with regular imaging assessments, tumor marker tests, etc., systematically collecting core prognostic indicators such as overall survival (OS) and progression-free survival (PFS), and should include quality of life assessments and long-term safety data to clarify the long-term benefits and risks of CKI combined treatment. (6) Patient tumor staging should be considered in research design, with detailed records of tumor size, lymph node metastasis, distant metastasis, and liver function grading; based on these, stratified subgroup analyses should be conducted to avoid heterogeneity in results caused by confounding factors related to staging and clarify the benefit differences among patients with different conditions. (7) High-level clinical trials with large sample sizes and multiple centers should be designed to exclude the interference of insufficient sample sizes on results. (8) Multi-dimensional detection technologies should be used to analyze the subgroup characteristics of CD8^+^ T cells, together with tumor microenvironment analyses, to clarify their dual roles in immune suppression and immune regulation at the molecular level. (9) Subsequent studies need to explore the specific mechanisms by which CKI regulates immune responses, such as distinguishing its effects on circulating immune cells and tumor-infiltrating lymphocytes. Moreover, in addition to studying T lymphocytes and NK cells, research should also include dendritic cells, macrophages, and tumor-associated macrophages. Although this meta-analysis focuses on PLC, literature analysis suggests that CKI may have potential benefits for other malignancies characterized by immune dysfunction. Therefore, future research should explore the role of CKI in various cancers such as lung cancer and gastric cancer, potentially expanding its clinical applications. Through the aforementioned multi-dimensional research optimization, it is possible to comprehensively address the existing evidence gaps, thereby further solidifying the evidence-based foundation for CKI in the treatment of primary liver cancer and providing more reliable scientific evidence for clinical decision-making.

## 5 Conclusion

This meta-analysis found that CKI combined with CT for PLC shows better immunomodulatory effects and clinical outcomes than CT alone. CKI combined with CT effectively improves patients’ immune function, increases ORR and DCR, reduces AFP levels, and improves patients’ KPS scores and one-year survival rate. In addition, CKI combined with CT alleviates gastrointestinal reactions, fever, and other side effects caused by CT. However, these conclusions should be interpreted with caution, as the varying quality of studies, lack of disease staging stratified analysis, short observation periods, and absence of long-term follow-up data indicate that high-quality, large-scale randomized controlled trials are needed to thoroughly evaluate its immunomodulatory effects, long-term efficacy, safety, and clinical applicability in PLC treatment.

## Data Availability

The raw data supporting the conclusions of this article will be made available by the authors, without undue reservation.

## References

[B1] Ba Y. H. (2018). Clinical observation on transcatheter arterial chemoembolization combined with compound kushen injection in the treatment of 42 cases of primary liver cancer. Chin. J. Ethnomed Ethnopharm 27 (20), 82–84. 10.3969/j.issn.1007-8517.2018.20.zgmzmjyyzz201820029

[B2] Bray F. Laversanne M. Sung H. Ferlay J. Siegel R. L. Soerjomataram I. (2024). Global cancer statistics 2022: GLOBOCAN estimates of incidence and mortality worldwide for 36 cancers in 185 countries. CA A Cancer J. Clin. 74 (3), 229–263. 10.3322/caac.21834 38572751

[B3] Buchbinder E. I. Desai A. (2016). CTLA-4 and PD-1 pathways: similarities, differences, and implications of their inhibition. Am. J. Clin. Oncol. 39 (1), 98–106. 10.1097/coc.0000000000000239 26558876 PMC4892769

[B4] Cao J. Liu H. Q. He Y. Xia N. Zhang H. L. (2011). Clinical analysis of TACE combined with compound Kushen injection in the treatment of primary liver cancer. Hebei Med. J. 33 (18), 2783–2784. 10.3969/j.issn.1002-7386.2011.18.033

[B5] Chan Y. T. Zhang C. Wu J. Lu P. Xu L. Yuan H. (2024). Biomarkers for diagnosis and therapeutic options in hepatocellular carcinoma. Mol. Cancer 23 (1), 189. 10.1186/s12943-024-02101-z 39242496 PMC11378508

[B6] Chau I. Peck-Radosavljevic M. Borg C. Malfertheiner P. Seitz J. F. Park J. O. (2017). Ramucirumab as second-line treatment in patients with advanced hepatocellular carcinoma following first-line therapy with sorafenib: patient-focused outcome results from the randomised phase III REACH study. Eur. J. Cancer 81, 17–25. 10.1016/j.ejca.2017.05.001 28591675

[B7] Chen G. H. Li S. R. Yang L. (2007). Efficacy observation of compound Kushen injection as adjuvant therapy in double interventional treatment of primary liver cancer. Chin. J. Integr. Tradit. West Med. Dig. (04), 239–241. 10.3969/j.issn.1671-038X.2007.04.010

[B8] Chen D. L. Wang Q. Li Z. (2019). Oxymatrine enhances the sensitivity of breast cancer MCF-7 cells to the cytotoxicity of NK-92MI cells. Chin. J. Immunol. 35 (06), 680–685. 10.3969/j.issn.1000-484X.2019.06.008

[B9] Cumpston M. Li T. Page M. J. Chandler J. Welch V. A. Higgins J. P. (2019). Updated guidance for trusted systematic reviews: a new edition of the cochrane handbook for systematic reviews of interventions. Cochrane Database Syst. Rev. 10 (10), Ed000142. 10.1002/14651858.Ed000142 31643080 PMC10284251

[B10] Dian L. P. Wei X. Q. Tang F. Zhang Y. Z. Zhong S. W. (2020). Analysis of adverse reactions and safe medication of compound Kushen injection based on literature reports. China Med. Her. 17 (35), 140–143. 10.20047/j.issn1673-7210.2020.35.036

[B11] Dong W. H. Hu L. R. Chen S. Chen J. B. Li H. J. Chen H. S. (2016). Clinical study on Compound Kushen injection combined with transcatheter arterial chemoembolization in the treatment of primary liver cancer. Liaoning J. Tradit. Chin. Med. 43 (12), 2564–2565. 10.13192/j.issn.1000-1719.2016.12.033

[B12] Du S. S. Chen G. W. Yang P. Chen Y. X. Hu Y. Zhao Q. Q. (2022). Radiation therapy promotes hepatocellular carcinoma immune cloaking via PD-L1 upregulation induced by cGAS-STING activation. Int. J. Radiat. Oncol. Biol. Phys. 112 (5), 1243–1255. 10.1016/j.ijrobp.2021.12.162 34986380

[B13] Guan C. N. Cai L. Z. Yue L. Q. Zhang Y. (2006). Clinical study on Yanshu Injection combined with chemotherapy in the treatment of advanced primary liver cancer. China J. Chin. Mater Med. 31 (06), 510–512. 10.3321/j.issn:1001-5302.2006.06.022 16722388

[B14] Guo G. J. (2015). Effect observation of compound Kushen injection as adjuvant therapy in transcatheter arterial chemoembolization for primary liver cancer. J. Massage Rehabil. Med. 6 (21), 51–52.

[B15] Han W. L. Yuan C. Y. Zhang T. S. (2012). Efficacy observation of compound Kushen injection combined with interventional chemotherapy in the treatment of primary liver cancer and its influence on immune function. Chin. J. Tradit. Med. Sci. Technol. 19 (01), 61–62. 10.3969/j.issn.1005-7072.2012.01.04

[B16] Hao C. H. Tian L. B. He J. (2020). Preventive effect of compound Kushen injection on postoperative recurrence of primary liver cancer and analysis of survival function. Chin. J. Integr. Tradit. West Med. Liver Dis. 30 (04), 303–306. 10.3969/j.issn.1005-0264.2020.04.005

[B17] He J. Zhang D. W. Zhang H. G. Li L. Wang L. L. Ma L. B. (2016). Effect of Compound Kushen Injection on immune function of patients with primary liver cancer after hepatectomy. Chin. Tradit. Pat. Med. 38 (08), 1700–1702. 10.3969/j.issn.1001-1528.2016.08.008

[B18] Hou C. L. Hu Y. N. (2019). Effect of Compound Kushen Injection combined with apatinib on clinical efficacy, quality of life and safety in patients with advanced non-small cell lung cancer. J. Pract. Oncol. 34 (07), 1176–1178. 10.3969/j.issn.1001-5930.2019.07.037

[B19] Hwang S. Y. Danpanichkul P. Agopian V. Mehta N. Parikh N. D. Abou-Alfa G. K. (2025). Hepatocellular carcinoma: updates on epidemiology, surveillance, diagnosis and treatment. Clin. Mol. Hepatol. 31 (Suppl. l), S228–S254. 10.3350/cmh.2024.0824 39722614 PMC11925437

[B20] Jäger E. Nagata Y. Gnjatic S. Wada H. Stockert E. Karbach J. (2000). Monitoring CD8 T cell responses to NY-ESO-1: correlation of humoral and cellular immune responses. Proc. Natl. Acad. Sci. U. S. A. 97 (9), 4760–4765. 10.1073/pnas.97.9.4760 10781081 PMC18306

[B21] Jiang X. Gao G. Y. Wang W. Zhou X. Y. Gu R. H. Shi H. C. (2017). Clinical study on Compound Kushen Injection combined with radioactive 125I seed implantation in the treatment of primary liver cancer. Guizhou Med. J. 41 (01), 39–41. 10.3969/j.issn.1000-744X.2017.01.015

[B22] Khazaie K. von Boehmer H. (2006). The impact of CD4+CD25+ Treg on tumor specific CD8+ T cell cytotoxicity and cancer. Semin. Cancer Biol. 16 (2), 124–136. 10.1016/j.semcancer.2005.11.006 16443370

[B23] Li G. (2020). Clinical efficacy of Compound Kushen Injection combined with TACE in the treatment of primary liver cancer. Pract. Clin. J. Integr. Tradit. West Med. 20 (03), 45–46+85. 10.13638/j.issn.1671-4040.2020.03.023

[B24] Li Z. J. Wang Y. (2021). Effect of Compound Kushen Injection on hepatocellular carcinoma after transcatheter arterial chemoembolization combined with radiofrequency ablation. Chin. J. Integr. Tradit. West Med. Imaging 19 (01), 31–33. 10.3969/j.issn.1672-0512.2021.01.008

[B25] Li X. He L. Ou Y. Wang S. Hu Y. Niu H. (2023). Oxymatrine inhibits melanoma development by modulating the immune microenvironment and targeting the MYC/PD-L1 pathway. Int. Immunopharmacol. 124 (Pt B), 111000. 10.1016/j.intimp.2023.111000 37788594

[B26] Li S. L. S. L. Liu L. M. Yin X. Zhan H. Li Y. Z. Yu Y. H. (2025). Effect of Compound Kushen Injection combined with tislelizumab on short-term efficacy, lung function and immune function in patients with non-small cell lung cancer undergoing chemotherapy. Drug Eval. Res. 48 (04), 978–983. 10.7501/j.issn.1674-6376.2025.04.020

[B27] Li X. T. Li F. Fu J. Y. (2025). Effect of Compound Kushen injection combined with camrelizumab and chemotherapy on efficacy and immune function in patients with advanced esophageal cancer. J. Aerosp. Med. 36 (10), 1221–1223.

[B28] Liao S. H. Mao Y. (2024). Efficacy of Compound Kushen injection combined with PD-1 inhibitor in the treatment of locally advanced and advanced non-small cell lung cancer and its influence on serum CEA. Jiangxi Med. J. 59 (12), 1146–1150. 10.3969/j.issn.1006-2238.2024.12.014

[B29] Liu X. Wu Y. Zhang Y. Bu D. Wu C. Lu S. (2021). High throughput transcriptome data analysis and computational verification reveal immunotherapy biomarkers of Compound Kushen injection for treating triple-negative breast cancer. Front. Oncol. 11, 747300. 10.3389/fonc.2021.747300 34604090 PMC8484800

[B30] Liu X. Bai M. Li H. Ye P. Duan X. Wu C. (2022). Single-cell RNA-sequencing uncovers compound kushen injection synergistically improves the efficacy of chemotherapy by modulating the tumor environment of breast cancer. Front. Immunol. 13, 965342. 10.3389/fimmu.2022.965342 36389835 PMC9660330

[B31] Liu Z. Zhu Y. Xie H. Zou Z. (2022). Immune-mediated hepatitis induced by immune checkpoint inhibitors: current updates and future perspectives. Front. Pharmacol. 13, 1077468. 10.3389/fphar.2022.1077468 36699050 PMC9868416

[B32] Liu L. Q. Dong D. H. Chen Y. N. (2024). Clinical efficacy of Compound Kushen injection combined with FOLFOX4 chemotherapy regimen in the treatment of advanced liver cancer. Shenzhen J. Integr. Tradit. West Med. 34 (08), 57–60. 10.16458/j.cnki.1007-0893.2024.08.016

[B33] Liu T. Meng G. Ma S. You J. Yu L. He R. (2024). Progress of immune checkpoint inhibitors in the treatment of advanced hepatocellular carcinoma. Front. Immunol. 15, 1455716. 10.3389/fimmu.2024.1455716 39185414 PMC11341420

[B34] Llovet J. M. Pinyol R. Yarchoan M. Singal A. G. Marron T. U. Schwartz M. (2024). Adjuvant and neoadjuvant immunotherapies in hepatocellular carcinoma. Nat. Rev. Clin. Oncol. 21 (4), 294–311. 10.1038/s41571-024-00868-0 38424197 PMC11984461

[B35] Lu J. Wang W. D. Fan C. Xu P. Wang W. T. Ding Y. (2011). Clinical observation on Compound Kushen injection as adjuvant therapy in transcatheter arterial chemoembolization for primary liver cancer. Mod. J. Integr. Tradit. West Med. 20 (36), 4661–4662. 10.3969/j.issn.1008-8849.2011.36.036

[B37] Lunny C. Thirugnanasampanthar S. S. Kanji S. Ferri N. Pieper D. Whitelaw S. (2022). How can clinicians choose between conflicting and discordant systematic reviews? A replication study of the Jadad algorithm. BMC Med. Res. Methodol. 22 (1), 276. 10.1186/s12874-022-01750-2 36289496 PMC9597955

[B36] Lv X. Y. Dai B. Liu C. (2023). Effect of Compound Kushen injection on chemotherapy efficacy and T-cell immunity in patients with hepatitis B-related hepatocellular carcinoma. Chin. J. Ration. Drug Use 20 (10), 61–66. 10.3969/j.issn.2096-3327.2023.10.010

[B38] Malmberg K. J. Carlsten M. Björklund A. Sohlberg E. Bryceson Y. T. Ljunggren H. G. (2017). Natural killer cell-mediated immunosurveillance of human cancer. Semin. Immunol. 31, 20–29. 10.1016/j.smim.2017.08.002 28888619

[B39] Ning J. Wang Y. Tao Z. (2024). The complex role of immune cells in antigen presentation and regulation of T-cell responses in hepatocellular carcinoma: progress, challenges, and future directions. Front. Immunol. 15, 1483834. 10.3389/fimmu.2024.1483834 39502703 PMC11534672

[B40] Powell N. Ibraheim H. Raine T. Speight R. A. Papa S. Brain O. (2020). British Society of Gastroenterology endorsed guidance for the management of immune checkpoint inhibitor-induced enterocolitis. Lancet Gastroenterol. Hepatol. 5 (7), 679–697. 10.1016/s2468-1253(20)30014-5 32553146

[B41] Ren Z. Q. Feng J. D. (2024). Observation on clinical efficacy, immune function and adverse reactions of Compound Kushen injection as adjuvant therapy in transcatheter arterial chemoembolization for primary liver cancer. Liaoning J. Tradit. Chin. Med. 51 (04), 125–128. 10.13192/j.issn.1000-1719.2024.04.034

[B42] Rotte A. (2019). Combination of CTLA-4 and PD-1 blockers for treatment of cancer. J. Exp. Clin. Cancer Res. 38 (1), 255. 10.1186/s13046-019-1259-z 31196207 PMC6567914

[B43] Sangro B. Sarobe P. Hervás-Stubbs S. Melero I. (2021). Advances in immunotherapy for hepatocellular carcinoma. Nat. Rev. Gastroenterol. Hepatol. 18 (8), 525–543. 10.1038/s41575-021-00438-0 33850328 PMC8042636

[B44] Sun L. Su Y. Jiao A. Wang X. Zhang B. (2023). T cells in health and disease. Signal Transduct. Target Ther. 8 (1), 235. 10.1038/s41392-023-01471-y 37332039 PMC10277291

[B45] Teng M. W. Ngiow S. F. Ribas A. Smyth M. J. (2015). Classifying cancers based on T-cell infiltration and PD-L1. Cancer Res. 75 (11), 2139–2145. 10.1158/0008-5472.Can-15-0255 25977340 PMC4452411

[B46] Tong J. Tan Y. Ouyang W. Chang H. (2025). Targeting immune checkpoints in hepatocellular carcinoma therapy: toward combination strategies with curative potential. Exp. Hematol. Oncol. 14 (1), 65. 10.1186/s40164-025-00636-5 40317077 PMC12046748

[B47] Wang Z. F. (2009). Efficacy observation of Compound Kushen injection via hepatic arterial perfusion in the treatment of liver cancer. Shandong Med. J. 49 (08), 102. 10.3969/j.issn.1002-266X.2009.08.058

[B48] Wang D. Xu Y. Huang T. Peng W. Zhu D. Zhou X. (2022). Clinical efficacy and safety of NSCLC ancillary treatment with compound Kushen injection through immunocompetence regulation: a systematic review and meta-analysis. Phytomedicine 104, 154315. 10.1016/j.phymed.2022.154315 35868145

[B49] Wang X. Yang Y. Zhao S. Wu D. Li L. Zhao Z. (2024). Chitosan-based biomaterial delivery strategies for hepatocellular carcinoma. Front. Pharmacol. 15, 1446030. 10.3389/fphar.2024.1446030 39161903 PMC11330802

[B50] Wang R. Y. Zhang Y. Wang J. H. Zhang Z. X. (2025). Mechanism study of Compound Kushen injection in alleviating chemotherapeutic adverse reactions of 5-Fluorouracil in rats with hepatocellular carcinoma. Tianjin Med. J. 53 (01), 24–29.

[B52] Wang K. X. Du G. H. Qin X. M. Gao L. (2021). Compound Kushen injection intervenes metabolic reprogramming and epithelial-mesenchymal transition of HCC via regulating β-catenin/c-Myc signaling. Phytomedicine 93, 153781. 10.1016/j.phymed.2021.153781 34649212

[B54] Wu S. Liu S. Cao Y. Chao G. Wang P. Pan H. (2022). Downregulation of ZC3H13 by miR-362-3p/miR-425-5p is associated with a poor prognosis and adverse outcomes in hepatocellular carcinoma. Aging (Albany NY) 14 (5), 2304–2319. 10.18632/aging.203939 35278064 PMC8954979

[B55] Xu P. Wang W. D. Lu J. Fan C. (2012). Effect of Compound Kushen injection on T-lymphocyte subsets in patients with primary liver cancer treated with TACE. Mod. J. Integr. Tradit. West Med. 21 (12), 1313–1314. 10.3969/j.issn.1008-8849.2012.12.034

[B56] Yang J. D. Hainaut P. Gores G. J. Amadou A. Plymoth A. Roberts L. R. (2019). A global view of hepatocellular carcinoma: trends, risk, prevention and management. Nat. Rev. Gastroenterol. Hepatol. 16 (10), 589–604. 10.1038/s41575-019-0186-y 31439937 PMC6813818

[B57] Yang Y. Sun M. Yao W. Wang F. Li X. Wang W. (2020). Compound kushen injection relieves tumor-associated macrophage-mediated immunosuppression through TNFR1 and sensitizes hepatocellular carcinoma to sorafenib. J. Immunother. Cancer 8 (1). 10.1136/jitc-2019-000317 32179631 PMC7073790

[B58] Yang Y. Sun M. Li W. Liu C. Jiang Z. Gu P. (2021). Rebalancing TGF-β/Smad7 signaling via Compound kushen injection in hepatic stellate cells protects against liver fibrosis and hepatocarcinogenesis. Clin. Transl. Med. 11 (7), e410. 10.1002/ctm2.410 34323416 PMC8255064

[B59] Yang W. L. Xu H. M. Zhao H. L. Liu Q. Nie Y. Q. (2022). Meta-analysis of clinical efficacy and safety of Compound Kushen injection combined with transcatheter arterial chemoembolization in the treatment of primary liver cancer. Lishizhen Med. Mater Med. Res. 33 (03), 767–770. 10.3969/j.issn.1008-0805.2022.03.75

[B60] Yao H. R. Tian J. Li Y. C. Zhang W. Q. Hao Q. (2014). Study on the content and suppressive function of CD4+ CD25+ regulatory T cells in ascites and peripheral blood of patients with ovarian cancer. Chin. J. Clin. Oncol. 41 (12), 787–792. 10.3969/j.issn.1000-8179.20131281

[B61] Yao H. Du X. Q. Chen Z. (2021). Clinical efficacy of Compound Kushen injection combined with transcatheter arterial chemoembolization in the treatment of advanced liver cancer and its influence on immune regulation. J. Hubei Univ. Chin. Med. 23 (01), 22–25. 10.3969/j.issn.1008-987x.2021.01.05

[B62] You G. C. Gao Y. Zhang Q. (2018). Efficacy of Compound Kushen injection combined with interventional therapy in the treatment of primary liver cancer and its influence on liver function of patients. Shaanxi J. Tradit. Chin. Med. 39 (12), 1722–1724. 10.3969/j.issn.1000-7369.2018.12.019

[B63] Yuan X. Y. (2018). Evaluation of the effect of Compound Kushen injection as adjuvant therapy in transcatheter arterial chemoembolization for primary liver cancer. J. Hubei Minzu Univ. Med. Ed. 35 (04), 81–82+85. 10.13501/j.cnki.42-1590/r.2018.04.024

[B64] Yue X. D. (2024). Research progress on the mechanism of Compound Kushen injection against liver cancer cells. J. Front. Med. 14 (33), 52–54. 10.3969/j.issn.2095-1752.2024.33.013

[B65] Zhang X. F. Zhou M. (2014). Adjuvant chemotherapy with Compound Kushen injection in the treatment of 48 cases of primary liver cancer. Henan J. Tradit. Chin. Med. 34 (10), 1929–1930. 10.16367/j.issn.1003-5028.2014.10.127

[B66] Zhang D. W. He J. Zhang H. G. Hao C. H. Zhang Y. W. Wang L. L. (2017). Study on the effect of Compound Kushen injection combined with intraoperative margin management on postoperative recurrence and metastasis of primary liver cancer. Mod. J. Integr. Tradit. West Med. 26 (21), 2348–2350. 10.3969/j.issn.1008-8849.2017.21.024

[B67] Zhang D. W. He J. Zhang H. G. Wang L. L. Hao C. H. Zhang Y. W. (2018). Clinical effect of Compound Kushen injection in preventing recurrence and metastasis of primary liver cancer. World J. Tradit. Chin. Med. 13 (03), 601–604. 10.3969/j.issn.1673-7202.2018.03.013

[B68] Zhang A. Fan T. Liu Y. Yu G. Li C. Jiang Z. (2024). Regulatory T cells in immune checkpoint blockade antitumor therapy. Mol. Cancer 23 (1), 251. 10.1186/s12943-024-02156-y 39516941 PMC11545879

[B69] Zhao Y. X. Liu L. Lu L. Gao R. H. Sun Z. F. Wang Q. Y. (2018). Study on the effect of Compound Kushen injection on quality of life and therapeutic efficacy in ovarian cancer patients undergoing chemotherapy. Chin. J. Clin. 46 (01), 89–91. 10.3969/j.issn.2095-8552.2018.01.032

[B70] Zheng J. Wang S. Xia L. Sun Z. Chan K. M. Bernards R. (2025). Hepatocellular carcinoma: signaling pathways and therapeutic advances. Signal Transduct. Target Ther. 10 (1), 35. 10.1038/s41392-024-02075-w 39915447 PMC11802921

[B71] Zhou X. L. (2021). Effect of Compound Kushen injection combined with transcatheter arterial chemoembolization in the treatment of patients with primary liver cancer. Chin. J. People's Health 33 (10), 72–74. 10.3969/j.issn.1672-0369.2021.10.030

