# Supplementary material for: Effect of compound kushen injection on immune function in patients with primary liver cancer: a systematic review and meta-analysis
Source: Front Pharmacol. 2026 Feb 19;17:1715798. doi: 10.3389/fphar.2026.1715798 (PMC12960130; doi:10.3389/fphar.2026.1715798)
Supplement: Supplementary file 2 [file Supplementaryfile1.docx]

Figure S1

**Depression**

S1a


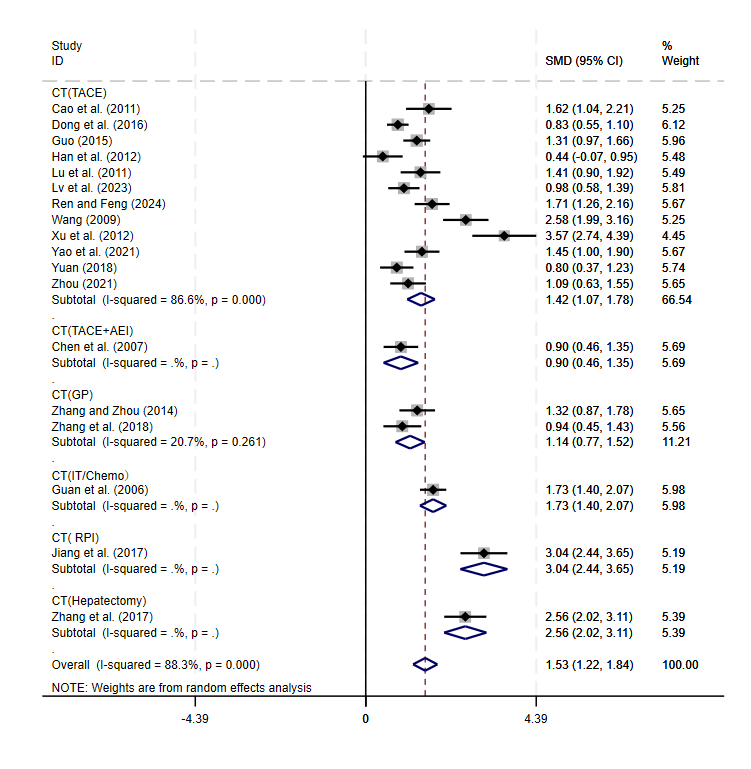


Fig. S1a. Forest plot for CD3^+^ levels of CKI combined with TACE, TACE+AEI, GP, IT/Chemo, RPI, or Hepatectomy in PLC treatment.

S1b


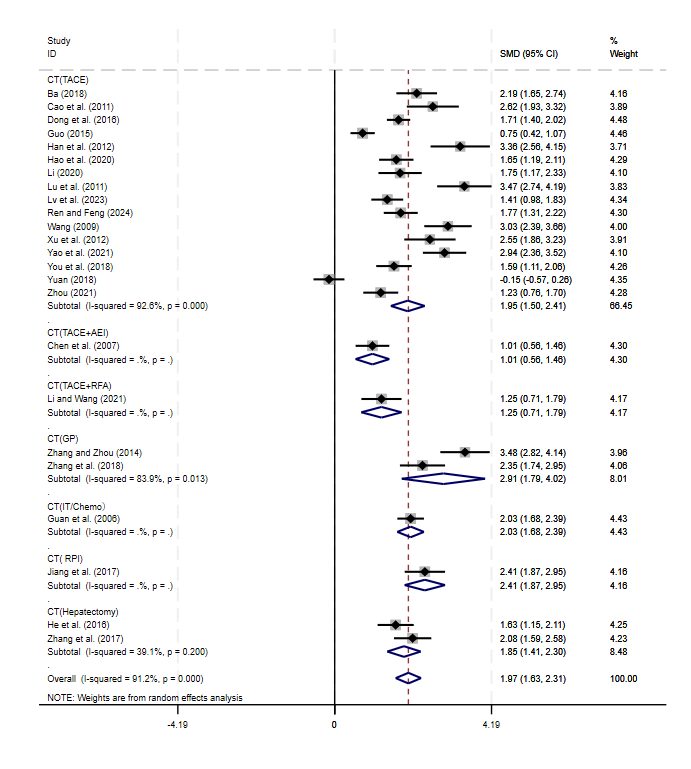


Fig. S1b. Forest plot for CD4^+^ levels of CKI combined with TACE, TACE+AEI, TACE+RFA, GP, IT/Chemo, RPI, or Hepatectomy in PLC treatment

S1c


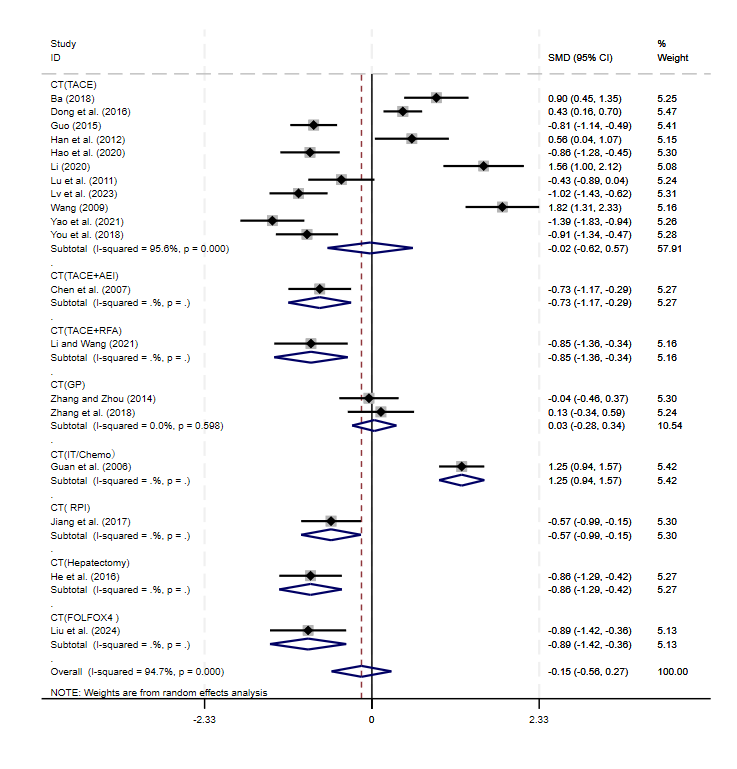


Fig. S1c. Forest plot for CD8^+^ levels of CKI combined with TACE, TACE+AEI, TACE+RFA, GP, IT/Chemo, RPI, Hepatectomy, or FOLFOX4 in PLC treatment

S1d


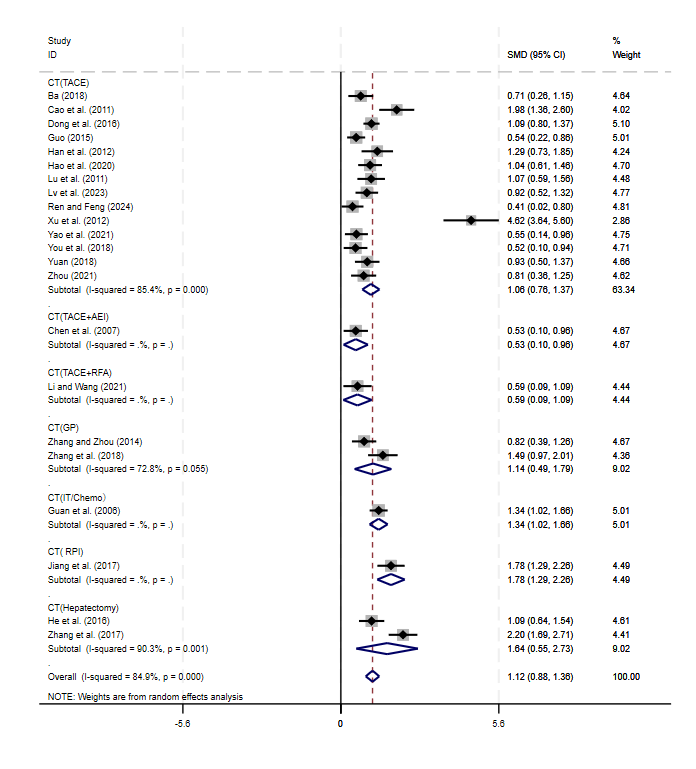


Fig. S1d. Forest plot for CD4^+^/CD8^+^ ratio of CKI combined with TACE, TACE+AEI, TACE+RFA, GP, IT/Chemo, RPI, or Hepatectomy in PLC treatment

S1e


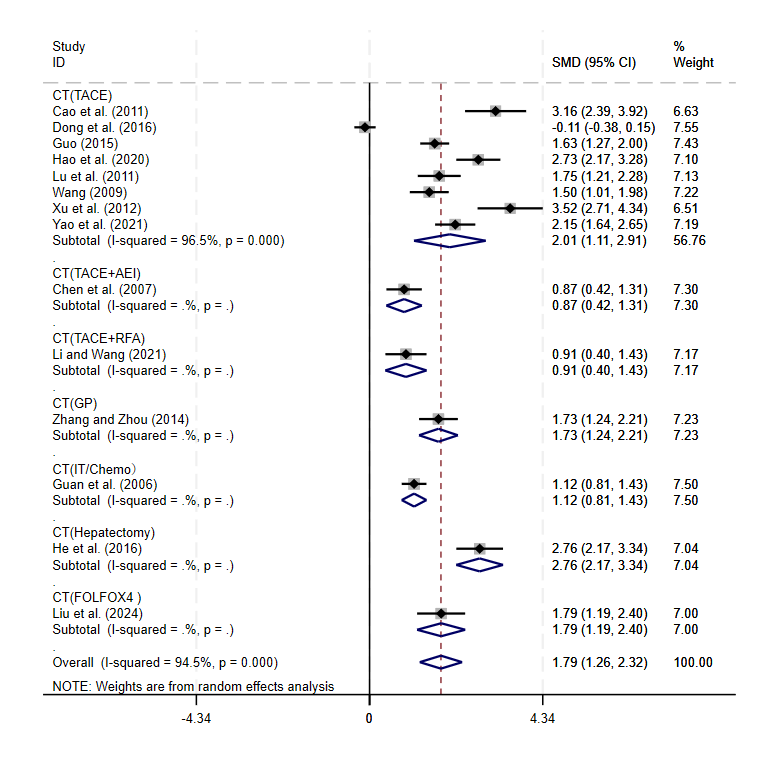


Fig. S1e. Forest plot for NK cell levels of CKI combined with TACE, TACE+AEI, TACE+RFA, GP, IT/Chemo, Hepatectomy, or FOLFOX4 in PLC treatment

S1f


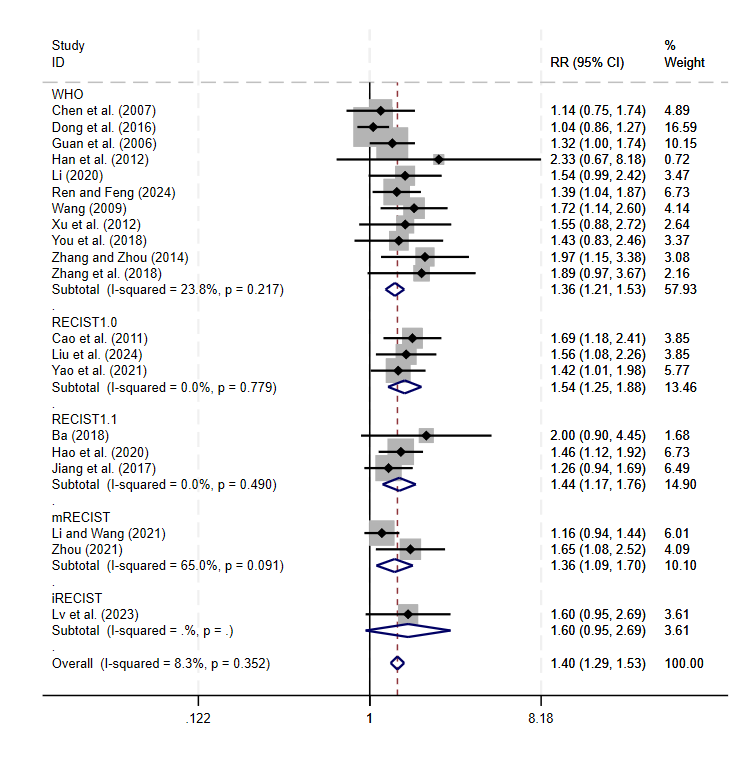


Fig. S1f. Forest plot for ORR of response evaluation criteria in Solid Tumors

S1g


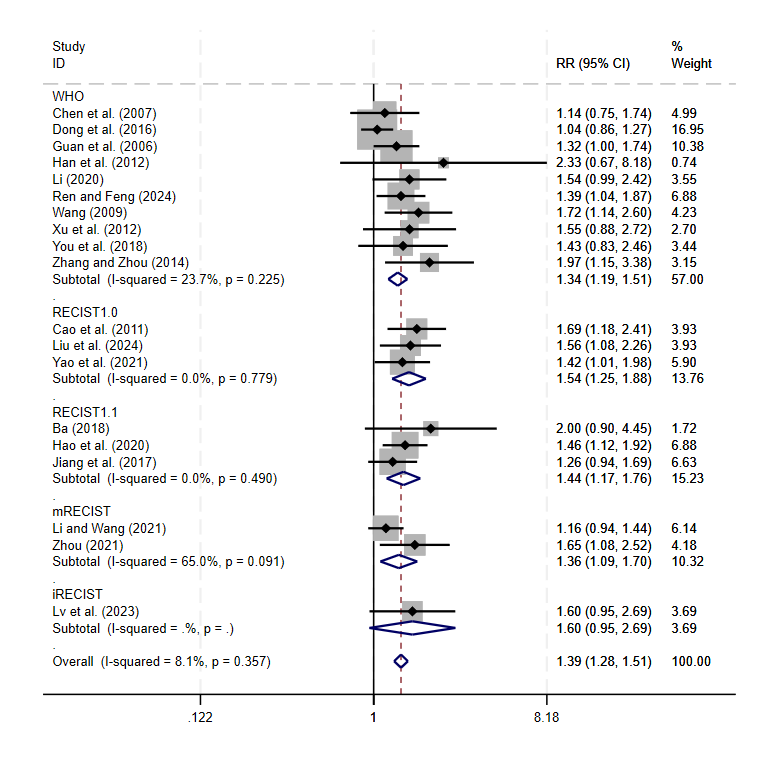


Fig. S1g. Forest plot for DCR of response evaluation criteria in Solid Tumors

S1h


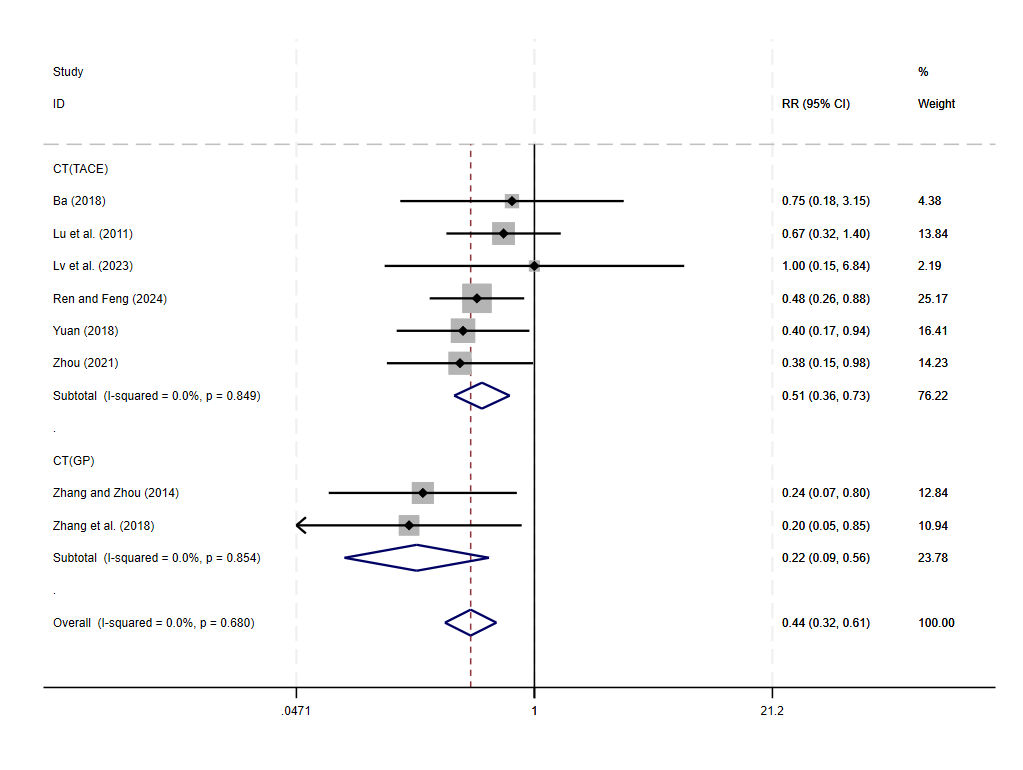


Fig. S1h. Forest plot for fever of CKI combined with TACE, GP in PLC treatment

S1i


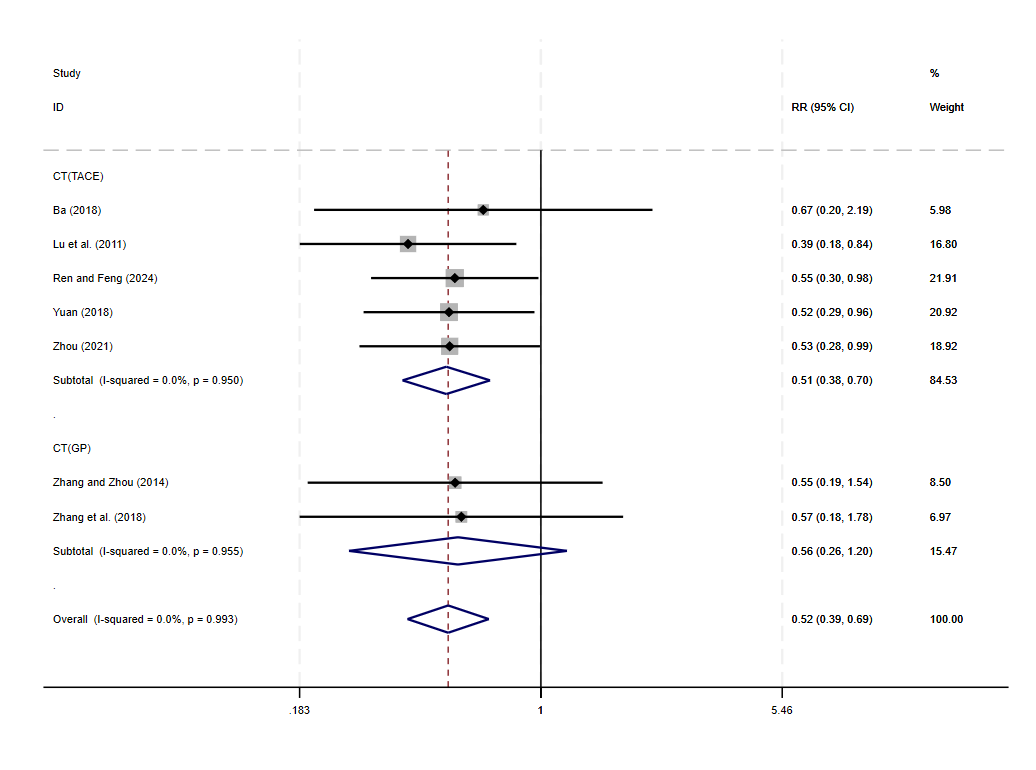


Fig. S1i. Forest plot for pain of CKI combined with TACE, GP in PLC treatment

S1j


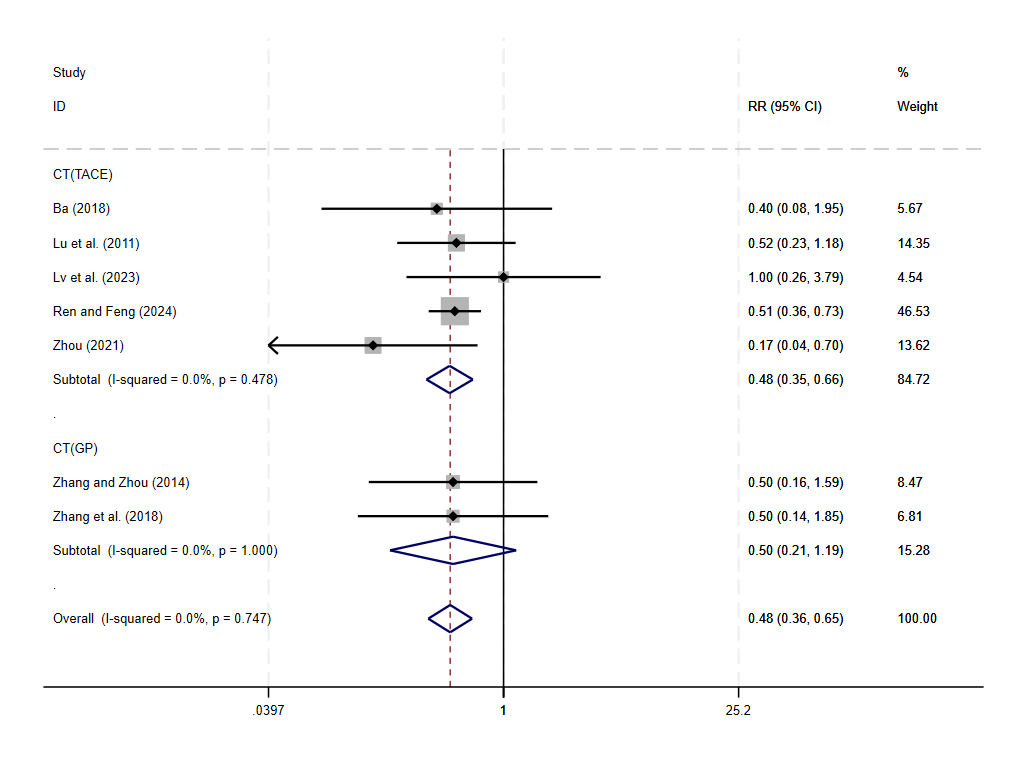


Fig. S1j. Forest plot for nausea and vomiting of CKI combined with TACE, GP in PLC treatment

S1k


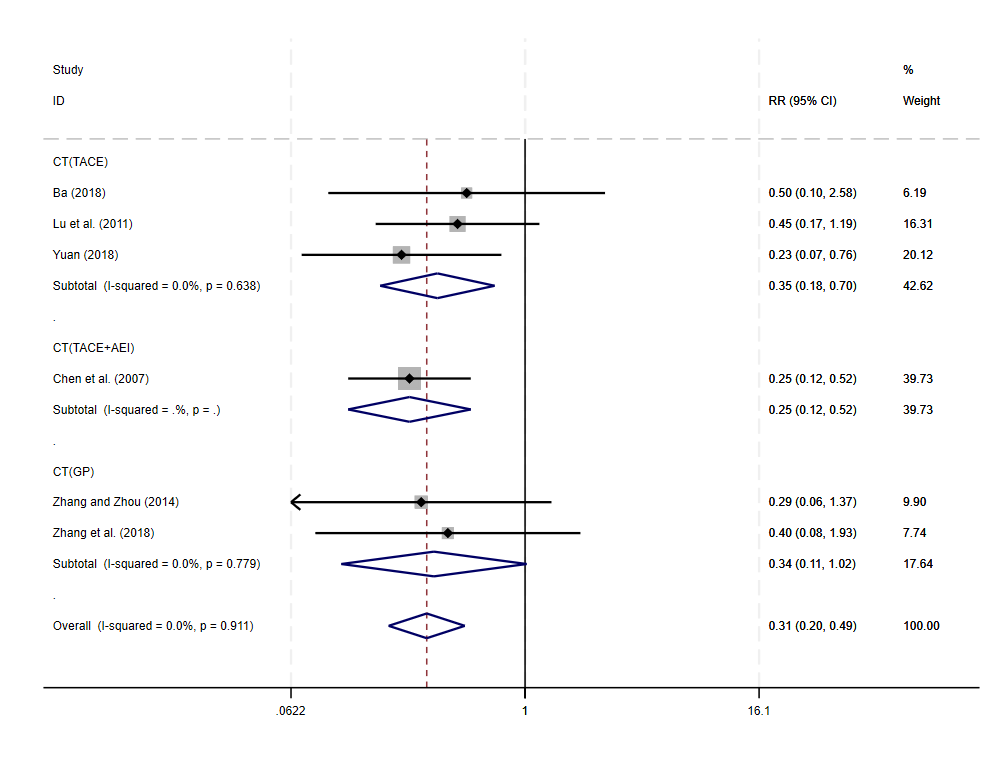


Fig. S1j. Forest plot for hepatic dysfunction of CKI combined with TACE, TACE + AEI, GP in PLC treatment

S1l


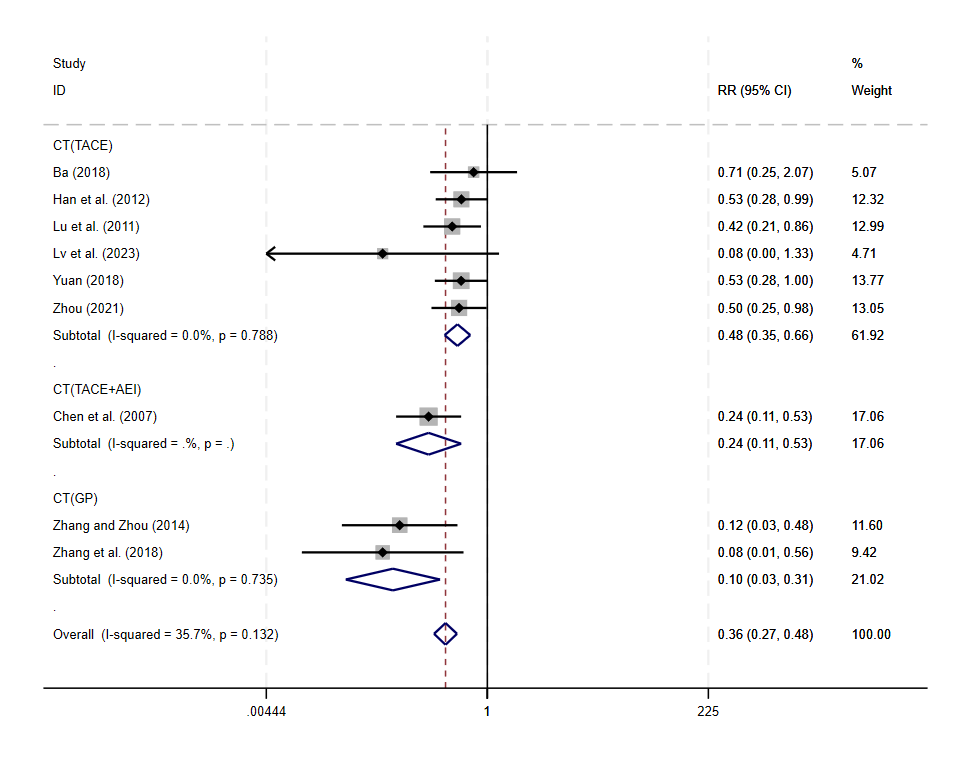


Fig. S1l. Forest plot for myelosuppression of CKI combined with TACE, TACE + AEI, GP in PLC treatment
