# Supplementary material for: Effect of compound kushen injection on immune function in patients with primary liver cancer: a systematic review and meta-analysis
Source: Front Pharmacol. 2026 Feb 19;17:1715798. doi: 10.3389/fphar.2026.1715798 (PMC12960130; doi:10.3389/fphar.2026.1715798)
Supplement: Supplementary file 3 [file Table1.docx]

Supplementary Material

**Effect of compound kushen injection on immune function in patients with primary liver cancer: a systematic review and meta-analysis**

*Yu Xiong^1,2^, Yutong Cai^3^, Chenxi Li^4^, Qian Yan^2,5*^, Xiongwen Wang^1*^, Xiaoying Zhang^6*^*

*^1^Department of Tumor, Chongqing Hospital of the First Affiliated Hospital of Guangzhou University of Chinese Medicine, Chongqing, China*

*^2^Postdoctoral Research Station, Guangzhou University of Chinese Medicine, Guangzhou, China*

*^3^School of Traditional Chinese Medicine, Chongqing University of Chinese Medicine, Chongqing, China*

*^4^School of Acupuncture and Tuina, Chongqing University of Chinese Medicine, Chongqing, China*

*^5^State Key Laboratory of Traditional Chinese Medicine, The First Affiliated Hospital of Guangzhou University of Chinese Medicine, Guangzhou, China*

*^6^Department of Tumor, Shenzhen Hospital (Futian) of Guangzhou University of Chinese Medicine, Shenzhen, China*

*^*^Corresponding authors at: The First Affiliated Hospital of Guangzhou University of Chinese Medicine, Airport Road, Baiyun District, Guangzhou ,510405, China*

*Chongqing Hospital of the First Affiliated Hospital of Guangzhou University of Chinese Medicine, No. 380, Jiangjun Road, Beibei District, Chongqing, 400700, China*

*Shenzhen Hospital (Futian) of Guangzhou University of Chinese Medicine, 6001 Beihuan Avenue, Futian District, Shenzhen, Guangdong, 518034, China*

*E-mail addresses:yanqian@gzucm.edu.cn (Q. Yan). [wangxiongwen@gzucm.edu.cn](mailto:awen681029@163.com) (X. Wang) . zhxiaoying@foxmail.com (X. Zhang).*

# Supplementary Material S1. PRISMA2020·checklist.

| **Section and Topic** | **Item #** | **Checklist item** | **Location where item is reported** |  |  |
| --- | --- | --- | --- | --- | --- |
| **TITLE** | | |  |  |  |
| Title | 1 | Identify the report as a systematic review. | **Title** |  |  |
| **ABSTRACT** | | |  |  |  |
| Abstract | 2 | See the PRISMA 2020 for Abstracts checklist. | **Abstract** |  |  |
| **INTRODUCTION** | | |  |  |  |
| Rationale | 3 | Describe the rationale for the review in the context of existing knowledge. | **Introduction** |  |  |
| Objectives | 4 | Provide an explicit statement of the objective(s) or question(s) the review addresses. | **Introduction** |  |  |
| **METHODS** | | |  |  |  |
| Eligibility criteria | 5 | Specify the inclusion and exclusion criteria for the review and how studies were grouped for the syntheses. | **Methods; Eligibility criteria** |  |  |
| Information sources | 6 | Specify all databases, registers, websites, organisations, reference lists and other sources searched or consulted to identify studies. Specify the date when each source was last searched or consulted. | **Methods; Search strategy** |  |  |
| Search strategy | 7 | Present the full search strategies for all databases, registers and websites, including any filters and limits used. | **Methods; Search strategy** |  |  |
| Selection process | 8 | Specify the methods used to decide whether a study met the inclusion criteria of the review, including how many reviewers screened each record and each report retrieved, whether they worked independently, and if applicable, details of automation tools used in the process. | **Methods;**  **Eligibility criteria** |  |  |
| Data collection process | 9 | Specify the methods used to collect data from reports, including how many reviewers collected data from each report, whether they worked independently, any processes for obtaining or confirming data from study investigators, and if applicable, details of automation tools used in the process. | **Methods;**  **Study selection and data extraction** |  |  |
| Data items | 10a | List and define all outcomes for which data were sought. Specify whether all results that were compatible with each outcome domain in each study were sought (e.g. for all measures, time points, analyses), and if not, the methods used to decide which results to collect. | **Methods;**  **Study selection and data extraction** |  |  |
|  | 10b | List and define all other variables for which data were sought (e.g. participant and intervention characteristics, funding sources). Describe any assumptions made about any missing or unclear information. | **Methods;**  **Study selection and data extraction** |  |  |
| Study risk of bias assessment | 11 | Specify the methods used to assess risk of bias in the included studies, including details of the tool(s) used, how many reviewers assessed each study and whether they worked independently, and if applicable, details of automation tools used in the process. | **Methods; Risk of bias assessment** |  |  |
| Effect measures | 12 | Specify for each outcome the effect measure(s) (e.g. risk ratio, mean difference) used in the synthesis or presentation of results. | **Methods; Statistical analysis** |  |  |
| Synthesis methods | 13a | Describe the processes used to decide which studies were eligible for each synthesis (e.g. tabulating the study intervention characteristics and comparing against the planned groups for each synthesis (item #5)). | **Methods; Statistical analysis** |  |  |
|  | 13b | Describe any methods required to prepare the data for presentation or synthesis, such as handling of missing summary statistics, or data conversions. | **Methods; Statistical analysis** |  |  |
|  | 13c | Describe any methods used to tabulate or visually display results of individual studies and syntheses. | **Methods; Statistical analysis** |  |  |
|  | 13d | Describe any methods used to synthesize results and provide a rationale for the choice(s). If meta-analysis was performed, describe the model(s), method(s) to identify the presence and extent of statistical heterogeneity, and software package(s) used. | **Methods; Statistical analysis** |  |  |
|  | 13e | Describe any methods used to explore possible causes of heterogeneity among study results (e.g. subgroup analysis, meta-regression). | **Methods; Subgroup analysis** |  |  |
|  | 13f | Describe any sensitivity analyses conducted to assess robustness of the synthesized results. | **Methods; Sensitivity analysis** |  |  |
| Reporting bias assessment | 14 | Describe any methods used to assess risk of bias due to missing results in a synthesis (arising from reporting biases). | **Methods; Subgroup analysis** |  |  |
| Certainty assessment | 15 | Describe any methods used to assess certainty (or confidence) in the body of evidence for an outcome. | **Methods; GRADE Evidence Quality Assessment** |  |  |
| **RESULTS** | | |  |  |  |
| Study selection | 16a | Describe the results of the search and selection process, from the number of records identified in the search to the number of studies included in the review, ideally using a flow diagram. | **Results; Search results and study characteristics** |  |  |
|  | 16b | Cite studies that might appear to meet the inclusion criteria, but which were excluded, and explain why they were excluded. | **Results; Search results and study characteristics** |  |  |
| Study characteristics | 17 | Cite each included study and present its characteristics. | **Results; Search results and study characteristics** |  |  |
| Risk of bias in studies | 18 | Present assessments of risk of bias for each included study. | **Results; Search results and study characteristics** |  |  |
| Results of individual studies | 19 | For all outcomes, present, for each study: (a) summary statistics for each group (where appropriate) and (b) an effect estimate and its precision (e.g. confidence/credible interval), ideally using structured tables or plots. | **Results; Search results and study characteristics** |  |  |
| Results of syntheses | 20a | For each synthesis, briefly summarise the characteristics and risk of bias among contributing studies. | **Results; Risk of bias Assessment** |  |  |
|  | 20b | Present results of all statistical syntheses conducted. If meta-analysis was done, present for each the summary estimate and its precision (e.g. confidence/credible interval) and measures of statistical heterogeneity. If comparing groups, describe the direction of the effect. | **Results; Primary Outcomes,Secondary Outcomes** |  |  |
|  | 20c | Present results of all investigations of possible causes of heterogeneity among study results. | **Results; publication bias** |  |  |
|  | 20d | Present results of all sensitivity analyses conducted to assess the robustness of the synthesized results. | **Results; Sensitivity analysis** |  |  |
| Reporting biases | 21 | Present assessments of risk of bias due to missing results (arising from reporting biases) for each synthesis assessed. | **Results; publication bias** |  |  |
| Certainty of evidence | 22 | Present assessments of certainty (or confidence) in the body of evidence for each outcome assessed. | **Results; Quality classification by GRADE** |  |  |
| **DISCUSSION** | | |  |  |  |
| Discussion | 23a | Provide a general interpretation of the results in the context of other evidence. | **Discussion** |  |  |
|  | 23b | Discuss any limitations of the evidence included in the review. | **Discussion** |  |  |
|  | 23c | Discuss any limitations of the review processes used. | **Discussion** |  |  |
|  | 23d | Discuss implications of the results for practice, policy, and future research. | **Discussion** |  |  |
| **OTHER INFORMATION** | | |  |  |  |
| Registration and protocol | 24a | Provide registration information for the review, including register name and registration number, or state that the review was not registered. | **Methods** |  |  |
|  | 24b | Indicate where the review protocol can be accessed, or state that a protocol was not prepared. | **Conclusions** |  |  |
|  | 24c | Describe and explain any amendments to information provided at registration or in the protocol. | **Conclusions** |  |  |
| Support | 25 | Describe sources of financial or non-financial support for the review, and the role of the funders or sponsors in the review. | **Funding** |  |  |
| Competing interests | 26 | Declare any competing interests of review authors. | **Acknowledgments** |  |  |
| Availability of data, code and other materials | 27 | Report which of the following are publicly available and where they can be found: template data collection forms; data extracted from included studies; data used for all analyses; analytic code; any other materials used in the review. | **Data availability** |  |  |
| **Deviations from Protocol** | | | |  |  |
| Synthesis methods | 28 | Subgroup analyses of different intervention regimens and dosages for CKI treatment were added, along with subgroup analyses and sensitivity analyses based on different efficacy evaluation criteria. | Figure S1、S2、S3 |  |  |

Page MJ, McKenzie JE, Bossuyt PM, et al. The PRISMA 2020 statement: an updated guideline for reporting systematic reviews[J]. BMJ. 2021, 372: n71.

# Supplementary Material S2. The search strategy.

**Search run on October 31 2024**

**PubMed (*n*=20)**

#1 ((Compound Kushen Injection [Title/Abstract]) OR (Compound Sophora flavescens Injection [Title/Abstract]) OR (Yanshu injection [Title/Abstract]))

#2 ((hepatocellular carcinoma [Title/Abstract]) OR (liver cancer [Title/Abstract]) OR (Hepatoma [Title/Abstract]) OR (Liver Cell Carcinomas [Title/Abstract]))

#3 #1 AND #2

**Embase (*n* =18 )**

#1 'Compound Kushen Injection'/exp

#2 'Compound Sophora flavescens Injection':ab,ti OR 'Yanshu Injection':ab,ti

#3 #1 OR #2

#4 'hepatocellular carcinoma'/exp

#5 'hepatocellular carcinoma':ab,ti OR 'liver cancer':ab,ti OR 'Hepatoma':ab,ti OR 'Liver Cell Carcinomas':ab,ti

#6 #4 OR #5

#7 #3 AND #6

**Cochrane library (*n* = 12)**

#1 MeSH descriptor: [Compound Kushen Injection] explode all trees

#2 (Compound Sophora flavescens Injection):ti,ab,kw OR (Yanshu Injection):ti,ab,kw

#3 #1 OR #2

#4 MeSH descriptor: [hepatocellular carcinoma] explode all trees

#5 (liver cancer):ti,ab,kw OR (Hepatoma):ti,ab,kw OR (Liver Cell Carcinomas):ti,ab,kw

#6 #4 OR #5

#7 #3 AND #6

**Web of Science (*n* = 41)**

#1 (TS=(Compound Kushen Injection) OR TS=(Compound Sophora flavescens Injection) OR TS=(Yanshu injection ))

#2 (TS=(hepatocellular carcinoma) OR TS=(liver cancer) OR TS=(Hepatoma) OR TS=(Liver Cell Carcinomas))

#3 #1 AND #2

**CNKI (*n*=256)**

#1 篇关摘：复方苦参注射液 OR 苦参注射液OR 岩舒注射液

#2 篇关摘：原发性肝癌 OR 肝细胞癌 OR 肝恶性肿瘤 OR肝肿瘤 OR肝癌

#3 #1 AND #2

(TKA=复方苦参注射液 OR TKA=苦参注射液 OR TKA=岩舒注射液) AND (TKA=原发性肝癌 OR TKA=肝细胞癌 OR TKA=肝恶性肿瘤 OR TKA=肝肿瘤 OR TKA=肝癌)

**Wanfang Data (*n*=164)**

#1 主题：复方苦参注射液 OR 苦参注射液 OR 岩舒注射液

#2 主题：原发性肝癌 OR 肝细胞癌 OR 肝恶性肿瘤 OR肝肿瘤 OR肝癌

#3 #1 AND #2

**CQVIP (*n*=181)**

#1 篇关摘：复方苦参注射液 OR 苦参注射液 OR 岩舒注射液

#2 篇关摘：原发性肝癌 OR 肝细胞癌 OR 肝恶性肿瘤 OR肝肿瘤 OR肝癌

#3 #1 AND #2

**CBM (*n*=220)**

#1 常用字段：复方苦参注射液 OR 苦参注射液 OR 岩舒注射液

#2 常用字段：原发性肝癌 OR 肝细胞癌 OR 肝恶性肿瘤 OR肝肿瘤 OR肝癌

#3 #1 AND #2

**Other sources (*n*=1)**

# Supplementary Material S3. Quality assessment of included studies.

| Study ID | Random sequence generation | Allocation concealment | Blinding | | Incomplete outcome data | Selective reporting | Other biases | Modified Jadad scores |
| --- | --- | --- | --- | --- | --- | --- | --- | --- |
|  |  |  | Blinding of participants and personnel | Blinding of outcome assessment |  |  |  |  |
| Ba (2018) | Low risk | Unclear risk | Unclear risk | Unclear risk | Low risk | Low risk | Unclear risk | 3 |
| Cao et al. (2011) | Unclear risk | Unclear risk | Unclear risk | Unclear risk | Low risk | Low risk | Unclear risk | 2 |
| Chen et al. (2007) | Unclear risk | Unclear risk | Unclear risk | Unclear risk | Low risk | Low risk | Unclear risk | 2 |
| Dong et al. (2016) | Low risk | Unclear risk | Unclear risk | Unclear risk | Low risk | Low risk | Unclear risk | 3 |
| Guan et al. (2006) | Unclear risk | Unclear risk | Unclear risk | Unclear risk | Low risk | Low risk | Unclear risk | 2 |
| Guo (2015) | Unclear risk | Unclear risk | Unclear risk | Unclear risk | Low risk | Low risk | Unclear risk | 2 |
| Han et al. (2012) | Low risk | Unclear risk | Unclear risk | Unclear risk | Low risk | Low risk | Unclear risk | 3 |
| Hao et al. (2020) | Low risk | Unclear risk | Unclear risk | Unclear risk | Low risk | Low risk | Unclear risk | 3 |
| He et al. (2016) | High risk | Unclear risk | Unclear risk | Unclear risk | Low risk | Low risk | Unclear risk | 1 |
| Jiang et al. (2017) | Unclear risk | Unclear risk | Unclear risk | Unclear risk | Low risk | Low risk | Unclear risk | 2 |
| Li (2020) | Low risk | Unclear risk | Unclear risk | Unclear risk | Low risk | Low risk | Unclear risk | 3 |
| Li and Wang (2021) | Unclear risk | Unclear risk | Unclear risk | Unclear risk | Low risk | Low risk | Unclear risk | 2 |
| Liu et al. (2024) | Low risk | Unclear risk | Unclear risk | Unclear risk | Low risk | Low risk | Unclear risk | 3 |
| Lu et al. (2011) | Unclear risk | Unclear risk | Unclear risk | Unclear risk | Low risk | Low risk | Unclear risk | 2 |
| Lv et al. (2023) | Low risk | Unclear risk | Unclear risk | Unclear risk | Low risk | Low risk | Unclear risk | 3 |
| Ren and Feng (2024) | Low risk | Unclear risk | Unclear risk | Unclear risk | Low risk | Low risk | Unclear risk | 3 |
| Wang (2009) | High risk | Unclear risk | Unclear risk | Unclear risk | Low risk | Low risk | Unclear risk | 1 |
| Xu et al. (2012) | High risk | Unclear risk | Unclear risk | Unclear risk | Low risk | Low risk | Unclear risk | 1 |
| Yao et al. (2021) | Unclear risk | Unclear risk | Unclear risk | Unclear risk | Low risk | Low risk | Unclear risk | 2 |
| You et al. (2018) | Unclear risk | Unclear risk | Unclear risk | Unclear risk | Low risk | Low risk | Unclear risk | 2 |
| Yuan (2018) | Unclear risk | Unclear risk | Unclear risk | Unclear risk | Low risk | Low risk | Unclear risk | 2 |
| Zhang and Zhou (2014) | High risk | Unclear risk | Unclear risk | Unclear risk | Low risk | Low risk | Unclear risk | 1 |
| Zhang et al. (2017) | Unclear risk | Unclear risk | Unclear risk | Unclear risk | Low risk | Low risk | Unclear risk | 2 |
| Zhang et al. (2018) | High risk | Unclear risk | Unclear risk | Unclear risk | Low risk | Low risk | Unclear risk | 1 |
| Zhou (2021) | Low risk | Unclear risk | Unclear risk | Unclear risk | Low risk | Low risk | Unclear risk | 3 |
