# Supplementary material for: Effect of compound kushen injection on immune function in patients with primary liver cancer: a systematic review and meta-analysis
Source: Front Pharmacol. 2026 Feb 19;17:1715798. doi: 10.3389/fphar.2026.1715798 (PMC12960130; doi:10.3389/fphar.2026.1715798)
Supplement: Supplementary file 4 [file Supplementaryfile5.docx]

Figure S5

**Depression**

S5a


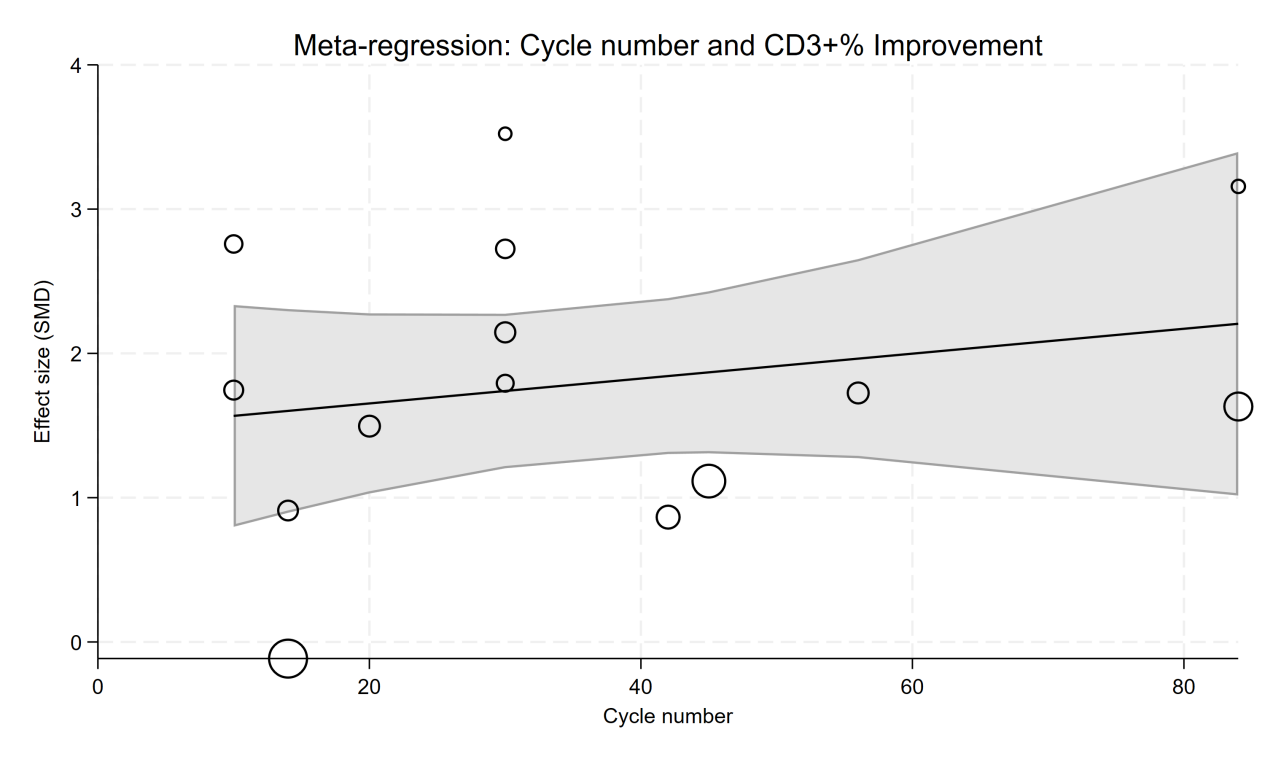


Fig. S5a. Regression analysis for CD3^+^ levels.

S5b


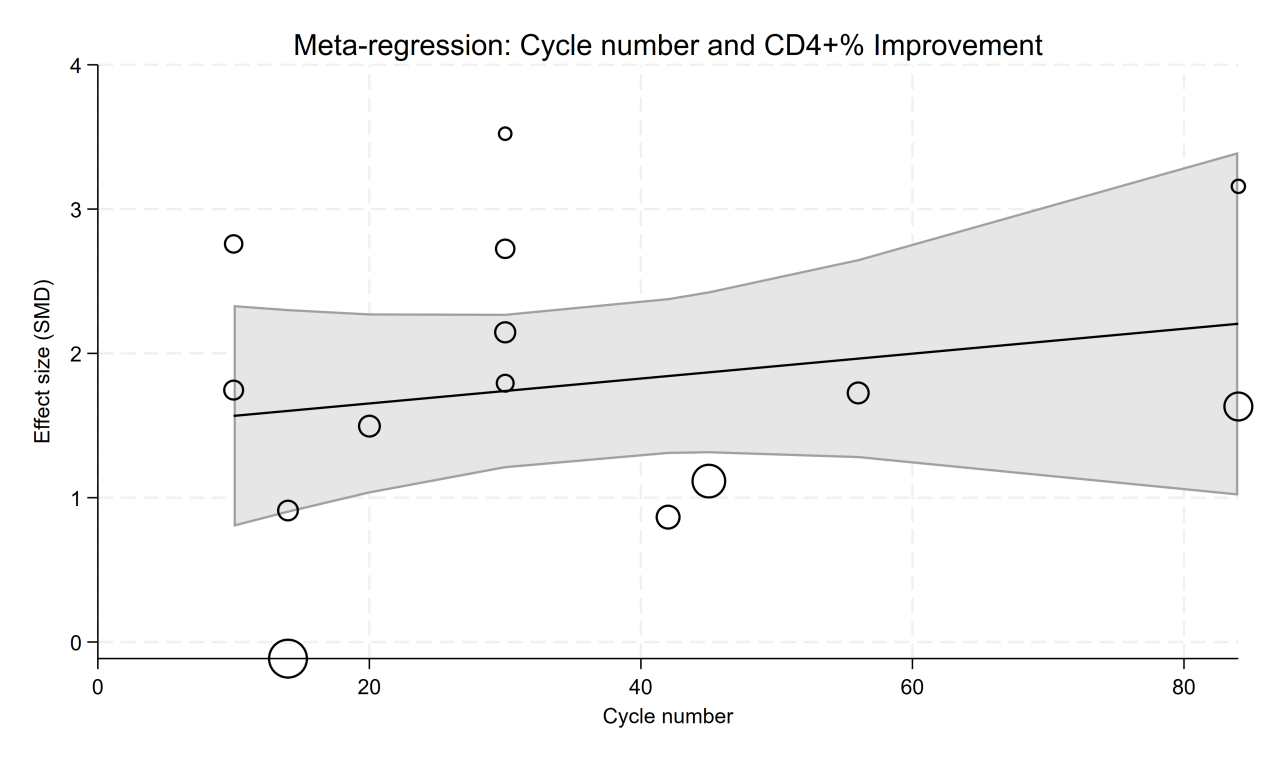


Fig. S5b. Regression analysis for CD4^+^ levels.

S5c


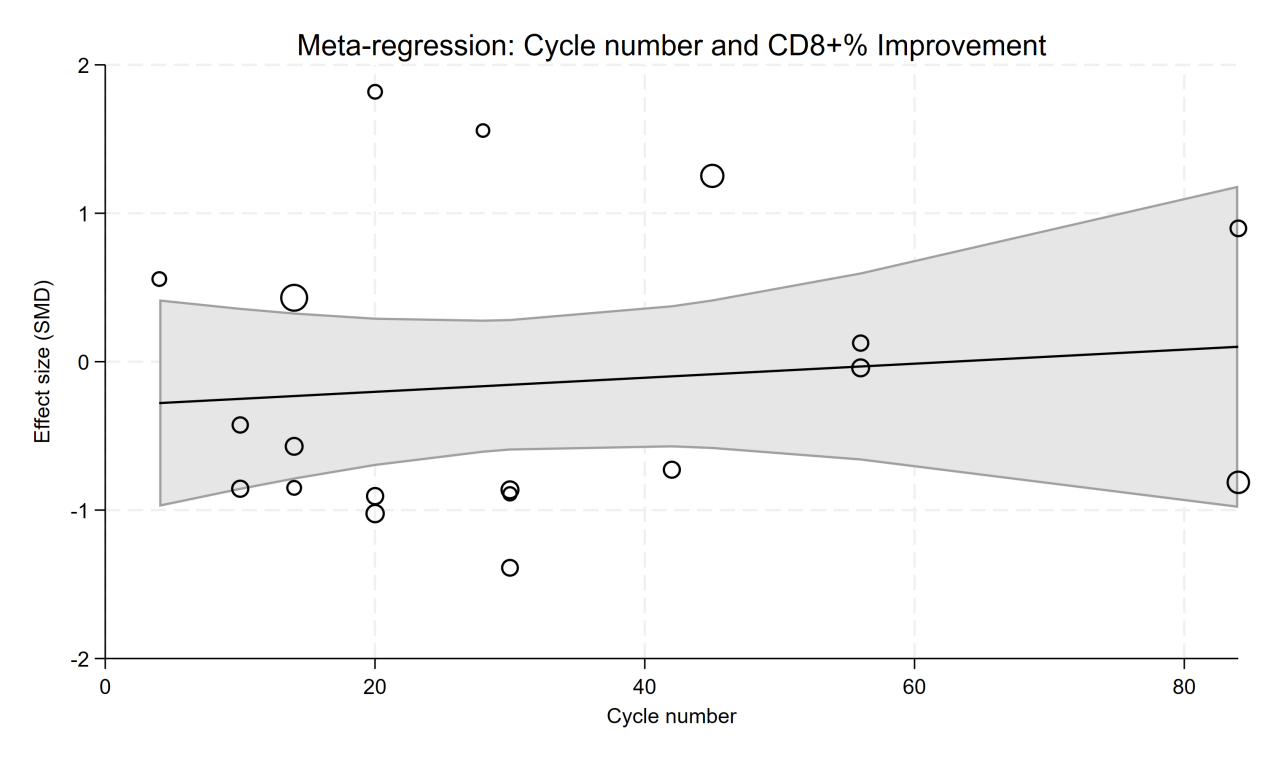


Fig. S5c. Regression analysis for CD8^+^ levels.

S5d


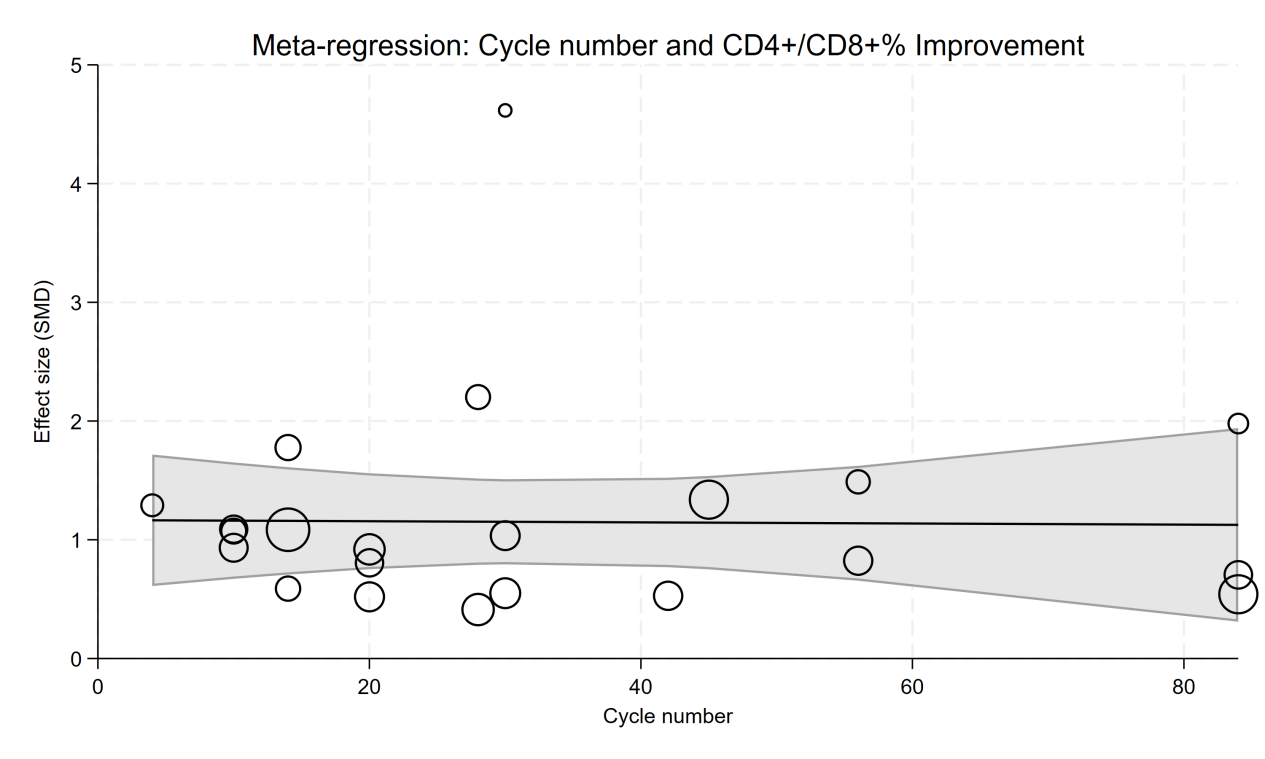


Fig. S5d. Regression analysis for CD4^+^/ CD8^+^ ratio.

S5e


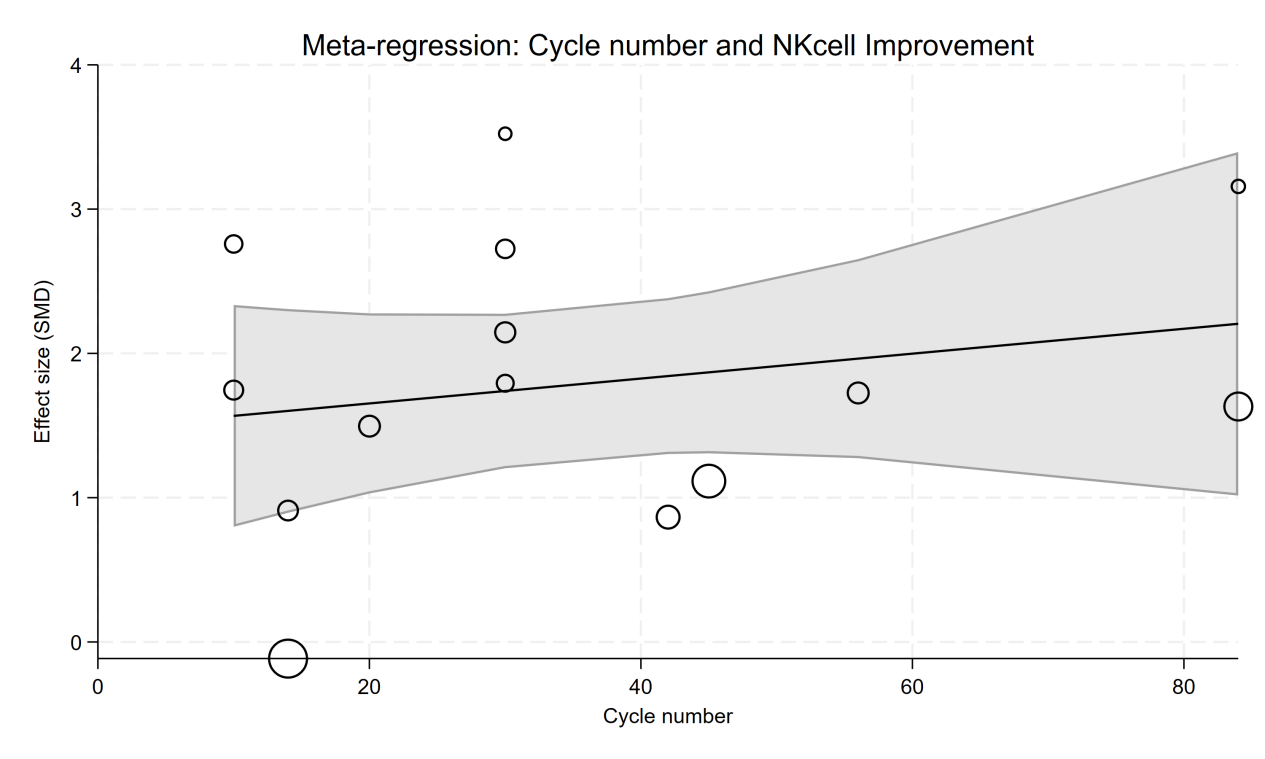


Fig. S5e. Regression analysis for NK cell levels.

S5f


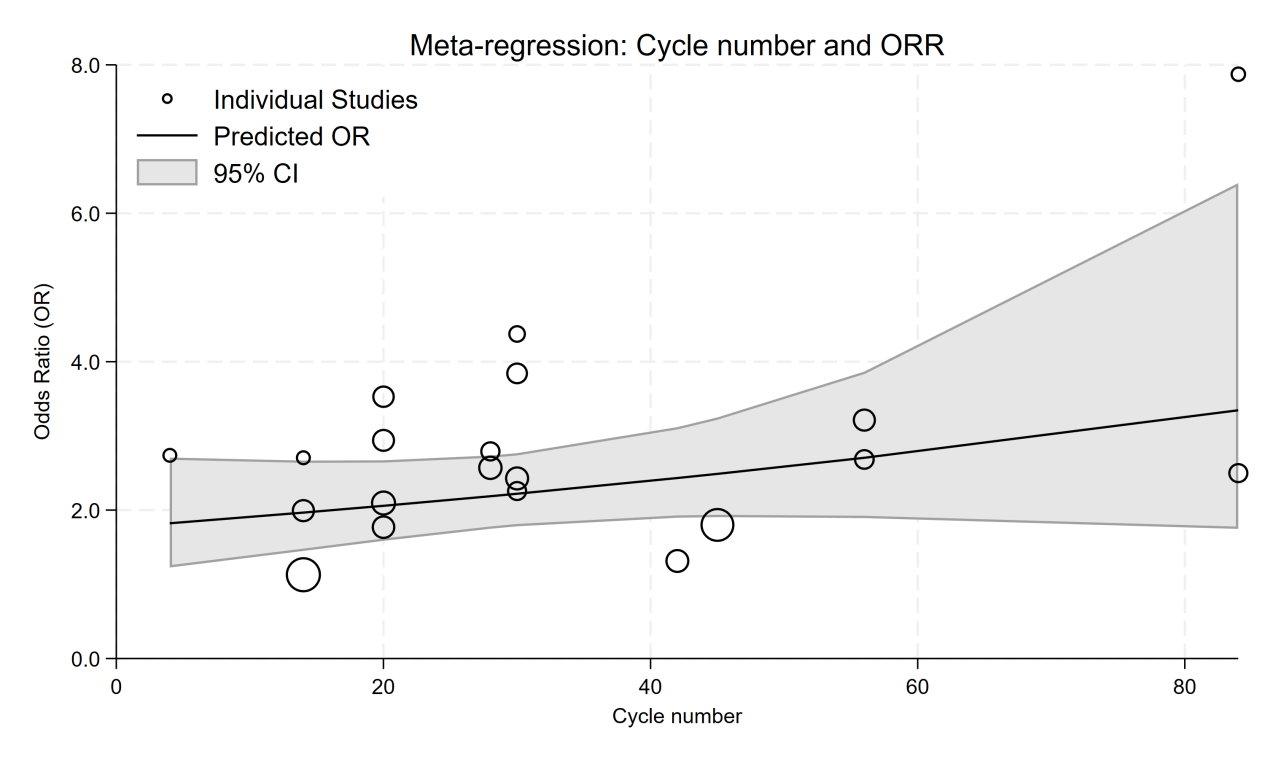


Fig. S5f. Regression analysis for ORR.

S5g.


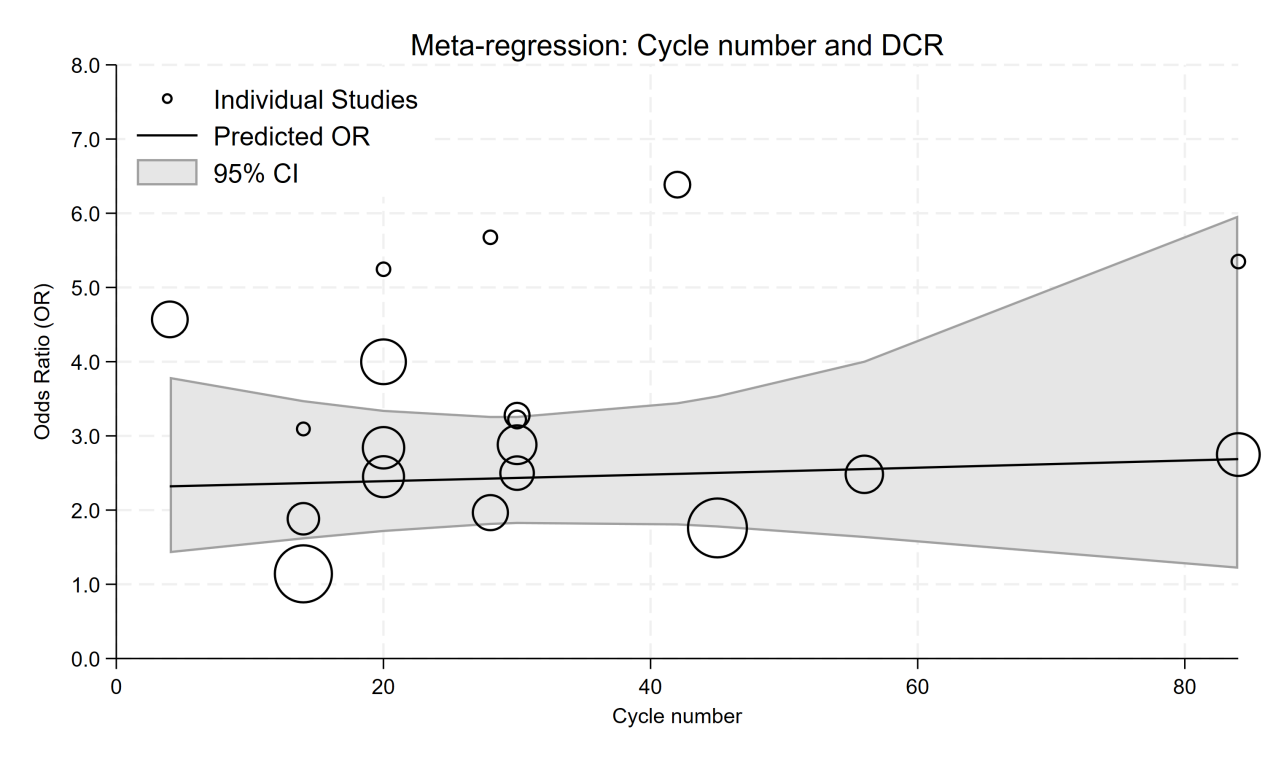


Fig. S5g. Regression analysis for DCR.

S5h


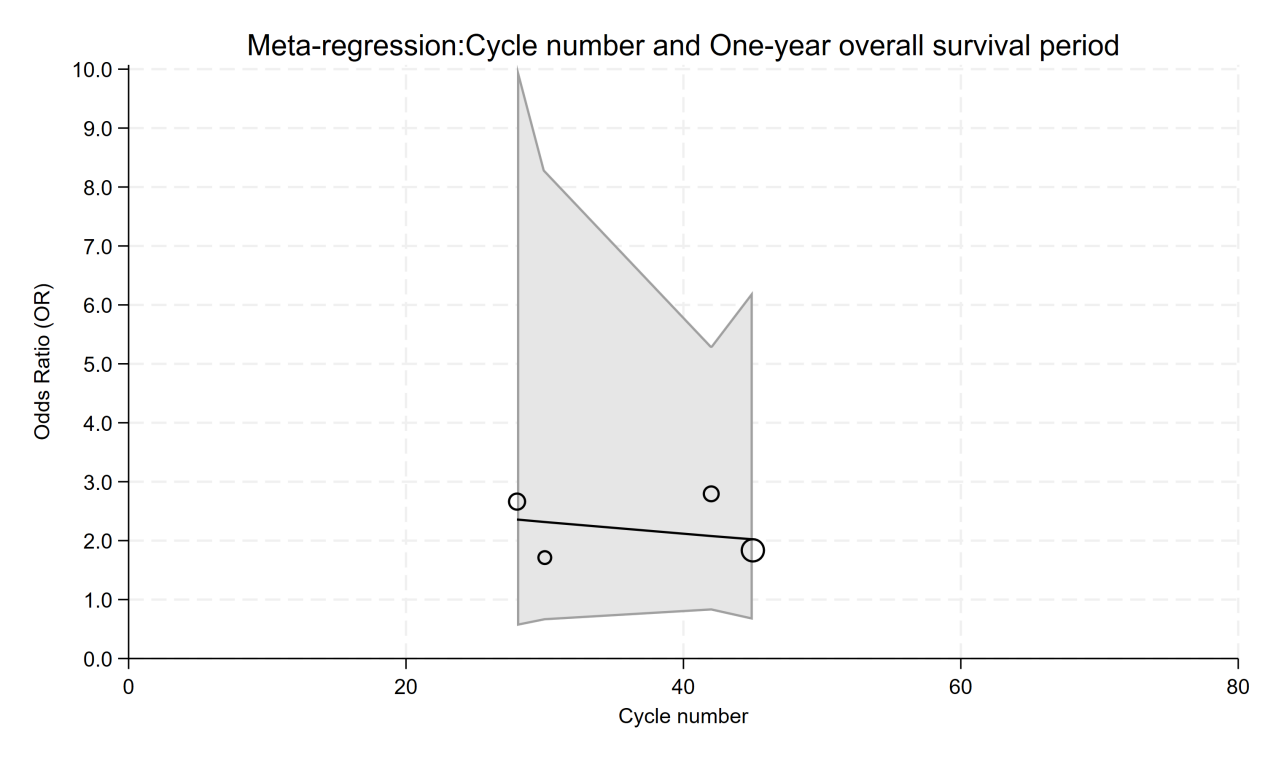


Fig. S5h. Regression analysis for one-year overall survival period.

.
