# Supplementary figures and images for: Effect of compound kushen injection on immune function in patients with primary liver cancer: a systematic review and meta-analysis
Source: Front Pharmacol. 2026 Feb 19;17:1715798. doi: 10.3389/fphar.2026.1715798 (PMC12960130; doi:10.3389/fphar.2026.1715798)

Figure S3

**Depression**

S3a

Fig. S3a. Sensitivity analysis for ORR.


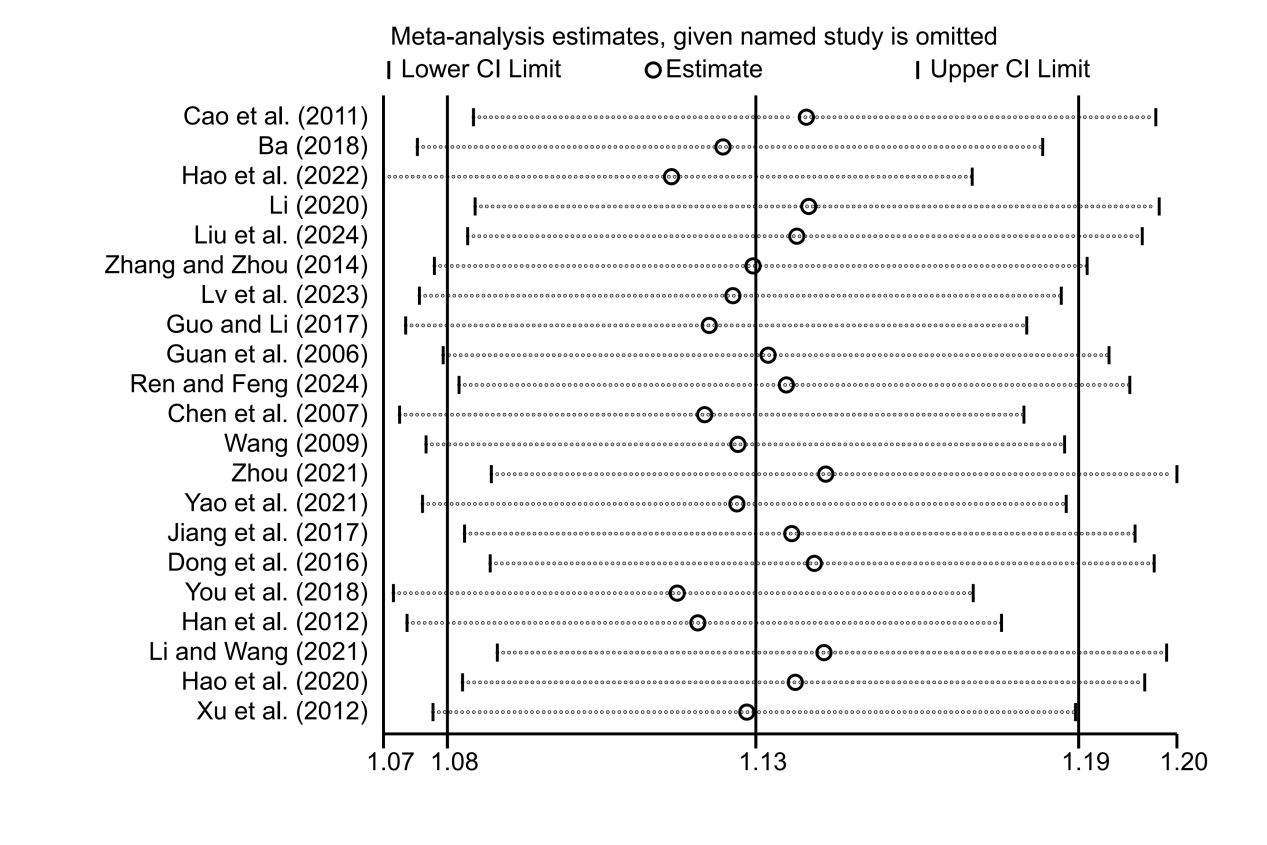


Fig. S3b. Sensitivity analysis for DCR.

Supplement: Supplementary file 5 [file Supplementaryfile3.docx]

Table S2

**Depression**

Table. S2a. GRADE evidence profile


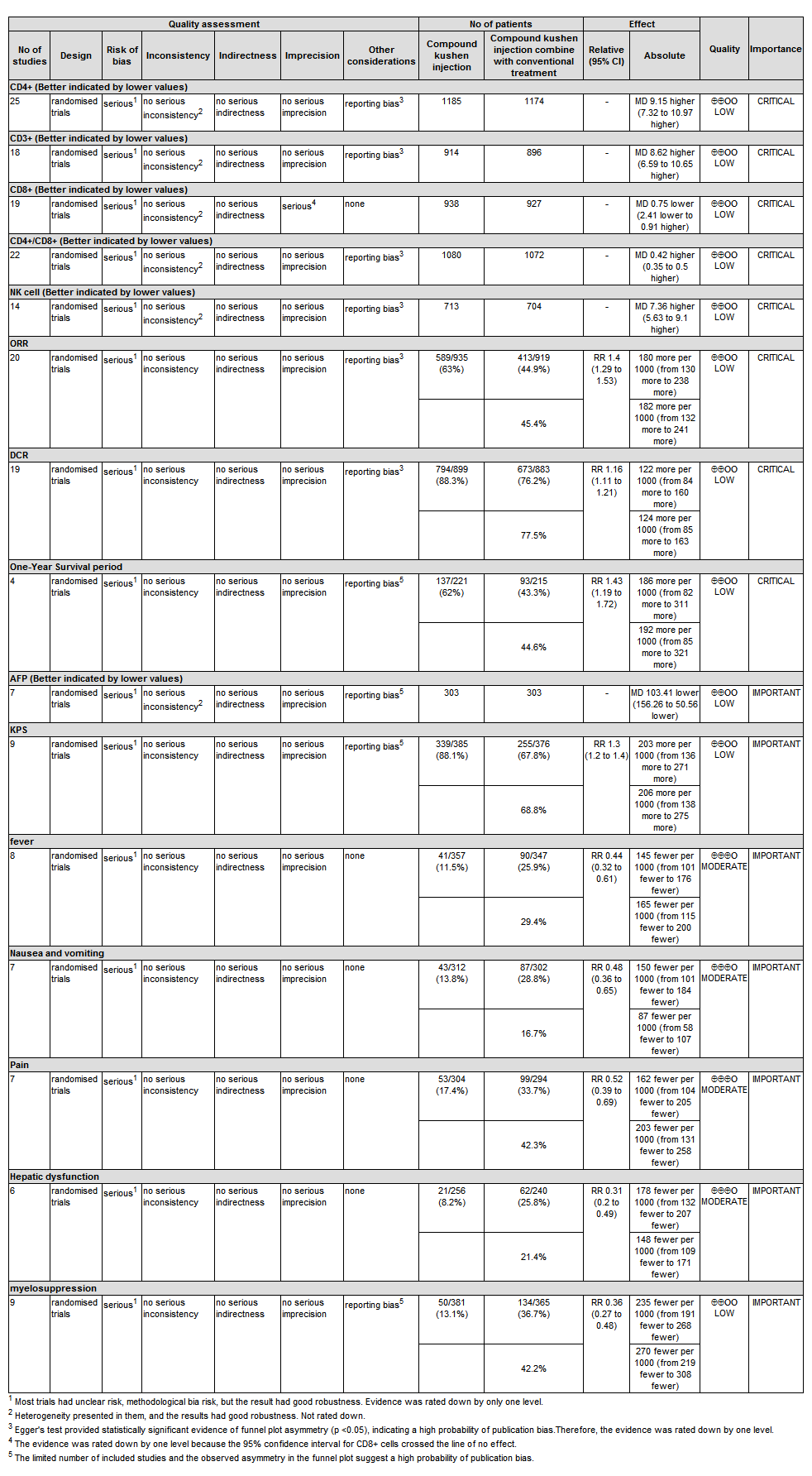


Table. S2b. Summary of Findings table(SoF)


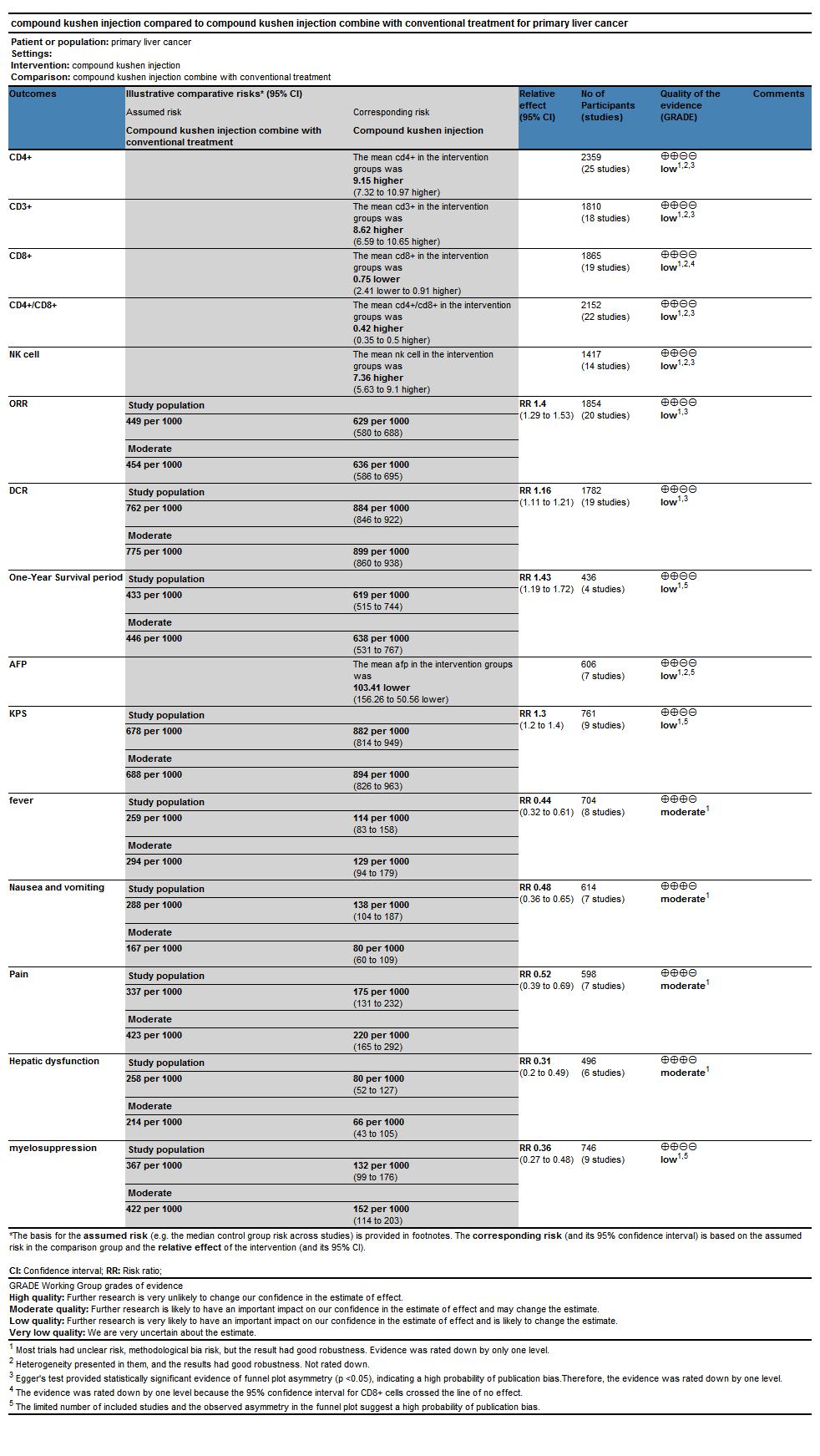

Supplement: Supplementary file 6 [file Table3.docx]
