# Supplementary material for: Effect of compound kushen injection on immune function in patients with primary liver cancer: a systematic review and meta-analysis
Source: Front Pharmacol. 2026 Feb 19;17:1715798. doi: 10.3389/fphar.2026.1715798 (PMC12960130; doi:10.3389/fphar.2026.1715798)
Supplement: Supplementary file 7 [file Supplementaryfile2.docx]

Figure S2

**Depression**

S2a


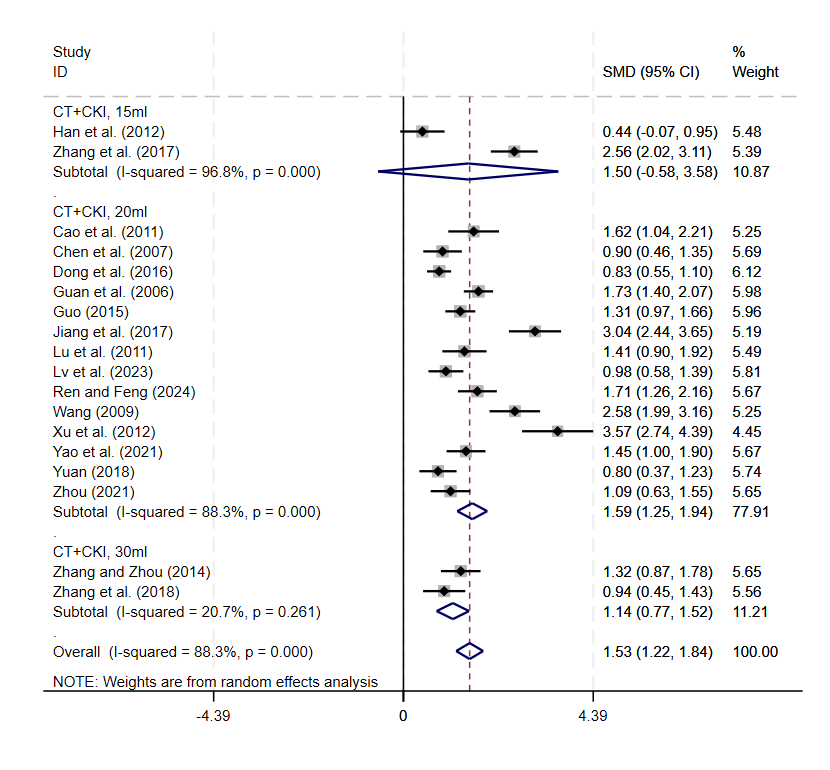


Fig. S2a. Forest plot for CD3^+^ levels of CKI dose.

S2b


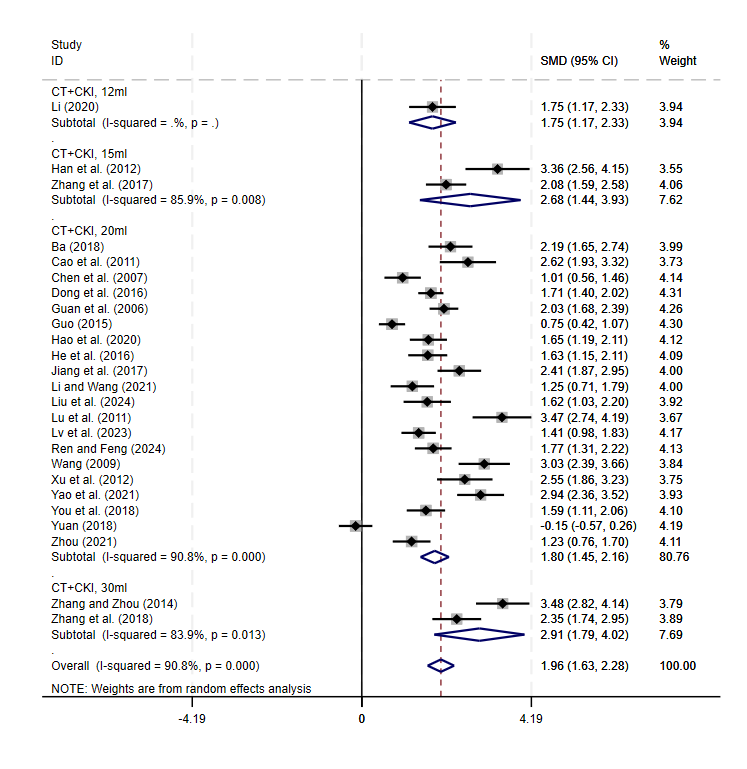


Fig. S2b. Forest plot for CD4^+^ levels of CKI dose.

S2c
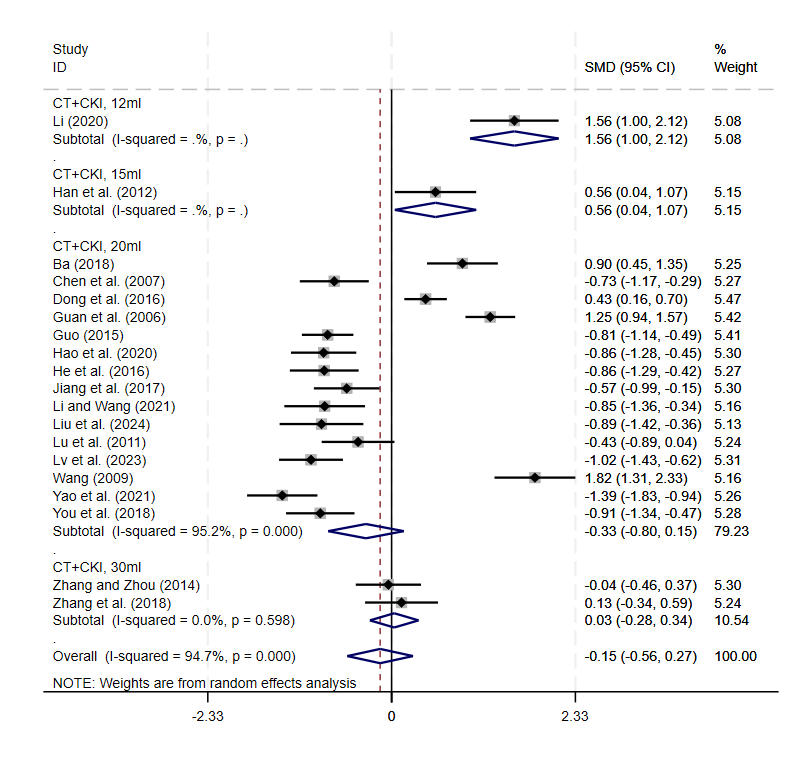


Fig. S2c. Forest plot for CD8^+^ levels of CKI dose.

S2d


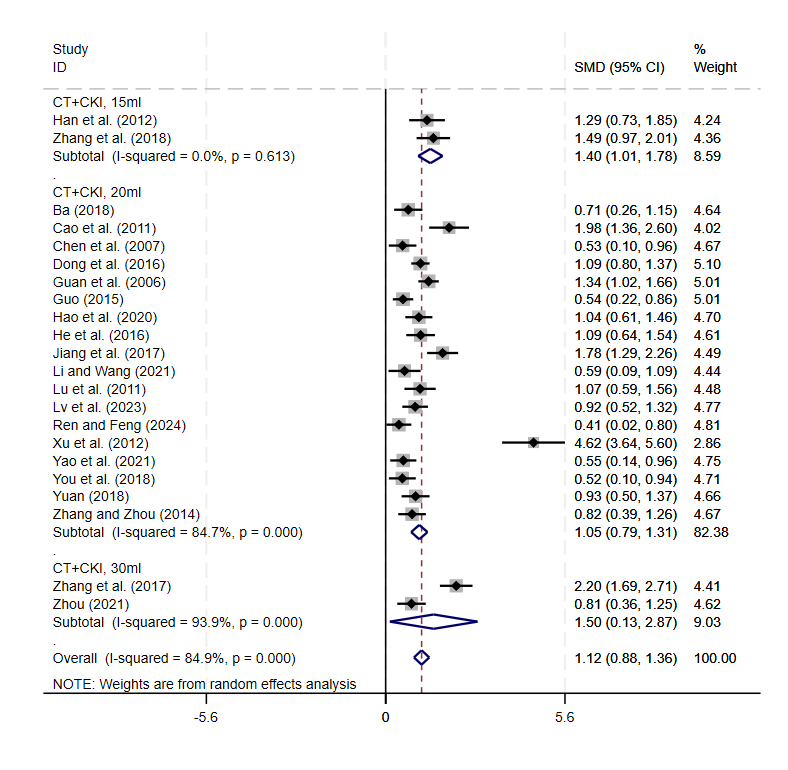


Fig. S2d. Forest plot for CD4^+^/CD8^+^ ratio of CKI dose.

S2e
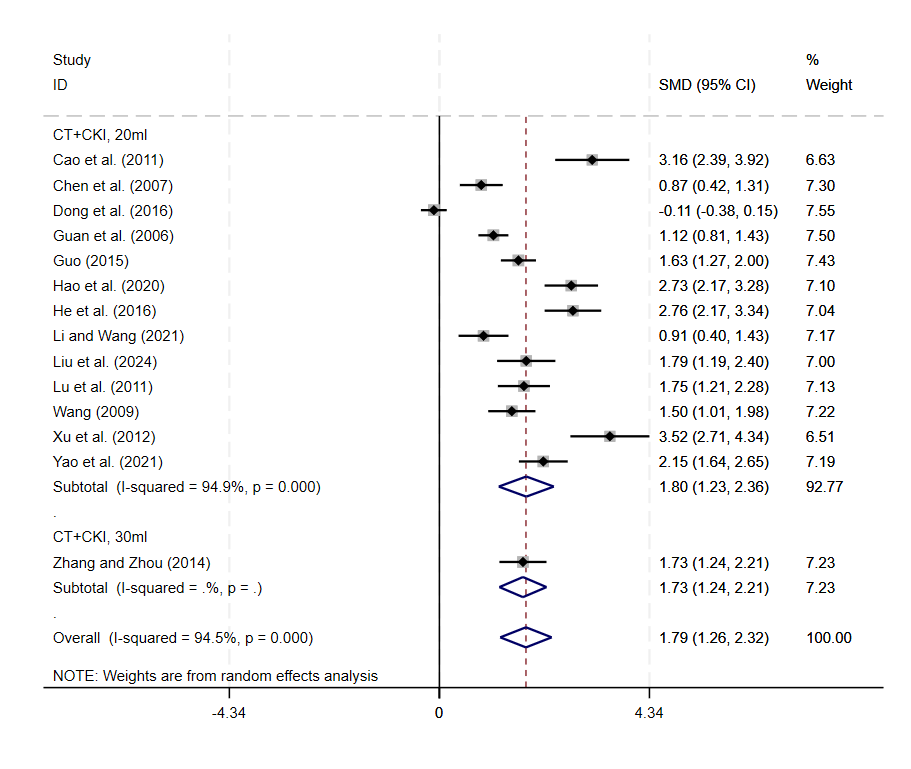


Fig. S2e. Forest plot for NK cell levels of CKI dose.
