# Supplementary material for: Effect of compound kushen injection on immune function in patients with primary liver cancer: a systematic review and meta-analysis
Source: Front Pharmacol. 2026 Feb 19;17:1715798. doi: 10.3389/fphar.2026.1715798 (PMC12960130; doi:10.3389/fphar.2026.1715798)
Supplement: Supplementary file 8 [file Supplementaryfile4.docx]

Figure S4

**Depression**

S4a


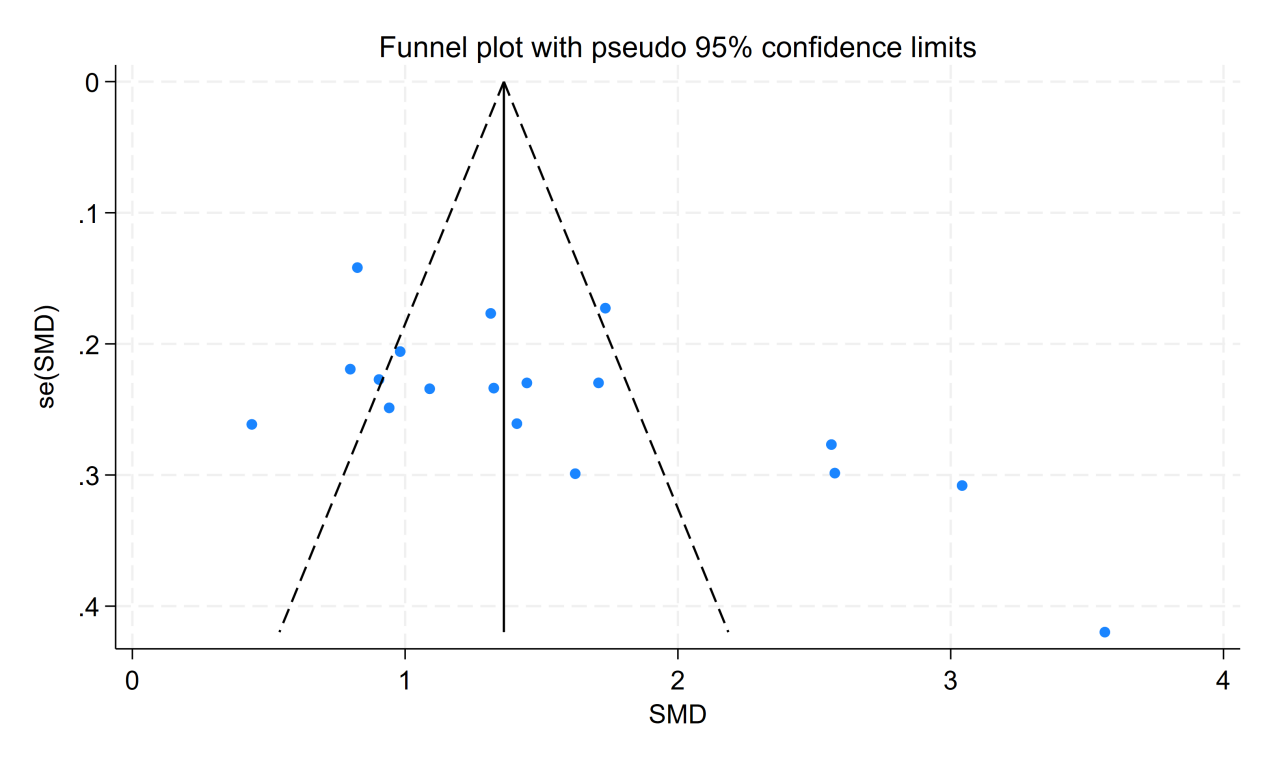


Fig. S4a. Funnel plot for CD3^+^ levels.

S4b


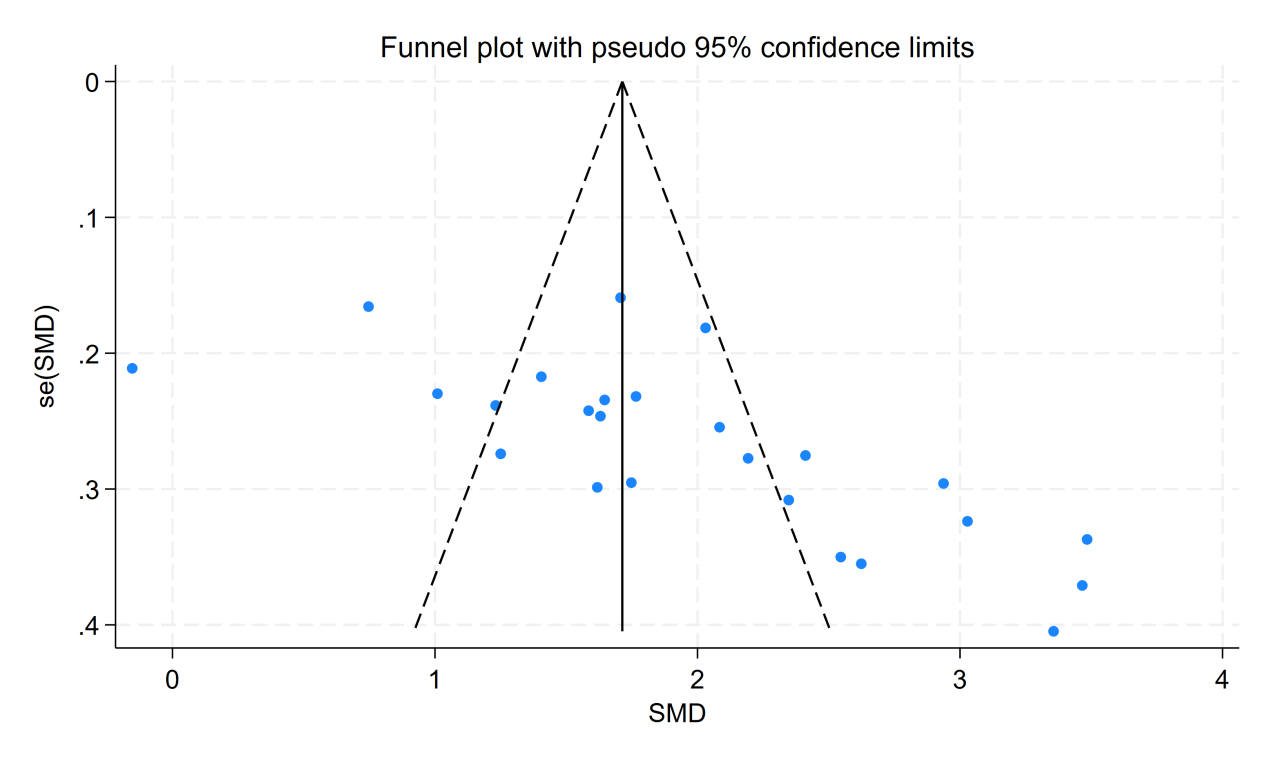


Fig. S4b. Funnel plot for CD4^+^ levels.

S4c


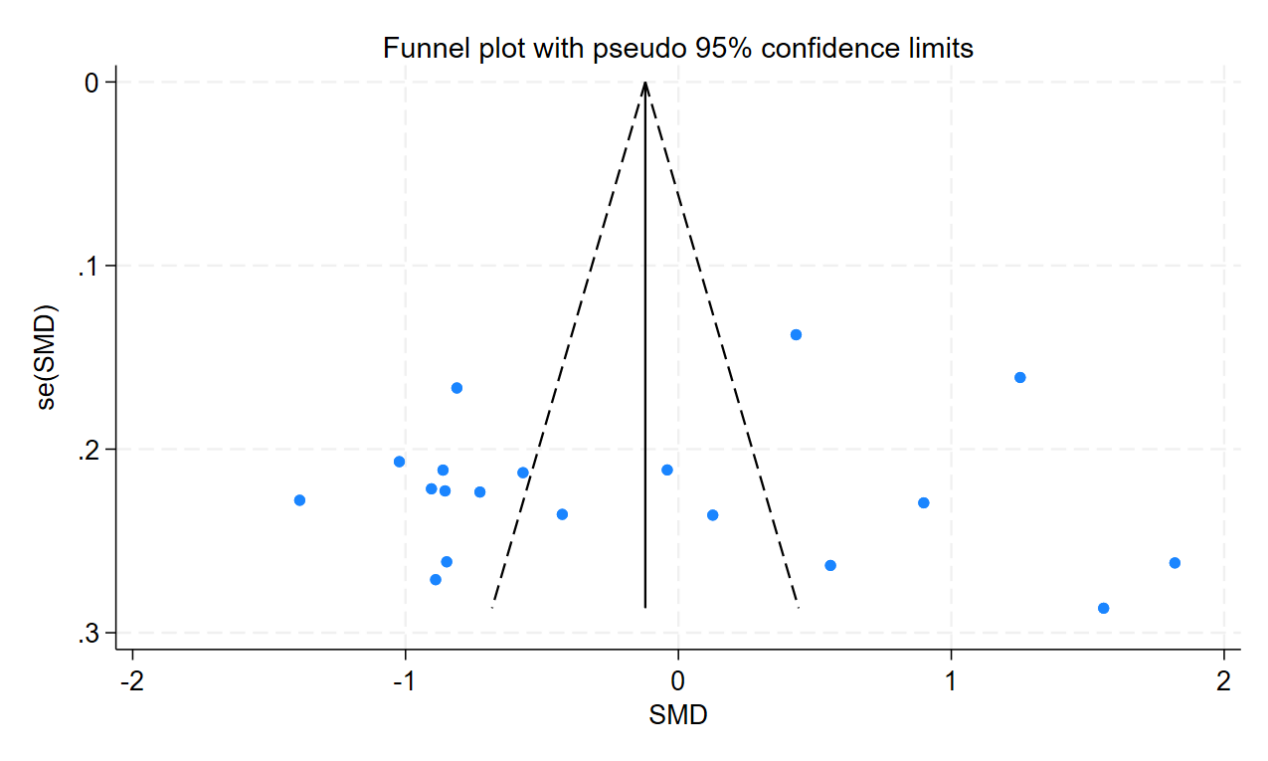


Fig. S4c. Funnel plot for CD8^+^ levels.

S4d


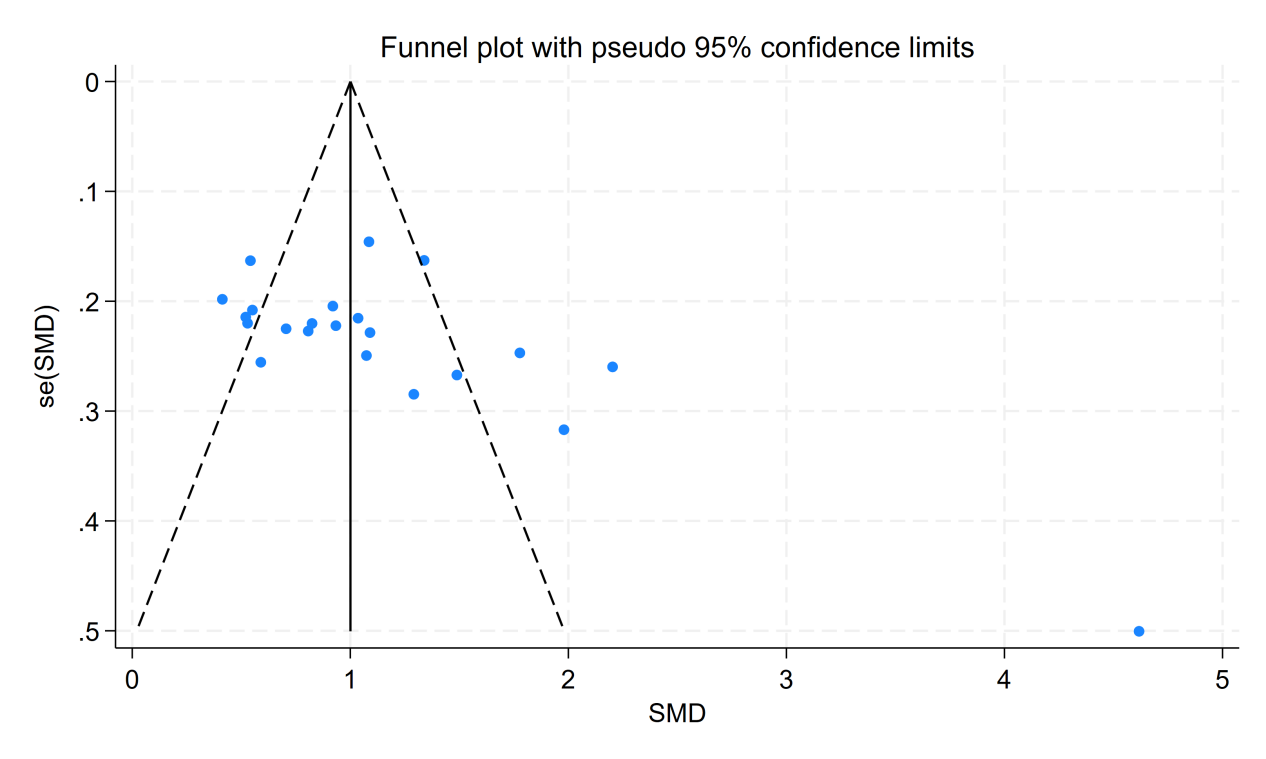


Fig. S4d. Funnel plot for CD4^+^/CD8^+^ ratio.

S4e


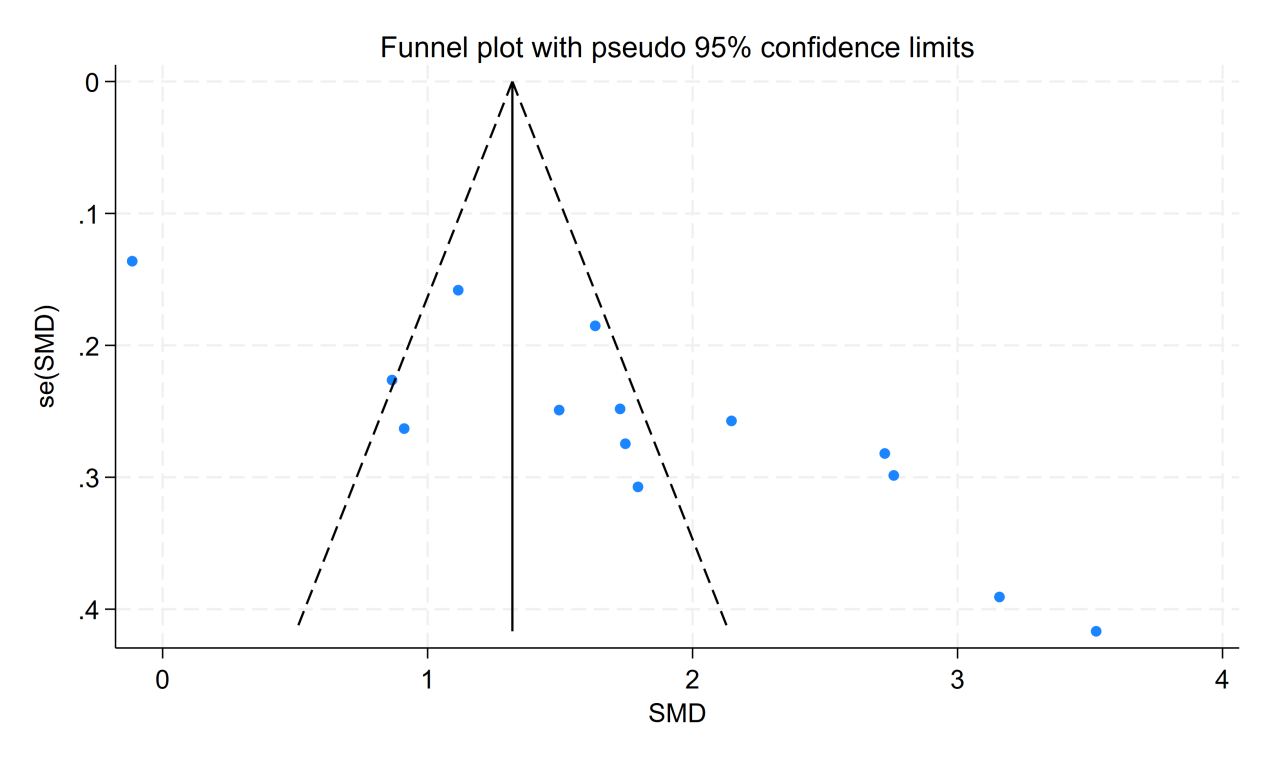


Fig. S4e. Funnel plot for NK cell^+^ levels.

S4f


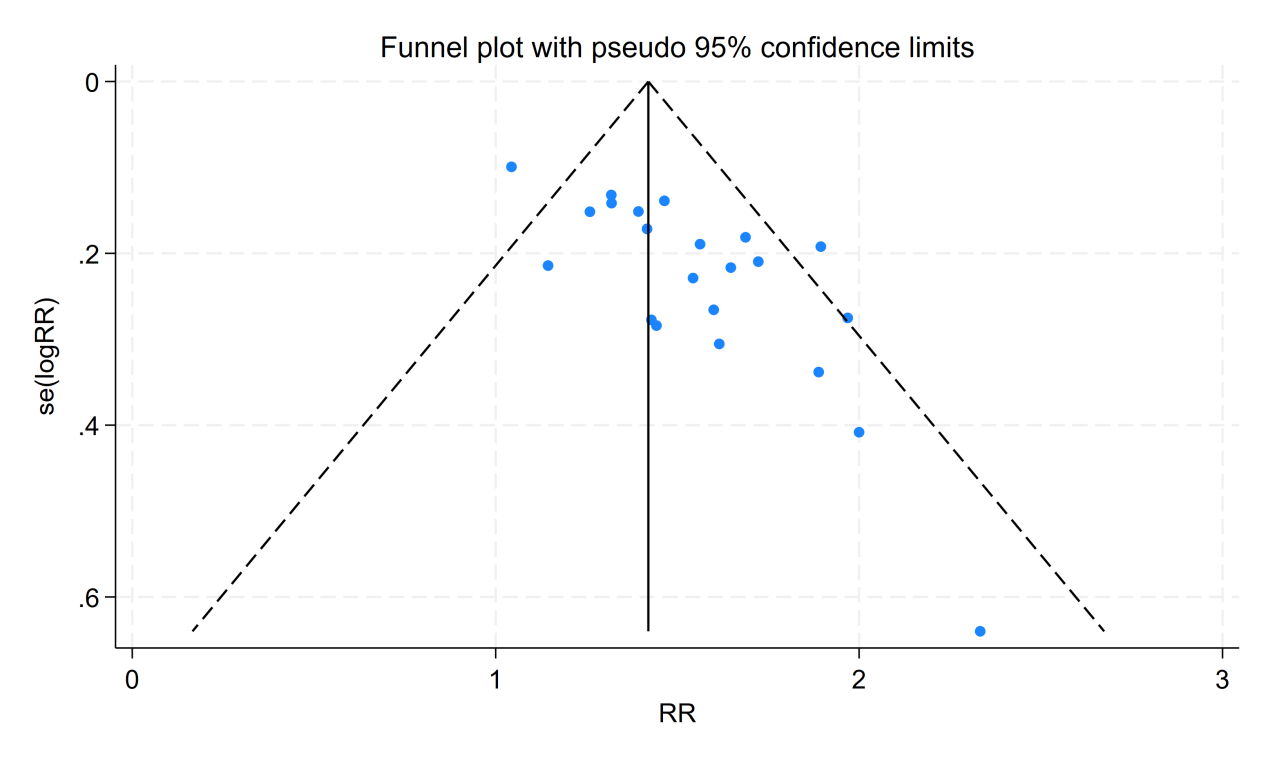


Fig. S4f. Funnel plot for ORR.

S4g


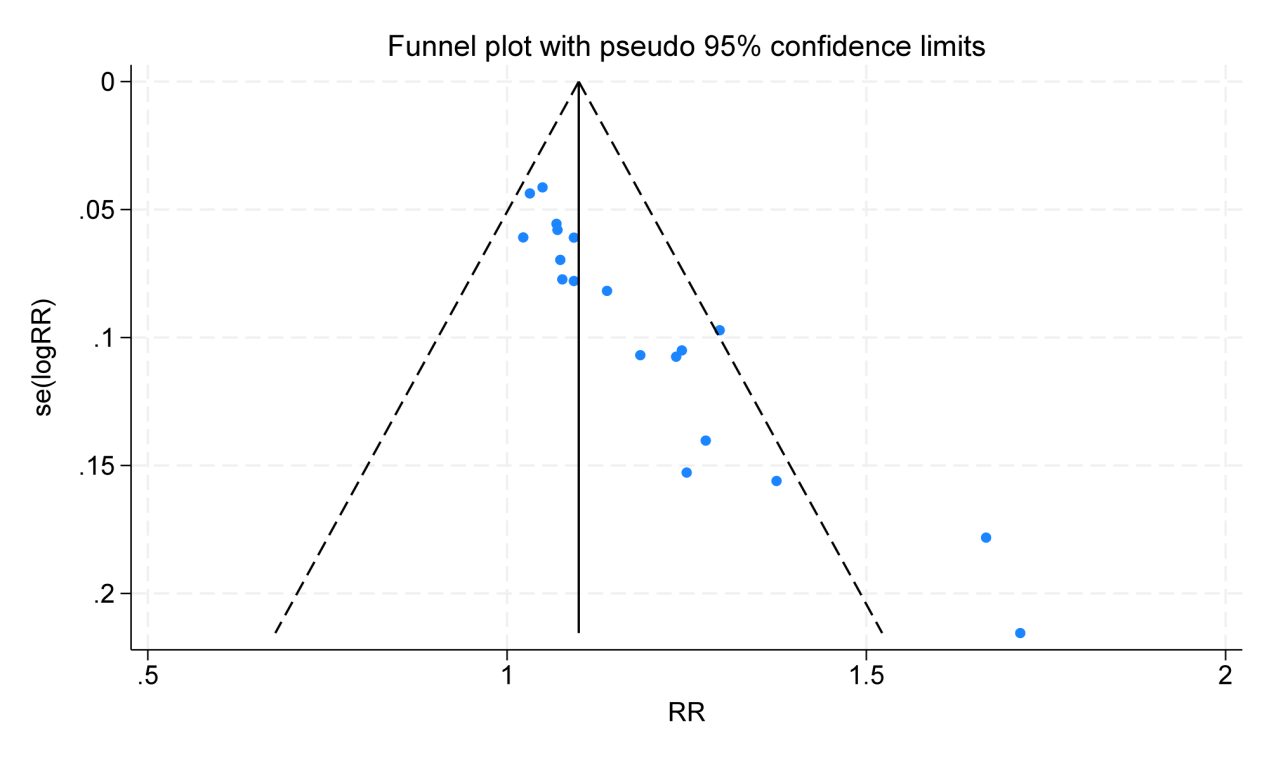


Fig. S4g. Funnel plot for DCR.
